# Current source density mapping of antennal sensory selectivity reveals conserved olfactory systems between tephritids and *Drosophila*

**DOI:** 10.1038/s41598-017-15431-4

**Published:** 2017-11-10

**Authors:** Vincent Jacob, Francesca Scolari, Hélène Delatte, Giuliano Gasperi, Emmanuelle Jacquin-Joly, Anna R. Malacrida, Pierre-François Duyck

**Affiliations:** 1 UMR PVBMT, Université de la Réunion, Saint Pierre, La Réunion France; 2 UMR PVBMT, CIRAD, Saint Pierre, La Réunion France; 3 Department of Biology and Biotechnology, University of Pavia, Pavia, Italy; 4 INRA, Institute of Ecology and Environmental Sciences, Versailles, France

## Abstract

Ecological specialization of insects involves the functional and morphological reshaping of olfactory systems. Little is known about the degree to which insect sensitivity to odorant compounds is conserved between genera, tribes, or families. Here we compared the olfactory systems of six tephritid fruit fly species spanning two tribes and the distantly related *Drosophila melanogaster* at molecular, functional, and morphological levels. Olfaction in these flies is mediated by a set of olfactory receptors (ORs) expressed in different functional classes of neurons located in distinct antennal regions. We performed a phylogenetic analysis that revealed both family-specific OR genes and putative orthologous OR genes between tephritids and *Drosophila*. With respect to function, we then used a current source density (CSD) analysis to map activity across antennae. Functional maps mirrored the intrinsic structure of antennae observed with scanning electron microscopy. Together, the results revealed partial conservation of the olfactory systems between tephritids and *Drosophila*. We also demonstrate that the mapping of olfactory responses is necessary to decipher antennal sensory selectivity to olfactory compounds. CSD analysis can be easily applied to map antennae of other species and therefore enables the rapid deriving of olfactory maps and the reconstructing of the target organisms’ history of evolution.

## Introduction

Olfaction plays a major role in mating and host foraging by phytophagous insects^1^. Unravelling the molecular, physiological, ecological, and behavioral features of olfaction is important not only for increasing our understanding of insect evolution but also for improving insect pest control methods^2^. In *Drosophila melanogaster*, olfaction is mediated by different functional classes of olfactory receptor neurons (ORNs) that are located in distinct regions of the third antennal segment (funiculus) and in the maxillary palps. These ORNs are spatially organized in a one-dimensional array along the ventro-proximal to dorso-distal axis of the funiculus^3–5^. This organization has been documented at morphological, functional, and molecular levels^6^. The olfactory sensilla, which are cuticular units that house one to four ORNs, are classified into three morphological types: basiconic, trichoid, and coeloconic^7–9^. While basiconic sensilla are more densely distributed in the ventro-proximal region of the funiculus, trichoid sensilla are mainly found in the dorso-distal region, and coeloconic sensilla are sparsely distributed throughout. Several studies have explored the functional responses of antennal ORNs with single-sensillum recordings (SSRs), and 42 functional classes have been identified that differ in antennal position and in sensitivity/selectivity to volatile compounds^3,5,6,10–12^. Genetic studies and *in situ* hybridization revealed that each ORN class expresses one or two specific olfactory receptor (OR) genes^13,14^, in addition to the conserved gene of the co-receptor Orco. Furthermore, ectopic expression confirmed that ORs are responsible for the selectivity of ORN responses to chemicals^11,15–17^.

The presence of such an organized antennal olfactory map raises intriguing evolutionary questions: Do all of the regions of the map have the same evolutionary constraints? Could a gain or loss in sensitivity be due to growth, shrinkage, or displacement of subregions in an allometric manner, in addition to being due to changes in the affinity of ORs to chemicals? Does the conservation of OR positions mirror the conservation of OR sensitivity and genetic sequences? With *Drosophila* ^18–20^, true fruit flies (Diptera: Tephritidae) are an ideal model system to answer these questions because they allow comparisons among closely related species that differ in size, antennal morphology, and level of host specialization^21^. Information regarding the ecology of many tephritid species is available because they damage a wide range of fruits and crops and have been accidentally introduced in many regions worldwide^21,22^. Furthermore, transcriptomic and genomic resources have become increasingly available for a number of tephritid species^23–26^, allowing the identification of OR gene sequences. Nevertheless, information on the olfactory system in tephritids is still scattered and limited to a few species. Molecular data are available only for the Mediterranean fruit fly (medfly) *Ceratitis capitata* ^27–29^, the oriental fruit fly *Bactrocera dorsalis* ^23^, the melon fly *Zeugodacus cucurbitae* ^30^, and *Rhagoletis* spp.^24,25^. From a functional perspective, electroantennogram (EAG) recordings have been used to investigate the antennal responses of several tephritid species to the volatiles of fruits and flowers^31–34^, as well as to pheromone components^28,35,36^. With few exceptions^34^, EAG-based experiments have been performed with little concern about the location of the recording electrode across the antennal surface. There is evidence, however, that the antennae of tephritids, like those of *D*. *melanogaster*, are spatially organized. The Tephritidae are, together with the Drosophilidae, members of the Acalyptrata subsection of Brachyceran Diptera, and scanning electron microscopy (SEM) has revealed a spatial distribution of olfactory sensilla on the funiculus surface for *C*. *capitata* ^37^, *Anastrepha fraterculus* ^38^, and species in the Dacini tribe^32,39–41^ that is roughly similar to the one observed in *Drosophila*. EAG signals were found to vary with the relative density of sensilla at the electrode location in *C*. *capitata* and *Bactrocera oleae* ^37,40^, and the spatial organization of ORN functional selectivity on the funiculus surface was reported for *Rhagoletis pomonella* using SSR recordings^42,43^.

The main objective of the present study was to derive and compare antennal olfactory maps of six tephritid species (*Bactrocera zonata*, *C*. *capitata*, *Ceratitis catoirii*, *Dacus demmerezi*, *Neoceratitis cyanescens*, and *Z*. *cucurbitae*). To accomplish this, we used a multidisciplinary approach. We first performed a phylogenetic analysis of ORs based on the sequences currently available in GenBank, and we identified a number of putative orthologs between tephritids and *D*. *melanogaster*. This suggested a partial conservation of olfactory systems at the molecular level. We also developed an innovative method based on EAG recordings at multiple antennal positions and on current source density (CSD) modelling^44,45^, and used this method to map the functional activation of individual antennae. We then used SEM to compare the distribution of olfactory sensilla on the antennae. We found that the functional and morphological organization of the antenna is also conserved between tephritids and *D*. *melanogaster*, but with significant differences among species that were consistent with the allometric growth of funiculus sub-regions. This study revealed that the recording of EAGs at multiple antennal positions is essential for exhaustive chemical ecology research and we suggest that the CSD methods used in this work can be readily applied to other insect species. This study also presents an important integrated approach that should be considered while studying insect olfactory systems.

## Results

### Phylogenetic analysis reveals putative OR orthologous genes between *Drosophila* and teph-ritid species

**Figure 1:**
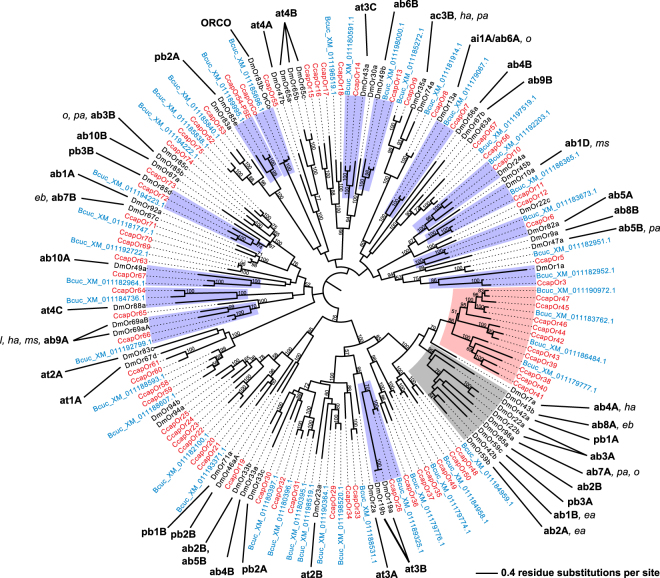
Phylogenetic relationships of OR proteins from *C*. *capitata*, *Z*. *cucurbitae*, and *D*. *melanogaster*. The unrooted maximum-likelihood (log likelihood = −76938.28) tree was inferred using the LG model^72^ with gamma-distributed rates across site (+G) and + F option (LG + G + F). Bootstrap values greater than 50% (1000 replicates) are shown. The suffix –PSE after the protein name stands for pseudogene. For *Drosophila*, we have indicated the identity of the ORNs where OR genes are expressed and the chemicals among those tested in the current study that activate the ORs (consensual-scaled response above 0.5^6^). *Z*. *cucurbitae* (Bcuc) sequences are in blue, *C*. *capitata* (Ccap) are in red, and *D*. *melanogaster* (Dm) are in black. The grey area shows the largest *D*. *melanogaster* gene cluster, the red area shows the largest tephritid gene cluster, and blue areas highlight the 1:1:1 orthologous sequences between the three species, or the 2:1:1 orthology when two duplicated *D*. *melanogaster* genes are expressed in the same ORN.

Odorant reception and processing in *D*. *melanogaster* is mediated by a set of 62 ORs that are derived from an ancestral multigene family^46^. To estimate the divergence between *Drosophila* and *Tephritidae* olfactory systems at the molecular level, we performed an initial analysis in order to identify potential phylogenetic relationships among the OR sequences of *C*. *capitata* (Ceratitidini tribe), *Z*. *cucurbitae* (Dacini tribe), and *D*. *melanogaster*. The phylogenetic relationships of the 76 *C*. *capitata* ORs, the 62 *D*. *melanogaster* ORs, and the 52 putative *Z*. *cucurbitae* ORs that we identified are shown in the maximum likelihood unrooted tree in Fig. 1. Bootstrap support was generally high, especially at terminal branches. The tree is consistent with the known topology of the *Drosophila* OR tree^47,48^.

Most *C*. *capitata* and *Z*. *cucurbitae* ORs were coupled according to their best blast hit and were not clustered in species-specific clades. A partial species-specific clustering was instead evident in *D*. *melanogaster*, including 10 ORs expressed in basiconic sensilla either in the funiculus or in the maxillary palp. This *Drosophila* branch is topologically near a cluster of tephritid ORs including 10 *C*. *capitata* ORs and four *Z*. *cucurbitae* ORs. We also found some ORs that displayed a well-supported 1:1:1 orthology among the three considered species. These include the co-receptor Orco (DmOR83b); six antennal basiconic ORs, i.e., DmOR10a (expressed in ab1D neuron), DmOR82a (ab5A), DmOR49b (ab6B), DmOR13a (ai1 = ab6A), DmOR69a (ab9A), and DmOR49a (ab10A); three trichoid ORs, i.e., DmOR19a,b (at3B), DmOR43a (at3C), and DmOR88a (at4C); and two maxillary palp ORs, i.e., DmOR85e (pb2A) and DmOR85d (pb3B).

### Spatially confined current sources can be estimated from EAGs recorded at multiple positions

**Figure 2:**
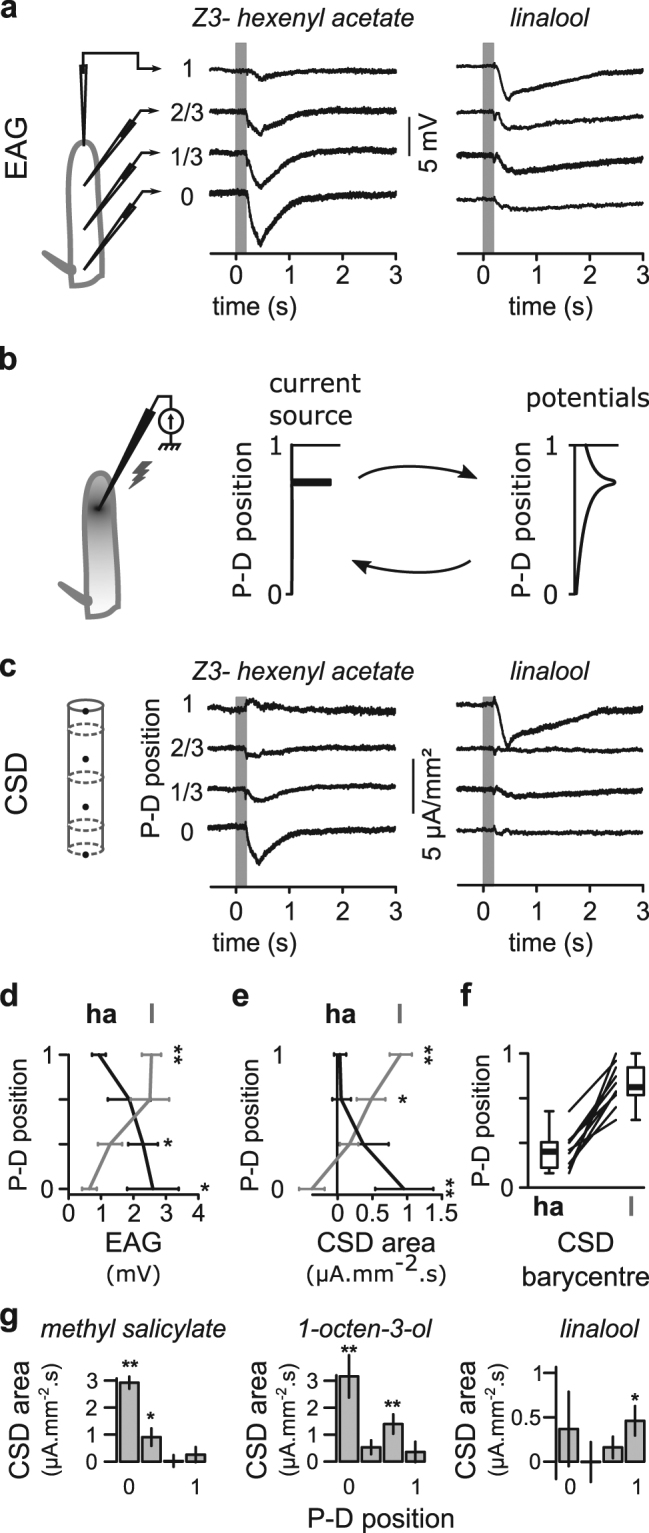
Methods for estimating current source density (CSD) from EAGs. (**a**) EAGs were recorded at four positions regularly interleaved from the most proximal (position 0 at the arista departure) to the most distal (position 1). Middle and right: EAG responses to two volatiles recorded in one individual at the four positions. Grey areas: stimulation time (200-ms duration). (**b**) Diagram and graphs showing that any punctual source of current (stimulation electrode in the diagram and in the middle graph) induces an electric field over the entire antenna (shaded area in the diagram and in the right graph) that can be directly measured. Electric potentials can be estimated at any position if the spatial distribution of current sources is known, and current sources can be localized if the spatial distribution of potentials across the antenna is known. (**c**) CSDs estimated from EAG recordings. Left diagram: the four compartments of the CSD model are represented by grey cylinders, and the electrode positions are represented by black dots. Middle and right: current source responses to volatile stimulations estimated in each of the four compartments from the EAG measures shown in panel (a). (**d**) Mean EAG responses to Z3-hexenyl acetate (ha, black) and linalool (l, grey) at the four electrode positions (error bars: SEM; *p < 0.05, **p < 0.01 Wilcoxon’s signed rank test, n = 10). (**e**) Mean areas of CSD responses to Z3-hexenyl acetate and linalool stimulations. Same abbreviations, color codes, and statistics as in panel (d). (**f**) Spatial barycenter of the CSD response to the stimulations. Boxplots shows the quartile distribution of barycenter positions, and for each individual a black line connects the barycenters of CSD response to the two chemicals (n = 10). (**g**) Mean CSD responses as a function of the position along the antenna for three chemicals in *Drosophila melanogaster* (n = 10, same statistics as in panel (e)).

Odorant Receptors are expressed in different regions of the antennae and maxillary palps and display different levels of selectivity to chemicals. To assess whether the functions of the putative orthologous OR genes that we identified are conserved between tephritids and *Drosophila*, it is necessary to determine whether specific chemicals activate the same antennal region. To accomplish this, we developed a new method for localizing the neuronal activity across the antenna using model-based analysis of EAG signals recorded at multiple antennal positions. Because EAGs consist in recording local field potentials, EAG response should be larger when the recording electrode is positioned near the activated ORNs. In our initial EAG assays, electrodes were placed at four positions on the surface of the *Z*. *cucurbitae* funiculus, and we tested the responses to chemicals that were applied with a puff of air. We observed that spatial variations in EAG responses to a given odor were reproducible among individuals and differed for each chemical tested. Responses to Z3-hexenyl acetate and linalool, two chemicals that displayed very different spatial patterns of EAG activation, are shown in Fig. 2a. The currents generated by ORN activation create local electric potentials that are remotely detected by EAG electrodes. We built a model for estimating current source density (CSD) on the surface of the funiculus from the spatial distribution of EAG potentials (Fig. 2b,c). Current sources were estimated in four spatial compartments along the proximo-distal axis. While the spatial information is already contained in the EAG signals, CSD responses were more spatially restricted (Fig. 2d,e) and presumably localize the activated ORNs. We calculated the spatial barycenter of the CSD responses to Z3-hexenyl acetate and linalool for 10 individuals, and they were significantly different, showing that CSD analysis reproducibly localizes neuronal activation in the antenna (Fig. 2f).

We then determined whether CSD responses identify known ORN positions in *D*. *melanogaster*. We tested methyl salicylate, which is solely detected by DmOR10a expressed in ab1D neurons in the proximal antennal region; linalool, which is detected by DmOR69a expressed in ab9A neurons in the distal region; and 1-octen-3-ol, which is detected by both DmOR85b and DmOR13a expressed in ab3B and ab6A neurons, respectively (Fig. 2g). Methyl salicylate and linalool induced CSD responses in the expected antennal regions, and 1-octen-3-ol induced activation in the two expected positions. We thus concluded that CSD analysis is suited for localizing the activation of single ORs.

### Antennal activation maps are conserved within and among tephritid species

**Figure 3:**
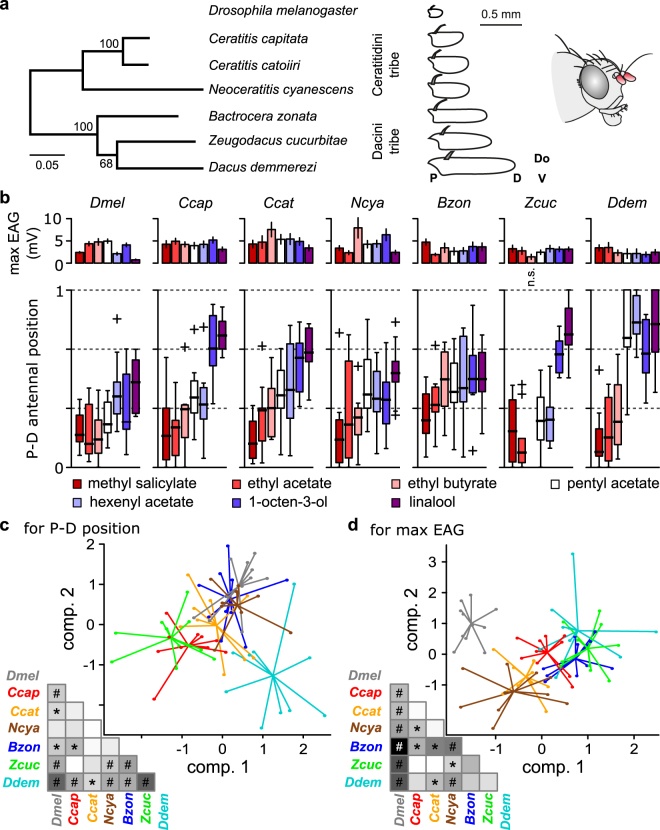
Conservation and divergence of olfactory maps between tephritids and *Drosophila*. (**a**) Left: Phylogenetic tree of six tephritid species. The tree, kindly provided by Massimiliano Virgilio, is a subtree of a published version^49^ and is based on a dataset composed by concatenated COI + 16 S + tRNApro + ND6 + period gene fragments (2167 bp). Methods in^49^; numbers are for bootstrap values; scale bar: 0.05 residue substitutions per site. *D*. *melanogaster* is an outlier species. Middle: Diagrams of lateral views of representative funiculi. Only the proximal part of the arista is drawn. P: proximal, D: distal, V: ventral, Do: dorsal. Right: Drawing of a tephritid head showing the funiculus (3^rd^ antennal segment) in red. (**b**) Characterization of the response to seven volatiles for each species (n = 10 per species). Upper plots: Absolute response amplitude defined as the maximum of the peaks of EAGs recorded at the four electrode positions. Each bar represents the mean ± SE for one chemical and one species, n = 10. Lower plots: Spatial barycenter of the CSD responses to the seven volatiles estimated across the proximo-distal axis of the funiculus. Boxplot: quartile representation (crosses: outliers). (**c**) Linear discriminant analysis for the P-D positions of CSD responses to the seven volatiles. Scatterplot showing the distribution along the first two components. Each dot represents one individual and is connected to the average value for its species. The color code for species (grey *Dmel*: *D*. *melanogaster*; red *Ccap*: *C*. *capitata*; orange *Ccat*: *C*. *catoirii*; brown *Ncya*: *N*. *cyanescens*; blue *Bzon*: *B*. *zonata*; green *Zcuc*: *Z*. *cucurbitae*; and cyan *Ddem*: *D*. *demmerezi*). Lower left: Distance matrix representing the ratio of average Euclidian distance between species and average Euclidian distance within species. Values are linearly color scaled between 1 (white) and 2 (black). *p < 0.05; ^#^p < 0.01 bootstrap comparison of the ratio with 1. (**d**) Linear discriminant analysis for the absolute response amplitude to the seven chemicals. For each individual, the response levels were normalized by the sum of the responses to the seven odors before analysis. Same abbreviations, color codes and statistics as in panel c.

Current Source Density analysis was then used to measure responses to seven volatile compounds commonly emitted by fruits and plants at four electrode positions along the proximo-distal axis of the funiculus in six tephritid species (Ceratitidini tribe: *C*. *capitata*, *C*. *catoirii*, and *N*. *cyanescens*; Dacini tribe: *B*. *zonata*, *Z*. *cucurbitae*, and *D*. *demmerezi*) and in *D*. *melanogaster* (Supplementary Fig. S1). The phylogeny of the tephritid species and their differences in antennal morphology are shown in Fig. 3a. Among the tested compounds, methyl salicylate and linalool selectively activate single ORs in *D*. *melanogaster* that have orthologs in tephritids. Only one *D*. *melanogaster* OR activated by 1-octen-3-ol has a tephritid ortholog. The other four compounds, namely Z3-hexenyl acetate, ethyl butyrate, ethyl acetate, and pentyl acetate are detected by two or more *D*. *melanogaster* ORs with no obvious orthologs in tephritids. We found that all seven compounds induced a significant response in each of the six species, except that ethyl butyrate did not induce a significant response in *Z*. *cucurbitae* (Fig. 3b, top row). We also found in the CSD response a significant interaction between antennal position and insect family for pentyl acetate (F(3,176) = 28.5, p < 10^−15^), ethyl acetate (F(3,176) = 9.5, p < 10^−5^), 1-octen-3-ol (F(3,176) = 8, p < 10^−4^), ethyl butyrate (F(3,176) = 4.91, p < 0.01), and hexenyl acetate (F(3,176) = 2.97, p < 0.05). In contrast, we found no interaction between position and insect family for linalool (F(3,176) = 2.27, p = 0.08) or methyl salicylate (F(3,176) = 0.52, p = 0.67).

The location of antennal response was further determined with the spatial barycenter of CSD responses across the four electrode positions. Distributions of CSD barycenters are shown in Fig. 3b. The proximo-distal order in which the volatile compounds activated the antenna was similar in all six tephritid species. We analyzed the variability of antennal olfactory maps among individuals by performing a linear discriminant analysis on CSD barycenters (Fig. 3c). We also compared each pair of species with bootstrap statistics applied on a distance ratio, defined as the average Euclidean distance of CSD barycenters between species divided by the average Euclidean distance within species (Fig. 3c). We found that antennal maps were more conserved within species than between species. More specifically, three groups with similar CSD maps emerged. In *C*. *capitata* and *Z*. *cucurbitae* (group 1), the activated regions were evenly distributed over the entire funiculus. In *N*. *cyanescens*, *B*. *zonata*, and *D*. *melanogaster* (group 2), none of the tested chemicals activated the distal region of the funiculus, and the pattern of CSD barycenters did not differ significantly between *D*. *melanogaster* and *N*. *cyanescens*. The pattern of CSD barycenters for *C*. *catoirii* was intermediate between those of group 1 and group 2. In *D*. *demmerezi* (group 3), the pattern of CSD barycenters differed significantly from those of all other species in that activity was not induced by any chemical in the central region but was induced by four chemicals in the distal region.

### Position of odor-activated area is not related to antennal sensitivity

We then determined whether the variability of antennal maps was correlated with the variability of antennal sensitivity to odors. Because different chemicals activate different regions of the antenna, an EAG recorded at a single position on the funiculus is biased toward the selectivity of the closest ORNs. To avoid this drawback, we estimated the odor sensitivity of the antenna by calculating the absolute response amplitude, which was defined as the maximum peak of EAG responses across the four recording positions (Fig. 3b). After normalizing responses for each individual, we performed a linear discriminant analysis on the pattern of absolute response amplitudes across chemicals (Fig. 3d) and compared species with bootstrapping the between/within species ratio of Euclidian distances. The outlier species *D*. *melanogaster*, with a small response to linalool and the largest response to pentyl acetate, differed significantly from all other species. The three species from the Dacini tribe had similar patterns of absolute response amplitudes. Among the Ceratitidini tribe, there was no significant difference between pairs of species except between *N*. *cyanescens* and *C*. *capitata*.

### The proportion of olfactory sensilla morphological types varies among tephritid species

**Figure 4:**
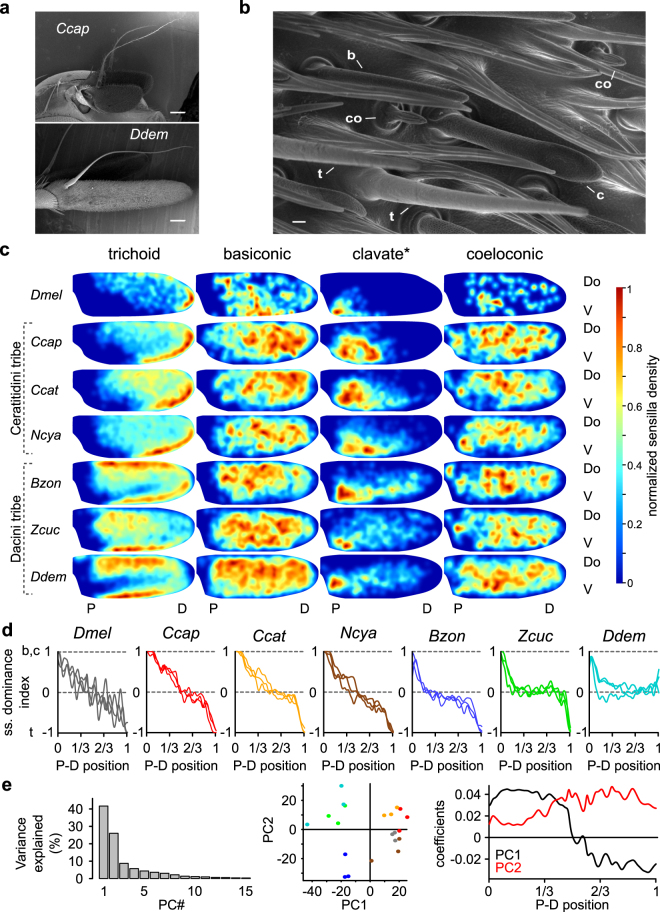
Distribution of olfactory sensilla morpho-types on the funiculus surfaces. (**a**) SEM micrographs of antennae for the smallest and the largest tephritid species. Bars: 100 μm. All SEM micrographs and associated analyses concerned the lateral face of the funiculus (Supplementary Fig. S2). (**b**) Details of the surface of the *D*. *demmerezi* funiculus showing the main sensilla morphotypes. b: basiconic; c: clavate; co: coeloconic; and t: trichoid. Cuticular pores are visible on the clavate sensilla. Bar: 1 μm. (**c**) Average distribution maps (n = 3) of the four main sensilla morphological types for the six tephritid species and *D*. *melanogaster*. *For *D*. *melanogaster*, thick basiconic sensilla were counted as clavate. (**d**) Sensilla dominance index (index = 1 if there are only basiconic + clavate sensilla, index = −1 if there are only trichoid sensilla) calculated along the proximo-distal axis of the funiculus. Each line indicates the distribution for one individual insect (n = 3 per species). Tick marks on the P-D position axis are located at the positions of the EAG electrodes. (**e**) Principal component analysis of the distribution of the sensilla dominance index. Left: The first two components explained 68% of the variance. Middle: Each dot is an individual insect that is projected on the subspace spanned by the first two components. Colors code for species, same colors code as in Fig. 3c. Right: First two components in function of the proximo-distal position.

Variability in the position of odor-evoked activity could be due to variability either in OR relative sensitivity or in ORN location. Because the different ORNs are localized in morphologically different olfactory sensilla, we used SEM to observe the four main olfactory sensilla classes on the external surface of the funiculus (Fig. 4a,b, and Supplementary Fig. S2). Trichoid sensilla were slender, had tapered tips, and had small cuticular pores that we occasionally observed. They lacked a basal socket. Basiconic sensilla were socketed and digitiform; they had blunt tips, were darker than trichoid sensilla, and were covered with large cuticular pores. Clavate sensilla, which are homologous to the thick basiconic sensilla of *D*. *melanogaster*, were similar to the basiconic sensilla except that the tip was larger than the base, giving the sensillum a club-like shape. Coeloconic sensilla were small, grooved, and also inserted in a basal socket. The number and average density of olfactory sensilla for each species are shown in Table 1. Because basiconic and clavate sensilla were easily confused, we pooled them together. The larger species had a larger number of olfactory sensilla but less dense olfactory sensilla than the smaller species. The density of trichoid sensilla and basiconic + clavate sensilla but not coeloconic sensilla differed significantly among tephritid species (F(5,12) = 26.06, p < 0.001; F(5,12) = 7.04, p < 0.005; and F(5,12) = 0.79, p = 0.58, respectively). We then calculated the ratio between the number of basiconic + clavate sensilla and trichoid sensilla. *N*. *cyanescens* and *B*. *zonata* had a larger proportion of trichoid sensilla, while *D*. *demmerezi* had a larger proportion of basiconic + clavate sensilla. The other species had an intermediate ratio of trichoid and basiconic + clavate sensilla.

**Table 1:** Number and density of antennal olfactory sensilla.

Species			trichoid	basiconic	clavate*	basiconic + clavate	coeloconic	total	(b + c)/t
*Drosophila*	*Dmel*	n	90 ± 16	74 ± 15	26 ± 8	100 ± 7	22 ± 5	211 ± 26	1.13 ± 0,13
		*density*	*8*,*669* ± *1*,*828*	*7*,*088* ± *1*,*335*	*2*,*554* ± 878	*9*,*642* ± 836	*2*,*082* ± 456	*20*,*394* ± *2*,*914*	
Ceratitidini tribe	*Ccap*	n	285 ± 27	235 ± 30	119 ± 23	354 ± 35	141 ± 35	781 ± 41	1.25 ± 0,15
		*density*	*5*,*500* ± 901	*4*,*497* ± 471	*2*,*272* ± 389	*6*,*768* ± 261	*2*,*718* ± 744	*14*,*987* ± *1*,*231*	
	*Ccat*	n	365 ± 57	285 ± 73	162 ± 21	447 ± 69	201 ± 75	1,013 ± 200	1.22 ± 0,03
		*density*	*4*,*934* ± 173	*3*,*828* ± 666	*2*,*209* ± 361	*6*,*037* ± 345	*2*,*658* ± 727	*13*,*628* ± *1*,*123*	
	*Ncya*	n	446 ± 49	197 ± 60	212 ± 113	409 ± 66	159 ± 26	1,014 ± 127	0.92 ± 0,11
		*density*	*6*,*758* ± 460	*3*,*017* ± 983	*3*,*169* ± *1*,*517*	*6*,*186* ± 592	*2*,*402* ± 307	*15*,*346* ± 943	
Dacini tribe	*Bzon*	n	642 ± 19	271 ± 65	204 ± 30	474 ± 39	174 ± 6	1,291 ± 63	0.74 ± 0,04
		*density*	*7*,*105* ± 46	*2*,*984* ± 652	*2*,*258* ± 376	*5*,*242* ± 299	*1*,*928* ± 26	*14*,*276* ± 344	
	*Zcuc*	n	439 ± 42	335 ± 51	151 ± 71	486 ± 24	205 ± 76	1,130 ± 63	1.11 ± 0,11
		*density*	*4*,*787* ± 300	*3*,*678* ± 682	*1*,*633* ± 711	*5*,*311* ± 213	*2*,*249* ± 880	*12*,*347* ± 784	
	*Ddem*	n	654 ± 19	656 ± 32	312 ± 155	968 ± 126	391 ± 75	2,013 ± 119	1.48 ± 0,17
		*density*	*3*,*604* ± 194	*3*,*628* ± 469	*1*,*694* ± 739	*5*,*322* ± 557	*2*,*152* ± 380	*11*,*078* ± 338	
p-value		n	<0.001	<0.001	0.17	<0.001	0.002	<0.001	<0.001
		*density*	<*0*.*001*	*0*.*14*	*0*.*28*	*0*.*003*	*0*.*58*	<*0*.*001*	

For each individual insect, the number (n) of each morphological type of sensilla on the lateral face of the funiculus and their average density (n/mm²) were quantified using SEM micrographs. Because the identification of basiconic and clavate sensilla can be confused, we also provide the sum of both sensilla types. The number of coeloconic sensilla is probably underestimated. *For *D*. *melanogaster*, thick basiconic sensilla were counted as clavate. For each individual, the ratio between basiconic + clavate and trichoid sensilla is also provided (right, (b+c)/t). Values are means ± SD (n = 3). The p-value indicates the effect of tephritid species (ANOVA). Same abbreviations as in Fig. 3.

### Spatial distributions of olfactory sensilla mirror the functional maps

We then determined the spatial distribution of the different types of sensilla on the funiculus surface. Density maps were calculated for each sensilla type and three insect per species and were averaged (Fig. 4c). As for *D*. *melanogaster*, trichoid sensilla were absent from the most proximal region, and their density increased on a ventro-proximal to dorso-distal gradient in all species. The densities were highest on the ventro-distal border for the Ceratitidini tribe and on the ventral and dorsal borders for the Dacini tribe. Clavate sensilla were mainly located in the ventro-proximal region, while basiconic sensilla occurred everywhere on the funiculus surface but were rare on the borders. Coeloconic sensilla were uniformly distributed. We calculated an index of sensilla dominance that ranged from 1 (only basiconic and clavate sensilla) to −1 (only trichoid sensilla) and expressed it as a function of the proximo-distal position on the antenna (Fig. 4d). In most species, the index decreased from 1 proximally to −1 distally. In addition, we performed a Principal Component Analysis (PCA) on the sensilla dominance index profiles (Fig. 4e). The first component (42% of the variance) separated the Ceratidini species and *D*. *melanogaster* from the Dacini species, with a regular decrease in the Ceratitidini tribe and a decrease confined to both funiculus extremities in the Dacini tribe. We then determined whether specificities in the antennal structure were related to the three groups that were determined based on the spatial pattern of antennal activation. Species from group 2 (*N*. *cyanescens*, *B*. *zonata* and *D*. *melanogaster*) were separated from the other species by the second component of the PCA (26% of the variance). The shape of the second component indicated that, relative to the other species, these three species have an excess of trichoid sensilla lying mostly in the distal half of the funiculus. The distal region in this group was not activated by any of the tested chemicals. A specific feature of the sensilla dominance index profiles separated group 1 species from group 3 species. In *D*. *demmerezi* only (group 3), the index of sensilla dominance remained at 0 and never became negative in the distal region, indicating that basiconic sensilla were still dense at the distal end. Yet several compounds activated the distal region of the *D*. *demmerezi* funiculus. These observations suggest a match between functional maps and the distribution of basiconic sensilla on the funiculus surface.

## Discussion

In this work, we combined molecular, functional, and morphological data to comprehensively compare the peripheric olfactory system in six tephritid species with known phylogeny^49^ and host range^50,51^ (Ceratitidini tribe: *C*. *capitata*, *C*. *catoirii*, and *N*. *cyanescens*, Dacini tribe: *B*. *zonata*, *Z*. *cucurbitae*, and *D*. *demmerezi*) and in *D*. *melanogaster*. We constructed a phylogeny of *D*. *melanogaster*, *C*. *capitata*, and *Z*. *cucurbitae* ORs and found that potential events of deletions and duplications may have reshuffled the set of available ORs between *Drosophila* and tephritids. However, about half of the OR genes were conserved. Furthermore, we suggest their potential functional conservation because the same regions of the antenna were sensitive to OR-specific ligands in all species. To confirm this, further studies in tephritids would be required including *in situ* hybridization to check the conservation of OR genes expression loci, as well as OR functional studies of orthologous genes to determine if they indeed share similar response profiles. In addition, we observed interspecies variability in antennal olfactory maps that appeared related to the intrinsic structures of the antenna, as revealed by SEM. The link between functional and morphological observations can be explained by the hypothesis that, as in *D*. *melanogaster* ^6^, the set of tested chemicals targeted basiconic but not trichoid ORs. More generally, basiconic and clavate sensilla typically detect food odors, while trichoid sensilla detect pheromones in phytophagous insects^52^. Responses to the fruit and plante volatiles we tested would then be focused in regions with a high density of basiconic sensilla. Interestingly, Z3-hexenyl acetate and pentyl acetate also activated the coeloconic sensilla ac3B located in the proximal region of the funiculus of *Drosophila* ^5,53^. Thus, the differences among species in the morphological and functional maps were mainly associated with the distal half of the funiculus. These differences concerned in particular the ratio between the morphological categories of sensilla, suggesting functional specializations of the olfactory systems.

Studies of *D*. *melanogaster* have shown that polymorphisms in OR genes and related proteins such as odorant-binding proteins affect host preference, are subject to natural selection, and therefore contribute to speciation^54^. The OR family is highly duplicated^46,55^, which favors the emergence of different sensitivities to specific volatiles and which therefore favors speciation. For example, Or22a/b shows an elevated genetic variability among *D*. *melanogaster* populations^56,57^ and among *Drosophila* species^47,48^, and its functional alterations were involved in host-fruit recognition in the specialist species *Drosophila sechellia* ^19^, *Drosophila erecta* ^58^ and *Drosophila mojavensis* ^20^. Whether there is a correlation between polymorphism in chemosensory genes and functional preference of their corresponding proteins has been unexplored in tephritids. Because we found both specific and conserved ORs in *D*. *melanogaster* vs. tephritids, we can speculate about how their functions differ. The 10 ORs in the *D*. *melanogaster* cluster are broadly tuned to small esters and ketones (volatiles frequently found in fruits) and green leaf volatiles^6^. They include indistinctively genes with a high (Or22a/b, Or43b, Or98a) and a low (Or42b, Or59b) evolution rate among *Drosophila* species^47,48^. Curiously in *Drosophila suzukii*, a species that feed on ripening fruits as do the tephritids, the OR genes from the cluster specific to *Drosophila* exhibited higher rates of duplication/loss/positive selection than in the rest of the OR repertoire^59^ and DsuzOr22a might be directly involved in localizing ripening fruits^18^. In contrast, most orthologs of tephritid ORs in *D*. *melanogaster* are narrowly tuned to more complex chemicals: terpenes, aromatics, or alcohols. Confirmation of this categorization of ORs will require the detailed analysis of OR functions in tephritid species and the integration of OR sequences from the other tephritid species involved in this study. This will help clarify whether differences in the conservation of ORs reflect a divergence in feeding preferences or other functional differences between *D*. *melanogaster* and tephritids. Regardless, our study complements similar analyses performed on other chemosensory proteins in tephritids^30,60^ and other diptera species^61^ and provides the basis for future comparative studies that determine differences related to species-specific host ranges.

In addition to OR sensitivity, the abundance of receptor neurons expressing each OR is developmentally regulated^62^ and affects an insect’s ability to detect volatile compounds^19,20^. The two traits could evolve at different time scales. OR sensitivity directly depends on the gene sequence^16^. Within the Diptera, previous phylogenetic analysis revealed no obvious orthologous ORs between species belonging to different suborders^63^, whereas orthologous ORs were found within the genus *Drosophila* ^47,48^. In the current study, we found that about half of the ORs have orthologs between two families (Drosophilidae and Tephritidae) of the same suborder. The functional study we performed shows that the patterns of EAG response amplitudes are more similar within tribes than between tribes, and the patterns therefore seem related to insect species phylogeny at this scale. Because the volatile compounds used in this study are commonly emitted by a wide range of fruits and plants and are not specific to particular host fruits, they are potentially relevant to all tephritid species. Future studies should consider volatiles with species-specific relevance. For example, cucurbit specialists could differ from other species when exposed to cucumber-derived volatiles. We also found that the relative abundance of sensillar morphological types corresponded to the spatial patterns of antennal activation. Differences between both the abundance of sensillar type and the spatial distribution of activation were greatest between two species from the Dacini tribe, namely *D*. *demerezzi* and *B*. *zonata*, suggesting that antennal topography may evolve rapidly and thereby enable rapid adaptation to a changing environment.

Antennal responses to many of the compounds that we tested have been previously observed for *D*. *melanogaster* ^3^, *C*. *capitata* ^64^, *Z*. *cucurbitae* ^31^, or *R*. *pomonella* ^43^. More specifically, five chemicals included in our study were tested with *C*. *capitata* with an EAG electrode located at the tip of the antenna^64^. The response to ethyl acetate was low and the response to linalool was high in the latter study, while we found the opposite when taking into account multiple electrode positions. Regarding methods, our study stressed the importance of recognizing that sensitivity in the antennae of fruit flies is regionalized. We found that the recording of EAGs at a single antennal position in fruit flies leads to the underestimation or otherwise incorrect estimation of the response to some chemicals (Supplementary Fig. S1). Our CSD model predicts that this experimental bias will vary with the geometry of the funiculus: proximal ORNs are more likely to induce an electric field that reach the distal region of a globular funiculus, like that in *C*. *catoirii*, than of an elongated funiculus, like that in *D*. *demmerezi*. As a consequence, the average dimensions of the funiculus (length × width × thickness) for each target species should be considered.

To date, obtaining a functional map of the olfactory system in one insect species demanded significant experimental effort on multiple individuals using SSR recordings. Olfactory maps have been reported in *Drosophila* ^3,4,53^, in the tephritid *R*. *pomonella* ^42,43^, and also within individual antennal segments of mosquitoes^65^ and moths^66^. Although CSD analysis has been proven to be useful for the efficient mapping of functional activity (including olfaction) in vertebrates^67^, it has seldom been used with insects^68^ and had never been used on antennal activity before the current study. We successfully adapted this approach to localize the activated area on the antenna in a reproducible manner between individuals. SSRs and CSD are complementary methods for characterizing olfactory maps: although SSRs provide more precise maps since they have single-neuron resolution and are less sensitive to experimental bias than CSD, CSD is much faster and allows the assessment of individual antennae. The CSD method therefore has the potential to provide a rapid and comprehensive estimation of ORN sensitivity to chemical compounds for all insects with bulbous antennae.

The molecular, functional and morphological evidences we integrated in this paper led to a coherent picture of the olfactory systems in tephritids. Our results suggest that, in spite of the partial conservation of the olfactory system between two different Diptera families (Drosophilidae and Tephritidae), evolution has reshaped antennal topography together with OR sensitivity. This opens new avenues of investigation focused at clarifying the relationships between OR function, ancestral behavior, and niche specialization.

## Materials and Methods

### Phylogenetic analyses

A phylogenetic analysis was performed based on 76 *C*. *capitata* OR amino acid sequences^26^ and 62 *D*. *melanogaster* OR amino acid sequences (downloaded from http://kim.bio.upenn.edu/sofware/dord.shtml ^47^). In addition, the RNA-seq dataset from whole bodies of *Z*. *cucurbitae* adults (available in GenBank; https://trace.ncbi.nlm.nih.gov/Traces/study/?acc=SRP058791) was mined to identify putative OR sequences in this species. In this regards, the 76 *C*. *capitata* OR amino acid sequences were used as queries in tblastn (<1e^−10^) analyses against the *Z*. *cucurbitae* transcriptome. The *Z*. *cucurbitae* hits were then blasted (<1e^−10^; BLASTp) against the nr database. Fifty-two *Z*. *cucurbitae* putative OR sequences were identified, all having best hits to ORs from other tephritids. All of the amino acid sequences used in this phylogenetic analysis are available in Supplementary data file S1. For the three considered species, the amino acid sequences were aligned using MAFFT v7, online version (http://mafft.cbrc.jp/alignment/server/)^69^ with the E-INS-i strategy, BLOSUM62 matrix, 1000 maxiterate, and offset 0. Maxalign (http://www.cbs.dtu.dk/services/MaxAlign/) was used to remove gaps in the alignment. The most appropriate model of molecular evolution for the data set was determined using MEGA 6.06^70^. Phylogenetic relationships were estimated by maximum likelihood with 1000 bootstrap replicates in MEGA 6.06; positions present in at least 75% of the sequences were retained. The resulting tree was drawn using FigTree v1.4 (http://tree.bio.ed.ac.uk/software/figtree/) and Adobe Illustrator CC 2014.

### Insects used in this study

The six tephritid species used in this study (*C*. *capitata*, *C*. *catoirii*, *N*. *cyanescens*, *B*. *zonata*, *Z*. *cucurbitae*, and *D*. *demmerezi*) were originally collected in La Réunion Island. Experiments were performed on sexually mature females at 8–15, 14–24, 7–16, 18–34, 17–30, and 18–34 days after emergence, respectively. *C*. *capitata*, *C*. *catoirii*, and *B*. *zonata* were reared on artificial diet^71^ for 12–16, 162–165, and 131–136 generations, respectively; *N*. *cyanescens* was reared on potato (*Solanum tuberosum*) for 18–20 generations; and *Z*. *cucurbitae* and *D*. *demmerezi* were reared on zucchini for 68–72 and 17–20 generations, respectively. Flies were reared at 25 ± 1 °C and with 65 ± 10% relative humidity and a 12:12 h light:dark photoperiod. *Drosophila melanogaster* mature females, which were subjected to the same experimental setup, were obtained from the standard wild-type laboratory strain Canton Special (CS) reared on artificial diet at INRA, Versailles (25 °C, 12:12 h light:dark photoperiod).

### Odor delivery system

A 7-mm glass tube held 4 mm from the insect antenna continuously delivered a humidified air stream (23 ml/s, air speed 60 cm/s) through a charcoal filter. Stimuli were applied by inserting a Pasteur pipette 15 cm upstream containing a small piece of filter paper loaded with 1 μl of the odorant diluted at 10^−2^ in paraffin oil. A puff of air (200 ms, 5 ml/s) was delivered through the pipette with an electro-valve (LHDA-1233215-H, Lee Company, France) controlled by a digital output module (NI 9472, National Instr., Nanterre, France) and the software Labview (National Instr.). Seven odorants were tested: methyl salicylate (ms), ethyl acetate (ea), ethyl butyrate (eb), pentyl acetate (pa), hexenyl acetate (ha), 1-octen3ol (o), and linalool (l). The odorants were selected because (1) they are commonly detected as volatiles of tephritid host fruits, (2) they cover diverse chemical classes: aromatic, fatty acid derivative, green leaf volatile, and terpenoid, (3) the activation patterns of *D*. *melanogaster* ORs by these chemicals are known, and (4) they activate different classes of antennal basiconic ORs in *D*. *melanogaster* but no trichoid ORs. Odorants were presented every 1 min in random order between different insects, but in a fixed order for different recording positions in the same insect. Control stimuli (1 μl of paraffin oil) were applied once before and after the sequence of odorants. The stimulation sequences were replicated in 10 females per species that were prepared for electrophysiological recordings.

### Electrophysiology

Living flies were secured in a plastic tube, and the head was fixed with dental wax, leaving the antennae exposed. EAGs were recorded with two glass capillary electrodes (tip diameter 1-2 μm, filled with 120 mM NaCl, 5 mM KCl, 1 mM CaCl_2_, 4 mM MgCl_2_, and 10 mM HEPES). The reference electrode was inserted in the right eye, and the recording electrode was leaned against the left antenna without insertion. The signal was amplified (total gain ×200), low-pass filtered (1 kHz) with a DAGAN Ex-1 amplifier (Minneapolis, Minnesota, USA), and was digitized at 500 Hz (NI 9215, National instr.) with Labview software. The position of the electrode was set manually, was always in the lateral side of the funiculus on the medial axis, and varied between 0 (adjacent to the basis of the arista) and 1 (funiculus tip) along the proximo-distal axis. For each individual, four regularly interleaved positions (0, 1/3, 2/3, and 1) were consecutively explored in a random order. For quantifying the EAG response amplitude, the EAG was filtered with a Gaussian convolution of 20 ms width, and response to control was subtracted. Amplitude was defined as the minimum in the 0.5 s following stimulation minus the average value in the 0.5 s preceding stimulation.

### Current source density (CSD)

The location of the current sources generating an EAG signal can be estimated from electric potentials recorded at multiple spatial positions and assuming constant electrical conductivity. For estimating the CSD in the antennae of flies, we adapted the inverse method proposed by Pettersen and colleagues^45^. A point source current *I*_c_ spreads uniformly in all directions and generates a potential *ϕ* at distance *r* from the source given by:

1 $$\varphi ={I}_{c}/4\pi \sigma r$$

where *σ* is the conductivity of the medium. This equation can be integrated spatially for current sources with any geometrical shape. During antennal olfactory responses, the potentials are generated by the dendritic and somatic activity of olfactory receptor neurons located on the surface of the funiculus. Because the amplitude of the signal drops as soon as the electrode breaks through the cuticle, we assumed that the electric conductivity in the internal medium is low compared to the conductivity on the surface and can be neglected. We thus used a simplified 2D geometry with virtually unfolding the funiculus surface. Each current source was assumed to be constant over a rectangular area surrounding an electrode position. In our case, the current sources were estimated at *N* spatial positions, *N* = 4 being the number of recording positions. Given this geometry and equation (1), the potential *ψ*_*ij*_ generated at electrode *x*_*j*_ by current source *C*_*i*_ around the electrode position *x*_*i*_ is:

2 $${\psi }_{ij}=\frac{1}{4\pi \sigma }{\int }_{-q}^{q}{\int }_{{x}_{i}-\frac{h}{2}}^{{x}_{i}+\frac{h}{2}}\frac{1}{\sqrt{{(x-{x}_{j})}^{2}+{y}^{2}}}dxdy\ast {C}_{i}$$

3 $${\psi }_{ij}={F}_{ij}\ast {C}_{i}$$

where *q* is the width of the rectangular current source (half-circumference of the funiculus cross-section), and *h* is the spacing between electrodes. The conductivity *σ* was estimated at 10 MΩ/mm. The parameters q and h were estimated for each species by measuring the size of the funiculus of the left antenna (length, width, and thickness) with a light microscope; the average of 10 individuals was used for each species. The circumference of the funiculus cross section was estimated from the approximation of Ramanujan (1914) for circumference of an ellipse $\pi \,\ast \,(3(a+b)-\sqrt{(3a+b)(a+3b)})$, with *a* and *b* being the two radius of the ellipse (funiculus width/2 and funiculus thickness/2). Corrections on equation (2) were made for current sources at both extremities of the funiculus. At the distal end, the electrode is located at the tip of the funiculus such that there is no neuron beyond the electrode position. Thus, *dx* varied between *x*_*N*_ − *h*/2 and *x*_*N*_. SEM revealed that almost no olfactory sensilla were located more proximal than the arista where the electrode at the proximal end was located; as a consequence, *dx* ranged from *x*_1_ to *x*_1_ + *h*/2.

The EAG potential *ϕ*_*j*_ measured at position *x*_*j*_ is ${f}_{j}={\sum }_{i}{y}_{ij}$. In matrix formulation:

4 $${f}_{j}={F}_{ij}\ast {C}_{i}$$

The coefficients *F*_*ij*_ were calculated directly. The best estimation of current sources *C*_*i*_ when the potentials *ϕ*_*j*_ are known is then given by the reverse formula:

5 $${C}_{i}={{F}_{ij}}^{-1}\ast {f}_{j}$$

The CSD was estimated at each time point surrounding stimulation, and its area was calculated between 0 and 1.5 s after stimulation.

### Scanning electron microscopy

Flies were placed in 70% ethanol and sonicated for 5 minutes to remove surface wax from the cuticle. Heads were carefully excised, dehydrated in 100% ethanol, dried in a critical-point drier (CPD7501, Quorum), mounted on aluminum stubs with argent glue, and oriented so that the external side of the funiculus was exposed. Three specimens per species were coated with platinum (7 nm thickness; EM ACE600, Leica, Germany) and examined with a GeminiSEM 500 microscope (Zeiss, Germany). Olfactory sensilla on the surface of the external side of the funiculus were counted, localized, and assigned to the four main morphological classes (trichoid, basiconic, clavate, and coeloconic sensilla). The number of coeloconic sensilla is probably underestimated since some of these small sensilla may have been hidden. To generate sensilla density maps, antenna length (from the arista to the tip) and width were normalized. Sensilla density maps were computed with convolving spatial positions of sensilla with a 2D-Gaussian Kernel of standard deviation equal to 2% of funiculus length and 5% of funiculus width. The sensilla dominance index was defined as (D_b_ + D_c_ − D_t_)/(D_b_ + D_c_ + D_t_) with D_b_, D_c_, and D_t_ being the average density of basiconic, clavate, and trichoid sensilla, respectively, along the proximo-distal axis.

## Electronic supplementary material

Supplementary figures and data
