# Supplementary material for: Current source density mapping of antennal sensory selectivity reveals conserved olfactory systems between tephritids and Drosophila
Source: Sci Rep. 2017 Nov 10;7:15304. doi: 10.1038/s41598-017-15431-4 (PMC5681579; doi:10.1038/s41598-017-15431-4)
Supplement: Supplementary file 1 — Supplementary figures and data [file 41598_2017_15431_MOESM1_ESM.pdf]

Supplementary information for

Current source density mapping of antennal sensory selectivity reveals conserved olfactory systems between tephritids and Drosophila

Vincent Jacob*[1,2], Francesca Scolari[3], Hélène Delatte[2], Giuliano Gasperi[3], Emmanuelle Jacquin-Joly[4], Anna R. Malacrida[3], and Pierre-François Duyck[2].

Supplementary Figure legends

Fig. S1. EAG responses and CSD estimations. Top: EAG responses to the seven chemicals tested at the four electrode positions. Significant differences from control are indicated by asterisks (**p<0.01, * p<0.05, Wilcoxon's signed rank test, n=10). Bottom: Mean area of CSD estimations. Values are means $\pm$ SE, n=10. ms: methyl salicylate, ea: ethyl acetate, eb: ethyl butyrate, pa: pentyl acetate, ha: Z3-hexenyl acetate, o: 1-octen-3-ol, and l: linalool.

Fig. S2. SEM micrographs of antennae of the six tephritid species. Top. Whole antennae of the six species. Bars: 100 μm. Bottom: Details of the funiculus showing the main sensilla morphotypes for the six species. The micrographs in the bottom are presented in the same species order as in the top micrographs of the whole antennae. b: basiconic; c: clavate; co: coeloconic; and t: trichoid. Bar: 1 μm.

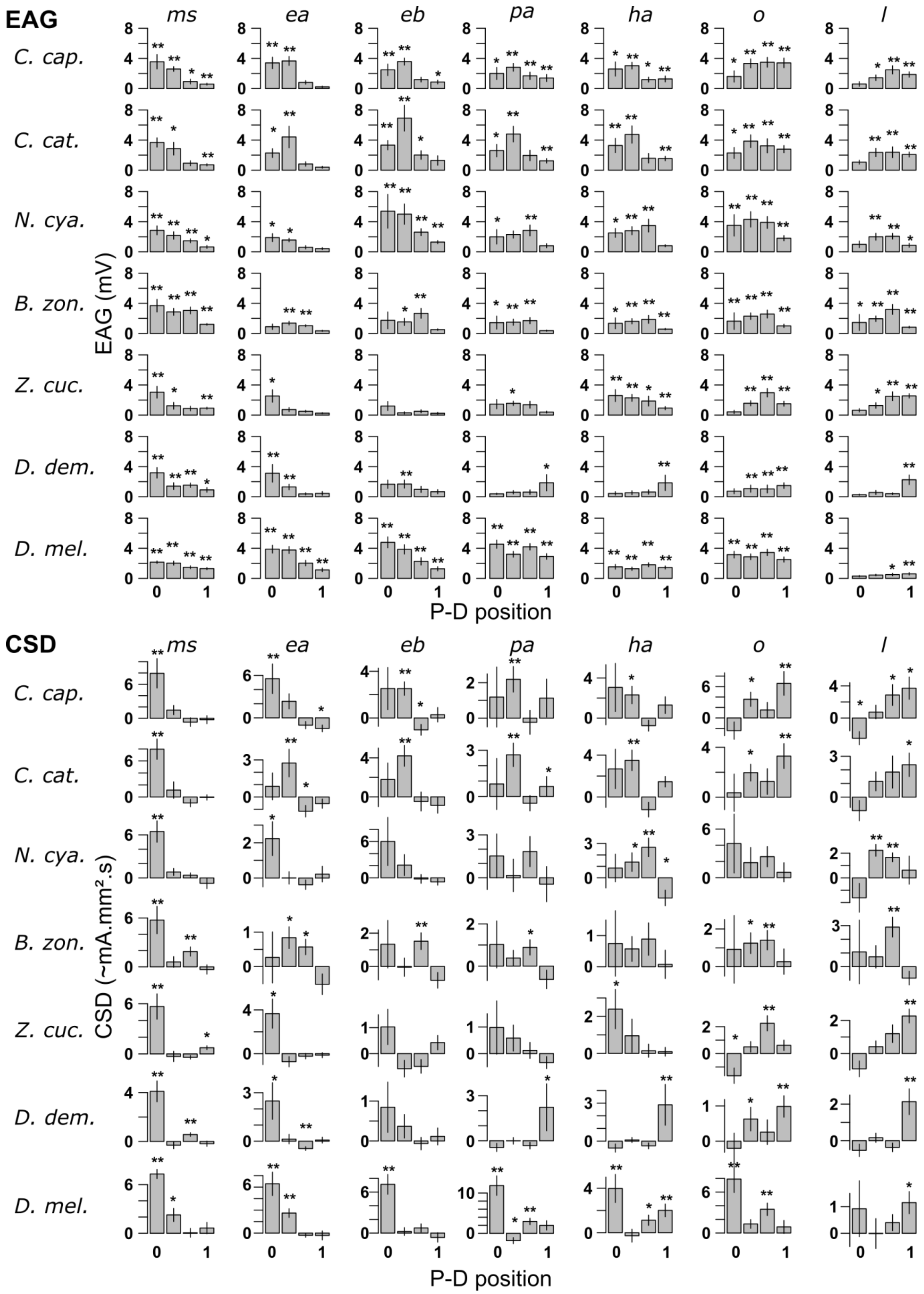

Fig. S1. EAG responses and CSD estimations.

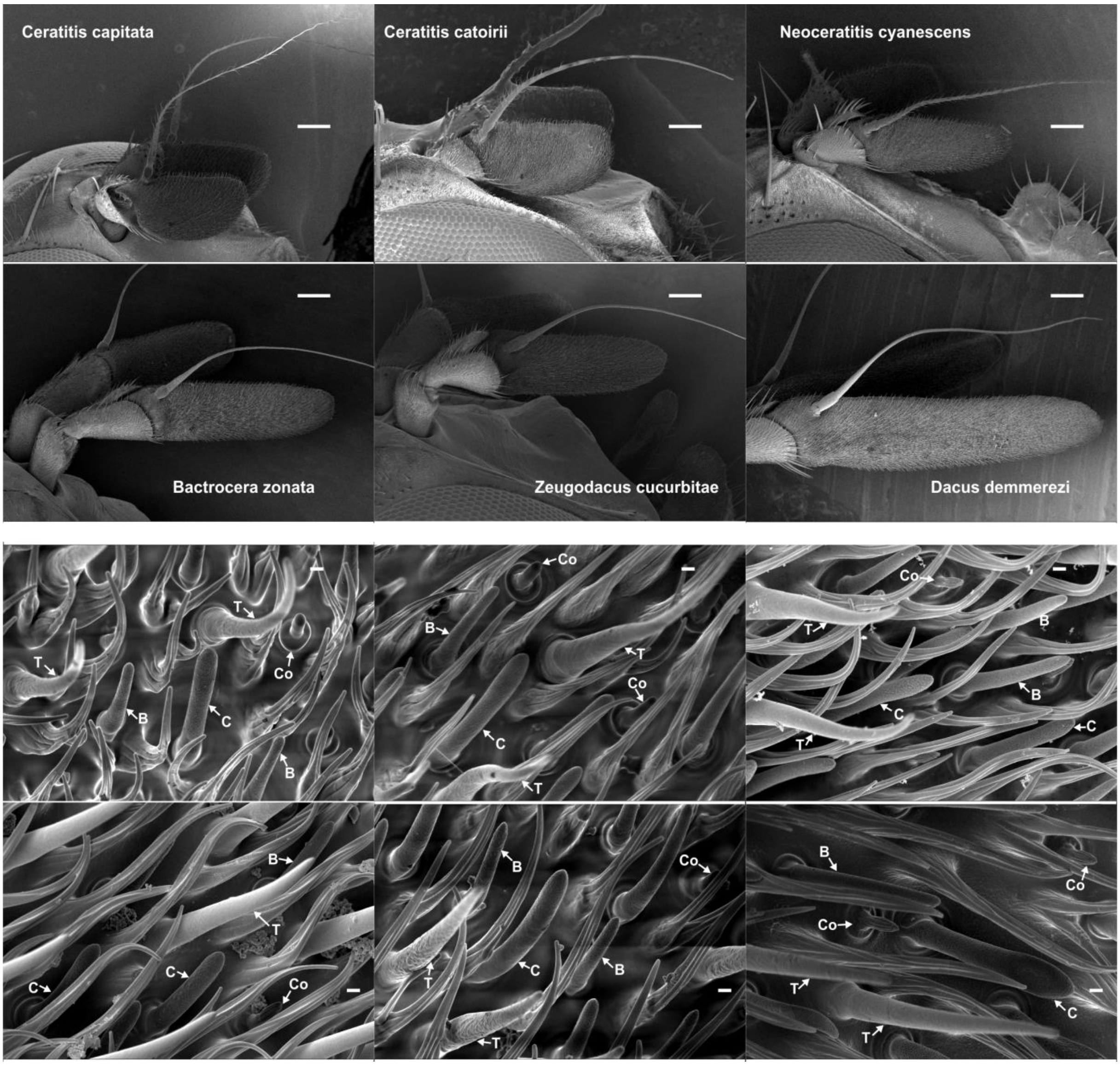

Fig. S2. SEM pictures of antenna for the six tephritid species.

S1 Data. ***C. capitata*****,** ***Z. cucurbitae*****, and** ***D. melanogaster*** **OR amino acid sequence list.**

>Bcuc_XM_011185696.1

MQPSKYVGLVADLMPNIRLMKYSGLFMHNFTGGSGLFKKIYSSIHLVLVVVQFLLILVNLALNAEEVNELSGNTITVL
FFTHSITKFVYLAVSQKNFYRTLNIWNQVNSHPLFAESDARYHAIALAKMRKLFTLVMLTTVATAVAWTTITFFGESV
KFAVDKETNSTITVEIPRLPIKSFYPWNAGAGMFYMISFAFQCYYLLFSMVHSNLCDVLFCSWLIFACEQLQHLKGIM
KPLMELSASLDTYRPNSAALFRSLSANSKSELINNEEKEPTDLDISGVYSSKADWGAQFRAPSTLQTFNGMNGTNPN
GLTRKQEMMVRSAIKYWVERHKHVVRLVAAIGDTYGGALLLHMLTSTIMLTLLAYQATKVTGVNVYAFTTIGYLGY
ALAQVFHFCIFGNRLIEESSSVMEAAYSCHWYDGSEEAKTFVQIVCQQCQKAMSISGAKFFTVSLDLFASVLGAVVT
YFMVLVQLK

>Bcuc_XM_011181431.1

MDKLAVLSSRIFPSDPSKGKIGSIEYNVWLAQLFGVPVVGLKAESPLMRIALGIYGVLLTLLVTFIYTGFEIYDMILCWP
NLDDLSQNICLSLTHIAGVLKVINILYRLDEVAHVVRRIEYSAKTYVISKRQLVAFYRGEFENKIPLTIYASLVGFTGVLGII
YLFYNPVGVAGQIFPYRVKLPDWMPFGLQLAYMGISVLVFALQIVSIDYLNVTMINQIRFQLKILNLAFEELKLDCGS
KKKAHFNHDKRLQTIVEHHCLLRDLRIDVEEIFRMPVLLQFFTSLIIFAMTGFQAIVKTENSNGAALIYCYCGCIFCELF
VYCWFGHEVSEQSKTLTTSGYSSHWFEFDQRFKKSLLIFMCNSQTPFVFTAGGFMSLSLPSFTGILSKSYTVIALLRQV
YSR

>Bcuc_XM_011182952.1

MRITILKDIKPITDAFVRIGLSIELGGSKGIFSHPERYATVMIGVIVWAVALFTYTMEYLADVDKIVAAMTINVQLCLTT
SKNFIFLARRERFLRLNEALERLALTGNNIERELWNTSNRRVLPITMAYSISCQMTVSICVLLPILKLLYYYIWHNEVVLT
LPLPGIFPYDYTVPFYFILTTILSVLLVYFCVYTICAVDGLFGWFVYNISAHLQIMRLKLEQLLQLHVDDPNFQRDLVAL
VNYHRQIIDLSLELDALYAPIIFLEVTSSSLPICFLAYQLSYLSDPASVPFMCLLMSSIVIQLMIYCFGGEKVQNECDQLC
ENIYLLIPWHKLPPKHCRLLLNPFIRSQRVLVLTGYFFTANRSLLVWIFRTAGSFTALLFALKEKEV

>Bcuc_XM_011182951.1

MTTREARVTQICFLIIQIFALASILIPIAVYSWQHIDDIAEVSNAMAPFMQATISLWKVWRVIYRRKEMAELCENIYLIS
AKASKLELAHLIQENNRERIMNTAYYYSVLNTGVMALTAPVVVSFIQYLRLGEFSYITALKATYPIDYARPLNYFLIWL
WTAVAIYGVIYVSVPVDSLYSWYIHNLVGNFKILQSKLVSAESIAESTADLGKRRELIYYCVAYHQRLITMSEQLNIIYQ
PIVFVQFSLNGLQICFLAYQIGSGVVAMVDLPFLLLFLISVGIQLMIYCYGGQHLQNESVNVSKSIYQTINSSPWPNEL
RKVLLISLMRAQKPCKLTGIFFDVDLPLFLAVWRTAGSYVTLLRSVDQKTM

>Bcuc_XM_011183673.1

MPEDLFRIQRNCLRLMGHQDIYDDDNETAGDEQKSKSWQQRCFRHSQTLKYALLLLLMMSAQLPMMDYIIYHID
DLALATACLSIVFTNVLTVIKTSTFLAYKREFKSLMAEFERMYDELHEAGAKRCLVTVNVGAKRFVKLYFYSVSCTGLY
FTIKPLVSMIWAKFQTKPLVLELPMPMRFPFDFESPPGYQFAYIYTILITIVVVMHATSVDGLFVSFTTNLRGHFQAL
QYFIETNTYDKSDALIQRELRIYVHYHVRLLELSQSVQRIFKPIIFGQFLMTSLQVCVIIYQLVTNMGVIMEMVVYCTFL
SSILLQLLIYCYGAEFLKIESSAVGTAVQMSQWYNLPPRHRHVLRLMMLRSQREIIISAGFYEASLANFMSILKAAMSY
ITFIQSIE

>Bcuc_XM_011179067.1

MLFNPKPLKDPISFRFPLQCIWLKLNGSWPLRPQRSGEFERYFRWLYSVWAWYVVAMVGITIGFQSAFLVKTFGDI
MVTTENGCTTFMGVLNFVRLLHLRLHQREFQQLLARFVKDIWITSSSQPIVERACARNMRVFQVISALQSCLITMYC
LLPLVELYMLTVNTAPDVLESVEKPFPYKMLFPYDANYGWRYALTYLFTAWAGVCVVTTLFAEDSLFGFFTTYTCGQ
FRILHIQIDNIIPDSYAATRAGRGTEADFQRESVRRLDRIAGKHCVLFNFVRSMEEFFSPILLVNFLISTILICMVGFQLV
TGKNMFIGDYVKFLVYILSSLSQLFVLCWNGDNIIQNSLEMANHLYACNWECGVEVAATNADNKRADEQVKMAA
PTVYYTTNNAFRKKLQFMIMRSQRQTCITALKFSVLSLSSFSGLISSSMSYFALLQSFNEDEEN

>Bcuc_XM_011181914.1

MVTIAQFMSESMVLIGEGIVMHDNLDNISFVCTVLAPNLILIEMMLRAYNIIYRRSSFRKHIEEFYKKIYVQRTWNPD
LFEQIRRQQLPTKYSTCTYIITLVTYVYVPISGLIKNERLVPFPIRFSFDYTVPWPRYLVFLAMSIWTGFAVVGPLVAEPN
LLAMQILHLNGRYSLLLQDLRKISKESIVEHERLKGKDTLLVTQRFRYRLFEIIRRNVELNEFAKSLQEQYSFRVFVMM
AMSATLLCVLGFLTATLGLTAQNIRFVSWIIGKVVELLIFGRLGTTLSTTTDELSTSYYCCDWEDVILHSTDAEENKKL
MKLIALAVHLNSNPFRLTGLNFSVVNYETVVSILRGAGSYFTVIYAYR

>Bcuc_XM_011185272.1

MLYRPRLNNGKLIPLSWPIVAYRLLNNICWPLRDHASLLERLFDRFFWSLGFFIFVQHNDAELRYILVNNNNLDEMLI
CGPTYLILVEIHLRAFQLGLNKEAFKRFLQKFYAEIYIDEPAHPKLYASIQKRLRPIWFYSLLYFSTLSSYVIMPFVNYLNN
VKAPLYKMYYPFDITPNPIYVAVVLSNIWVGFTVITMVSGEDNILSEVMLHLNGRFLLLQQKLGQDAERLLHAGDDR
NIADDLRQRIVEAIEENVRLYKFAEDFEREFSFRLFVSLSFSAGLICVLGFKVYTNPMASFGFMFWICAKVMEMLLIG
QLGSSLIYTTNEISSTFYKSHWELVIQKSSDTNANVRLMKTLLLAITTSQKPFVLTGFNYFSVSLTAVLKILQGAGSYFTF
LTSMRK

>Bcuc_XM_011192203.1

MLLKFLSQNYPTEKNVFLIPKFALRIVGFYPGDSKSRLMHAWLIFNMFVLVYGSYAEFMFGVHYLSIDVVRALDALCP
VASSIMAIVKLSFLWWHRAELNCIIKRVAELTAEQKSPLKSYYKHRYFTTATRLSAAVLCFGTTTSTLYTIRAAMVNYS
SYLREEKIPYETPFKMIFPQPLLSMPIFPLTFILSHWHGYITVAGFAGTDGLFLCFCKYIGTLLKALQFDMKDLLSDVDS
VTRKSTSEYEFRESLKLIIARHNEIIDLVKRFSAVMSGVTLAHFVTSSVIIGTCVVDVLLFSDLSGIFVYSVHTMAVTSELF
LYCLGGTVVIECSSQLATAAYDSQWYTHSVEVQRMVLLIIIRAQRSLVVKVPFFAPSLPALASILRFTGSLIALVKSVV

>Bcuc_XM_011186365.1

MNFRFLSRTFPLRDYYFYVPKLCLGALGFWPLDTCEPGAFNVWAWVNLIILTIGVVTEMHAGCLALRTDLELALDTL
CPAGTSAVTLLKMALIYYYRQDLAWVLKRMRDLVYERDGELLANLEKHIVRAHAVMAARLNFIPFVMGFITCTSYNL
KPLLMTLILYVQGREPMWKLPFNMTMPPFLLRAPYFPLTYIFTAYTGYITIFMYGGCDAFYYEFCSNTAALLELLQND
LKSILSFGGDKFTLTAEESTVLEWRLVQFIKRHNDIIELTRFFCKRYTVITLAHFVSAGLVIGASIFDLMTFTGFGIVIYIGY
TIAVLGQLFIYCYGGSMVAESSVQLATVAFGCDWHACNPRLRRYVLMIIIRSQRAISMSVPFFSPSLVTFTSILQTSGSI
IALASSFK

>Bcuc_XM_011195190.1

MRRLLGPQVPIERSFFRIPRFSARIAAFWPQTFNRSLYWLTALRFCVNTFAVAVGGIGETLYGFVYLHDLFSALEAFCP
GITKVISLLKMTIFFVRRKRWLHVIDGMRQLLLLDTSAEKRRIMEPLASFGSVLSFVLLISGSVTNTFFNILPLLKMGYY
KSQSLDVPLLLPFNVILPEMFVNWPYYPATYLVLTLSGAMTVFSFSAVDGFFLCACVYTSALFRILQHDIRGAFAELQE
QEHSTLVQNMRIQHRLAVLIERHNKIIDLCSDFASEFSLIILMHFLSASLVLCFSILDLMLNSTSIGVLTYIFYSIAALTQLV
LYCIGGTYVSESSLKVAEVIYDIDWYKCDVRTRRMLLIMICRSQKAKTIQVPFFTPSLPAFRS

>Bcuc_XM_011198000.1

MFDDLQLIHMSVRILRFWSLIYEHTWRRYVCLSMTTFLVFTQLYYMFRTSEGIVSIIRNSYMLVLWFNTILRAYLLLYD
REKYEKLLSDLEKFNFDLKQSKDFYIQDLLAEVNKTGKYMAQGNLFLGLLTCFGFGFYPFFVNERVLPFGSMIPGVDE
YKSPFYEFWYFYQMIITPMGCCMYIPYTSLIVGCIMFGIVMCKALQFRLKTLHRVRHDEPLIRKHVRECIRYQLCIIDYI
ARVNSLTTYIFLLEFLAFGTLLCALLFLLIIVSGLSQAIIVCAYIAMIFAQILSLYWYANELREQNLAIAAAAYDTEWFTFPI
PVQKYILLMILRAQKPPAIMVGNTQPITLELFQSLLNASYTYFTLLKRVYT

>Bcuc_XM_011180591.1

MSTAVEDNPLLSINVRLWKSLSVLFARDWRRCVAFVAPICLLNAMQCVYLYQQWGDLATFILNTFFAVSVFNALLR
TCLIIKNRDKFEALMEELVTLYDNIEESGDDYAKSVLAAATKSARNISIFNLSASFSDLIVAMAYPLFQEQRIHPFGVAL
PGIDVTRSPLYEIIYIGQLSFPFTLSSMYMPYVSLFASFAMFAKATLQILQNNLKNLCDNMKTKTEEELFELLRTNIAYH
ARIARYVSDFNELVTYIVLIEFLLFSCVICSLLFCINIVSTTTSTAEKISIVMYIGTMLYVLFTYYWQANGILEMSLLVSDAA
YEMQWYNCSPRFKRTLLIFIGRTQKPLQIRVGQMYPMTMEVFQSLLNTSYSYFTLLHNLYND

>Bcuc_XM_011188726.1

MYKSQAFLDFFVFVEKWYEEQQRDGEEVTLRKTHEFTQKIRKAAKTLLIVTMIILSYMVLIQLLATVGIGYRKLIVDVA
FPGIDFYISPNWEMMSFLQCLYVAPFTFVSYVSYLCLTLIAISFGIFLMKDLQFKLENMNDMTDLEALNCIKKCVKAH
VMIIKYHNHLEALFSVGSFADVCIFGIIPCVIIVLSTMDHDISMLIADIQMAAMVMTSTFIFFWVANNFVIESENIANA
AYNCNWVDRDKEFRKYIPLIIGNSQKPLQLTAGGIKPINMEFFLTIVRCTYSLFTVLFTMKTGGDS

>Bcuc_XM_011196519.1

MASYQNLPLYSVNIKAFVKLGLIESNNSTRRFLLGIIIIVTYIGQLTNMFRTWDVDIGETGMNFHVLALVTHYLLRFIIIV
RKEKKFERLFQGIEPWYTDIERHGDPHIVSILQKITQKTQRLTRLSFYASVVATLATFIYSLSFDERRLLVTVQYPFFDVL
QTPFFEFFFLIQMVWLVPTSLLVYLSFTNIFLTSLMFGELILKDLCLKLRNIRSENEMTMLKEFKDCIAYHNKIIDLCGDI
EDLISMDAFFHVTSFGMMLCMLLFFLSMIDDLELIPAVLVMMGFDMYLIGFSYYYANNLATESLEVANAAYDTPW
YRGNLEMRKCVLIMIARSQNPLQITAGGLYPLTMENFQAILRISYSYFSLLQGVSQQ

>Bcuc_XM_011195121.1

MDDNMINRQLKDCIQHYVKLRNITEIMEDLLSIPMSVQMMSSVLVLVANFYAMTFLTDPSDYVTFIKFLVYQLCML
SQIFMLCYFANEVSLRSAELSYSLYSSEWTRCSQINRRLMLLMMAQFDVPMRIKTINRCYSFNLPAFTSLSKF

>Bcuc_XM_011193371.1

MAIDTMVNFRTIVRILTLSVTFGFILLLSLEIIYSDGLDHAIDVLKYLLVVMALGIKVLNAWYYTRQITEVMYEWENSEL
FVLRNDDEKQMWAKTQKTFRKLGMTSFGLGFNSAMCALLGVLLMGATEQPYALWMPTNWRDKYYWQMYIYQ
CLSMPFICFSNVTNDVFQAYLLLHLTLCFRVISMRLERLANAGADGAITAELMNDIKMHQRVKEMAISCEHIISLSML
TQITLTFLIICFIIYNLANANFREDPVHCLAMLQYALIVSLQMFLPCYYGNELTLESEKLSINLYSSDWTGMSAYNRRFIF
HYMESLKKPLVLHAGSFFEIGIPIFAKAMNNAYSLLALLLNVNDDEQ

>Bcuc_XM_011182100.1

MKLEQFDNISGGRRVIKILKLLGLWHYGGVFRMPYLLYSGLLHSSFTIPYTIMMCLDVVQASNLEKFTNTMYMTLTE
LGLVAKLVNVWIYSRLLVDFFAAFTNDKLYKLQDAEEQLSWRRTQGNYARIAFLYFAMSLGALASAFVGVLYSEDYE
LPFPYAPPFDWRTPRGYWYAYFYELLAMPVTCLSNCAFDMIQCYMLLQLSLCFKLISGRLERMGALREDSASRGFSE
FRLHREFVDIVRLHARTKKLSQQCQTYISFPFLIQIICSSFVLCFSAYRLQKVPIQENPTQFLTLVQANLIMVLQIFIPCYC

GNEIIQHSSGLNNATYNAEWFRCSPKMRKYLVIYMEMLQRPVRVRAGDFFDISLAIFTKTMNNTYSLMALLLNMN
K

>Bcuc_XM_011189325.1

MIVITLLFPIYYPTCFTVESLLADNLNDFCEVIYIAMADVTLNIKFLTLFIVRQQLLELRPILKRLDARAKTEEEIGVLQDGI
DSAKKCFLIILRLFYSAFVTSQLMVIFSGEARLMYPAWYPFNYKATRTKFWIAYGYQTIGFLVQCTQACSVDTYPQAY
MRVLTAHIRALSLRIEAIGRKSFNCDSSEFIPLTKDEMKRNYDELVSCIKDHKTIIELFSTIQKPISGTAMAQFVCTGVA
QCTIGVYMLYVGFNISIMLNMAVFFVSVTMETLILCYYGDMFCQECEQLSKAIYNCNWTVQSSEFKKALCFFLLRSQ
RVNVLMAGNWIPVRLPTFVMVVKSSYSIFTLLSSFK

>Bcuc_XM_011198519.1

MNNSPPVDSRQFFRTHWRLWLLLGCVREPVHYQLLYRLYSTVVNALIMLFYPGTILIALYNSANLTDFLQTLPICAAA
LACSAKYISYYRRLGLVRQAEQIFNALDEQVLLPEDREFYAGIHRGTNLILNTLRGLCIFFMAITVMAFASSIEERGLAF
AIELPFDWRKSSVAYVGAVGLELLLLSCDLLQSLANDSFPAIALCVLSNHTRLLGARLARIGHTSKDVQANIREMQRCI
IDHQRLYRLQAIIEEIISMPVFIQYAVTAFQDCFTLITFIFYTNTVSDKVLYLTYLLALQLQIFPTCYYGTACAQSMDDLQ
QEIYASNWVEQNQVYRRLVTIFSQRTLKSTTTYAAGLIPIHLSAFVKTLQGAYSFYTFVDGVRKV

>Bcuc_XM_011190364.1

MSPSPLSLPPPPPPLAAVDTRSFFKLHWTCFRVLGINAPSSNTYYSVLLQVLVTLCYPFHLALALFSSPDASINIQNLTV
CVTCVACSMKFVFYATRLSRIRELESIIAALDARARSLCERRYFVQLRKELRRITICFLCIYTVVGVTAELMFIFHNERNLL
YPAWFPFDWRASNLKFYAAHSYQIVGISYQLLQNFVNDCFPTMALALLSAHIKLLGIRVSQIGHETASLGANEAELLH
CIKDQEQLYNVLNIIQNIISLPMFLQFTVTAINLCLGMAALLYFVDAPFDRLYYLAYLLALPLQIFPICYYGTTFQLLFDKL
HVEMFASNWVEQTHKFRKHMILFCERSLMSQTAMAGGIVRIHLDTFISTCKAAYSLLAVIMKMNE

>Bcuc_XM_011198520.1

MNQPINSNVFFKIHWLGFRICGGDSTVNKYRLVYLAYALMVTVLVTVCYPLHLALALFRNGSLGGNIKNLAVCVTCI
ACSSKFLIYTRKLRIMHEIEQTFAELDARVSSEVERKYFARLRISVRNVVSVFVCAYAAVGMTAELAFLLSKERSLLYPA
WFPFDWRASTRNFYVANVYQFVGISYQIFQNFIDDTFPPITCCLLSGHIKLLGIRVSRIGYDCVNRQDNERELVRCIKD
QKNLYKLFVLLQEVMSWPMFIQLTVTAFNICVAMFVMLFFVDTPFERLYYLVYFVSMPLQIFPICYYGSSLQHLFSQL
QYDVFRCNWTDQTPRFKKQMMLFTERALKTTTALAGGMIKIHLDTFFATVKGAYSLFAVIMKVK

>Bcuc_XM_011180397.1

MSSSNPSLQSVNSVVLYREFWLCWHAVGISTAYQKHLCALYDLLINVLVTIFYPIHLIVGLFLNPTPADLFQNLSITITC
FVCSVKHYLLRRKLPQIRVVQALLADLDKRVEDAEERAYFEKQLVVGAKNVVKLFSIAYGGANMAAISATLLSKERRL
MYPAWLPFQWQASTFSYCAAVIYQIAGVTIQIVQNLANDIYPPMSLCIIAGHVHLLALRVAKVGRDGKKSLKQHNQ
SLIECIEDHKKLVRIFELTQETLSQAQLAQFISSGLNMCIVLFYLIFYVDNVFSYIYYAVYFVSMAIELLPSCFYGSMLIYEF
QQLPSAIFKCGWLGQSREFYQNQRIFVQLTLKEIVPLAGGVIGIQLDSFLGTCKVAYSLYTVCNRMK

>Bcuc_XM_011180395.1

MVRQLDTRAIFTRLFLTWRVLGIIDWPFHRHLRLVYDILMNTVVTFGFTAHLVLGIILSTNQDQFFTNLVIGIASVSCV
FKHLLYRFRMPEMQRINEILGQLDDRVRTKEDYDYYKRLMERPCNFMVNFFTRCYFAVSITALIMALLTGELLYPAFI
PLQWRTSVFKYAVGLLFQFVAVSLQIVQNIANDAYGPVLLCMLSGHVHLLSNRVSRIGHDKPESVKDNYKELSLCIE

DHKLLMRYLRTTKAVEHMVSASYLVQFGGVGINLCIGLVYLLFFADNYFAYVYYTIHITAIMIELFPCCYYGSMLECEF
HDLSYAIFSCNWPMQPRPFRRNIVNFTELTLREVALYAGGMVRINLDSFFATCKTGYSFFTVIQSMK

>Bcuc_XM_011180396.1

MDSSIDTVNTFKRLFFFWRILGFTTNHNKYLIRLYDIFVTIFATFAFPLHLALGVIFADDKEVVFTNLAIGISTFACTAKH
LMLRPQLSKVIAVNRILQKLDERVQSDEDTHYYIKQMREKCIFMMHFFTVVYFSVAVMAVLSALWSGKVLYPAYVV
VDWHGSTWKYLAVMLFQIYGLNMQIVQNLTNDAYGPMILCLLSGHVHLLSRRILRIGHEHETEVERNYAELVHCID
DYKVLMSTTRVVERVISSSYMVQFTAVGINVVVGLIYLLFFADNLFAYCYYVFHILAIMIEIFPCCYYGSMVQAEFHAL
SYAIFRSNWLSQSRTFRRAAVTFTELSLKDVTVTAGGMMKIHLDSFFKTCKMGYSIFTVLQSLK

>Bcuc_XM_011188531.1

MAYEAKPPGVKQLFRTHWTVWKWLGQVIHPQYPKLHIAYTVLLNVGFSIGYPLHLLLGLLNLKSLQEVLLNLTISVPV
AVCTLKYFNIWRNLDKVRHLEQTYNTLYARIDHPEEWLYYRKIIIPYALKVLHLFYFICVGTAITSELTLLIVGFAYEWRL
MYPAYFPFDPYASTGGYVAAHLFQIIGLLVQLAENLVSDTYGGMCLSLLAGHAHLLGQRVARIGYDEQKTQEENNR
ELVDCIVDHNMLFDCHRTLTDIIGLGLFMQIISASLIMGVVIIYLIFFVGNSFEYVYYGLFLFACIMEVFPTCYYATYFEIE
FEKLTYMMFSCNWMDQNRQFKQNLIVCVEQSLNTRYFHVGGMFRINLQIFFATCKGAYSVLAVALRLK

>Bcuc_XM_011179774.1

MSAVQPQQQQQQQHAAASLHSSHAFRYLWLNWRLIGMHPTRRHRLPYYIYSGCINISLGVFLPATMIAKLFFIENL
SQLIGLLYLGVTLTMATAKQWSLWLHRSKLLAVNQYLDKLDARCMPHAVDRQHIRTAIRICHLYYAGYMFVYELCSS
GFAYIGFTLRQLVYDGWFPQFYADEATNLTVTLIYQNFAVMSFFVLQNVNNDMYPQCYLAMMIGHLRALTARISRI
GKDGVLSEDENIAELTNCIEDHKNLLGYFACIRPVISRTIFMQFGITAFVLCLTAVNYVAFERDAAQMLIAATYIFAVLI
EALPCCWYVNSLMEECGELTTALYNCQWYDQNRKFRKMLIIFMQRSQRTMLLMAGDLVPITLQTFLNIIKFSFSMY
TILKG

>Bcuc_XM_011179776.1

NLQAIVNNFTIFQLTILHPGENTSKMSHKPLLTATTLDTKEAFSYIWYCWGFFGMYPDLYERRLNWIYLILLNLYCGVI
YPLLYIGSLLTPMDLNQKLANISVAVPILYTLGKHVIIVVYYLRKDLPKALRQLQALDRLAERRPKDREYMKRMVKNCH
WIFLASSVSFWFALLSYGVLEMFRHKLPFEGWVPFDWQRTEFAYVCACALQLFGLGIETTNAICCDTYAVTYLVLLV
AHLRILNRRIARAGNTGDGSDAENYRELVACVEYHKECISYYNSIRPTLSGTCFIQFLSTGLGLSMPAIAFVGGSFSFSH
VIKFLIIFGAIIIEVAPCCWFMDEVMAEMHKLTNSMFSCRWYDQNLKYRKALIIFMQRSQIAHPVLAGNIIPVSLETFT
NIIKFAFSLFTLLNQLSHN

>Bcuc_XM_011179775.1

MNNKQDAVGRLDSSDALRYVWLFWRITGIHPTAKYRGIYWLYSLLLNISSSVLFIAFYVVTFFISTDLLETLTNLSVMV
PLIYTSTKHLVVFYHIRGKLPQAAFHLQALDRRVELEPAACEHLRRLVQRCHRIFLAALAGIGVCLALYALVGIARHKLP
FEGWLPFDWEHSLNAYILACAYQLFCLSVQSIYALCSDTYSIIYLLLLVAHLRILNARIARIGGACAAQCGEVANYQQL
ADCVRDHWECMKCISPTIAATIFVQFLSTAFALCTAAVAFVNADSSVEQLMKFLPYLLVVLCEIAPCCWLMDEAALE
MLKLTNALFSCCCSDGN

>Bcuc_XM_011189506.1

MGDLPITIVLSNLGVAINCVALPLKAIHIKVNIDRLHDIGLIFKRLDARYQRPEDQLEVREAVKVSTRIYAIFFFLYWFYG
TAMFYLQILLQINLTNDAFPGIYIRALRTHIKLLTDRVSRLGLNPDLSDQENFEELVDCIVSHQELIQISDTVGSILSLTTF

FQFTIYAAILCVCMLNMFVFGDATTKVVTVVYLIPVLWQTIPTCYQASMLESDCAKLPLAIFHCNWLALDKRCYKLIIY
FMQRSQEEISFTAIKLFRINLGTNLSIAKFSFTLYTFINEMGFGETLKERLE

>Bcuc_XM_011179777.1

MSAEEKHQQLIDCIATHQQILQVVDNVRSVFSPIMFIQFSIVAIVHCVSMVNIFIFADNLNMVITTIYYFTVAMEILPT
CYEASMLALESSRLPDSIFHCNWLALDKRGRKLIIVFIHRAQVDVTFVAMQMFEINMQFVATVGFIRKWKVSTVTE
CLSTLQAFINAIAASAKAFAVLMYFKRIKNVEPIMKDLDERYKKPHERQQISDCVASCTRLYASIWFIYYLYGNMSILT
AIVLHKQPFGGWYPFLDVIPNPTVHFYSCFIFETCYMYLLLTAQYLNDLFPTLYMRTIRTHIQLLRERVSQVGADPDM
SDEEKHQQLIDCIDIHQQILKVVNIVGSICSPTIFIQFSVVAIVHCICMVNIFIFADNLNLMITIIYYITVGMEILPTCYEAS
TLEMESSKLPVSIFHSNWLALDMRGRKLIIFFIRRAQVDVSFVAMQMFKINLQTYLVIAKFSFTLYTFVNEMGFGQNI
KDLME

>Bcuc_XM_011186484.1

MPNLIRVGGACIYKSRDSLTYLFKIFTFVGINPSEQQSRKYYWLYYSYSLTVNFICCLFSPLSFHIGYIKLWHVLNNNQL
LAAIQNAVQVTGIPIKILVITWYMKRLRQAFKILDQLDVNYTQHEDLAKIRECVRRCRKIVLIFCLPYYSFELSTIALGLL
QKRAPLAAWVPFLDGQRAAWEYWTIVLWDTFVMFILLSYQLGGDTYPLIFLNIIRTHVQLLVTRVSRLGRDGALSA
DEHYAELLACIRTHVQIVSIANIVAPVISVTLFTQFATTATTLLTWFGNVEYPENIISFAFFSCQLLQIFPCCSSASQLIAD
CERLPDAIFHCNWVDQDRRFRRAILFFLQRTQKPMRFWCLKLFVVKLETSVAIGKFAFSLYTFIQESDVGRKSNN

>Bcuc_XM_011183762.1

MFDLIKGRGRTVFASRDAVIYLFNSFRYIGFNPPATYRVPYFMYSAIITFFAVVFSPVIFNVGWLRDRNKLSVMEILTC
VQASLNVMAVPLKCITLAMAHKRLRGIEPMVTELDERYTTAEDKAKIKQCAVTGNRLVFGFAISYFLYETLTVVSALV
GGHAPLSLWIPNVDWHRSTWEYWLQVSFDSAVLFFLLYHQVLNDSYPAVYIYIIRTQVQLLASRVEKLGYDEQKSV
DENYQELLECIVIHQKILKIVSIVESVVSITVFTQFLVAALILGVTMINIFIFADLTTKIASSTYFFCVLLQTSPTCYHASYLL
ADCDELRIAIFHCNWIAQNKRFNNLLIYFLHRSQDSIPFFALKLVPINLATNLSIAKFSFTLFTFIQEMGLGENLKG

>Bcuc_XM_011190972.1

MFDLVKGRGRNVFASRDAVIYLFNTFRFVGLNPPPQCRFLYYFYSGIITLFVVLLSPIIFNVGWIRDRNVLSVMEILNC
VQAALNVVGVPIKSITLAMSLERLRSVEPLLSKLDARYTEPEDVAKIRSCAIIGNRIVFGYIISYMMYETLTVVSALLGG
HAPLTLWIPYVDWHRSMWEYWLQVTFDGAMLFFLLFHQILNDSYPAVYIYIIRTHVQLLSNRVKRLGTANKSQDET
YHELQDCIVTHQEILRLVRVVEPIISLTLFVQFFIAAAILGTTMINIFIFADFATRIASLTYLFCVLLQTSPTCYYATHLQSD
CQDLTMSIFHSNWLAQGKRFNTLLLYFLHRSQADIPLFALKLIPINLSTSVSIAKFSFTLYTFIQKMGVGKNLK

>Bcuc_XM_011184959.1

MLCKMSKLQPQLTAVMAKDVKTAQRNTNVQRKPLSVLEAKNLVKTLSEETVEEDVVQPVSTQDTTKYLFKAAFLM
GIVMPSRYRALYVLHSFWVNFLTTFYFPIGFTLIFFTLSDEINISNLLTSLQVIFDVYGGSAKFIVMMCMLEKLRATQAI
TQQLDRRCRAADEIAELQKMVRFGQKVVIFYLTIFLCYSGSTFMASFFSGYPPYSLYFPFLKWRRSHSEFIIASFLEFVI
MDLACLQQTVNDGYPVIYINMMLCHMKILQLRVQKLGNNTALTLEEHLSELKLCIKDHQLLIELYDIISPIVSVTLFIQF
TLSAVCIGTTLINIVIFANEFQTQVACCFFILAVLIEIFPACYFSQCLIDESDNLSDVIFHSKWVEQSKEYRKLMIFFLQRS
QRPMFLTAGKLFPVTLSSFVSIAKFSFSLYTFIEKMNLKERLGIE

>Bcuc_XM_011184958.1

MEDAPSSSHQLQADIAAQRRTETTDKPAVRTVQATDYLFRGFRVLGIYMPKHRKWLYALYSLIPNVLVTIWLPLSFV
FSYATMSTEELVPSSLLTSIQVAINVIGCSVKIVVMAFLLPKLRTANVFMDRLDARCRVEAEIAELRKLVKQGNRFVVL
FAMSYWSYASSTFLGSVIFGRPPYALYNPLIDWRKSKLEFIAASIMEFALMDVACFQQVVDDSYAVIYVCVLRTHMN
ILLVRLSRLATDDETNLDANLEELKLCIIDHKNLLGLYDVVAPIISLTIFIQFMITASILSATLINIFIFAEQLSAQIACCFYILA
VVVEIFPLCYYAQCLMDDSERLSQQIFHSNWVAQDVRFRKMLVFFMQRTQRVMELNAGKIFPITLGSFLNIAKFSFS
LYTLIKKMGIRERLGLE

>Bcuc_XM_011185838.1

MADEETQKTGIPTTVNKGYPPYMERRDMFRFMRCNLWFTAMYRLPLERYFPACLQCLAITLDWAYEVFLYLTLLHI
DILFMCTIYLNKDKGDLELIVSCMIQTVIYTWAIVIKVFFKRIKPKRIRELMRYLNEDCRTRSAAGFTYVTIKESTDLANV
WTTVFLICCYAGVTFWLFVPIFNQDRSLPLACWYPIDYKVPVVYEVVYFLQTVGQLQVAGAFGCTSAFYLLVSVLFS
GQFDVLNCSLKNILATTYLSLKNPKSELITAEIIKKNPKDEHFYEAFKIAFRSCIAHHRYILRGLKMLEELYTYLWFLKTIE
VTVLVCLVAFVWVKSTAANSFLRLLSLSQYLLLALWEMFMICYAGEIIFLNSKRCDEGLQRSPWYLHSSEIKQDALFFI
LNAQRPFRLTGGKMYNLNVKKFRSILTTSFSILTILQKMDLRQPQPK

>Bcuc_XM_011185840.1

MSPPSKRNIGLTGSCTNPNGCISNEKRSDLFLYVRWLLFFSAIRPIPFDKHLPRRMHGHSVLVNVIWEIFLYLVVLHIL
VLFIITIYLNYDNGDLEFLISCGIQVLIYLWAILIKVTFRRIYPELVNGIVDFVNEEYVQHSALGFTYVTMKECVDRVNGG
IRIFVPCCFSAVIYRFILPIIYNDRSLPLPCWYPVNYKAPFIFQILYFFQILAQLQMSAAFTVSSVYFISLCFLLSGQFDVLN
CSLKNIVATTYIYMGASKHELIELRDNERIPGEEINEFFVAKELPFDLDCLPHILNPADTARTRSFREAFNYALGSCVKQ
HNFILNALLKLERLYNLLWLFKTLDVTLSICMGTFDVVKSSDEKSFLQLLSLGQYLFLGLWEIFMICYAGEIIYVNSQRC
DEALLRSPWHLHLREVRADFLLFLMNAQRAFKLTGGKFYPLALEKFRGIVSTSFSFYTLLQNLDERN

>Bcuc_XM_011189995.1

MKDKTARDVRELESSTLLYSDEDKPRIADLFVAQVICFKATGQIPFNLGYGLGYVYCFFVITQTLHMAVLFLKTSYEML
LNGKLEEITDALTMTIIFWFSVYAACYWLLRWQRLLVFLRRINHHYWHHSLPGLSFVSSHRTFVLANRMSIVWTVAC
VAGTLLYGFAPLVMGVHVLPLKCWYPFDPLQPYVYELLYVLQLSAQMIMGATFGNGSALFVSLVILMCGQFDVLYC
SLKNLSYYARLRSSFEVEKLRNEQAALPITSDDELNQYMYCQEHLTNLSTLQLLYTQQSAVTLPEALHMAVVQCVQL
HRFILDACKELEELFNPYCLVKSIQVTFQLCLLVFVGVAGERSMVRILNLVQYVTLTFIELLMFTYFGELLRGHSVRCGE
AFWRSQWWTHSVAIRKDIIILLANSKRAVRLTAGKFYAMDVERLRSVVTQAFSFLTLLQKLAAKNQK

>Bcuc_XM_011198388.1

MISLSTQATINNTISTHNSYLSNDSNTHLKHTASKLRTILAPYRVLKEMLRSGEAVHPPHTCLFYFRAYIRLLGLWPAE
RAVENPLYYAFNVLIMLLFGFFTLCLIFFKMILFRLGNADTDIIINEFDALHVKHFNESHDSPRNRRTRQWQRSFFFGE
MCFFSGFYILSLFLFAAMSLQPLLSQQILPFRCKFPFGLDDPDEHPMGFVCVYFFQCFCTLYMLVAIVVMDSLGGNS
FNQTTLNLRILCENMRNLGNGSTSELVVWRKLKETVEFHQQIIKLMNRINQTFYWNYVSQMGASTFMICLTAFEAL
LAQDKPMVALKFQTYMFSAFMQLLYWCWMGNRTYYDSMEVATAAYEVRTWYRHSPLLQRQLIFIIKRAQKPLEF
RAKPLFGFTFASFTSILSTSYSYFTLLRTMSD

>Bcuc_XM_011197519.1

MVFEDVEEIYRRNYNSIKVLFQVSFGLGVNLTAPSKIKDALKLFNVILVVASLLSMYAHWCYLIRHFENIPLLAETVCTA
LQTLISAFKMIYFLFRQHNFYRLLDQALKHVIIREIEIFKHDFPINQQLKREIDEIMNGVWQNARRQILFYFCCCVGIVC
NYFFGAFLVNLYHQLKKTPDYKYVLPFPELYPFWEDKGMTFPYYPIQMFISSSAVYIAGMCAVSFDGVFIVLCQHAV

GLVRVHNLLVLRSTSPLIPVERRVEYLRYTIFTYQRIYTYVQQIQTSFKHISLSQFVLSLIVFGFVLFEMSFGLVSNCESSIII
FIRMIMYISAGGTQIIIYCYNGQALTSVSEEIPMAFYNCNWYEECEKFKQLLRMMIMRTHRYFYLEVSWFTLMNLAT
LIALFRMSGSYFLLLRNLQES

>Bcuc_XM_011188593.1

MVRLISSLVGADVSTVNYRVNIITVILILCIVIYFIFTATTVASVFSEDWTFLLEASCMMGSGLQGCTKLISGIFRTKDVS
SMRLELEEIYRTYESKGQSYCKVLNESCDRVRKVIKMVGYIYASNIAGIILLTTVLMLTSDRKIYIMQFFIPGVDADTSF
GYLLTTAVHMVVFLAGSFGLFGGDLFFLIYLGQPELFRDILILKVEELNEAVAQKDDNIESLLINIIEWHQYYMDFNNR
CNDIFYYIITMQILTSGISIICTMYIILMGDWPGAYLYILVALCGLYLYCIIGTRIQTSSETFFEELYNINWYELDIKKQKM
MILILMKSQNPSEIKIAGVLPLSVETALQITKSMYGIFTMMLQVMAEEL

>Bcuc_XM_011188607.1

MAVKAIRPTETFTKILNFFHLICSLVGADLSNDNYRVNIITVIVILSIIIYFIFTATTVASVFSEDWTYMLEASCMVGSVL
QGCTKLISAFIFKNKICGMRAELERLYAEYEVKGDEYVKTLNKSCERMWQITKVVGQMYLYAAVGIVVTIIYFVIATT
QRVYVMHFFIPGIDVNTQTGYLITLGVHAVVFMSGAFGLFAGDLFFILLFLTQPMLFVDLLVLKVKALNEAAAQKTNA
VQRLLIDIIEWHQYYTDYNKRCNHLFYYIISVQIITSGISIICTLYIILMGDWPGAYMYILIAFSGLYLYCILGTKIQDCNSA
FCNELCNINFYDLEVKSQEMIVLIIMKAQNPVEIKVGGFLPLSVQTALKITKTIYGIFTMMIRFLEEEQ

>Bcuc_XM_011179576.1

MNAIERNTNFIRFTAGPVRYFKFIGICLQPPEMRSYKCTRILIVLTILLMFLHQIGYILTPGRTFAEQSTAAGLLNYTTVS
GGKILFLMYNRHLLLNSHCQLAALYPSASLERRYKLEHYLRIYARVQTLLYNFFKYILIIYLLYPLVQSCYDLWSNGVYSY
LMPTLFWYPVPLEQSLLVYIVYFLFACFCSFCAGLIILSADLCLFSSVSQLMLHLDLLAQRISELQPAEPESLSALKAIIEY
HQKILT

>Bcuc_XM_011184736.1

MALRQEKYGTAKLDDLCDILHPVQRYLRLNYLDFRRVNGRFAIPNSKLLNICLILAVFDCIGNCIKCVKAINAGEITKA
QEIFAVFGMGFVMTMRGLMLALNRVQLSNFYNKIDCIFPRSAHLQQHMAVEKVHSYIKRRFYIMHTLMTVTVAAF
LTTPGVKFMVFHDFDSDDSVADEYHVNPSWLPFGLKDKVSTYPYVYIYESVLAAAAVNMIITWDELFVVLISQLCMY
YEYLGRLLEEMNVQDALDPTKLDAFYEQLHEYIYMHQYLNKLAVEFNDLFNFSILFSDAGIATSICFNIVLITDATDYLQ
IVTYTSPLFVEVWLIYDAAKWGTMLETVTGRINEILYEQKWYESSVRFGKYTMMWMQSTNEPFRLTAFNMFYVN
MKHFQDMMMLAYQMLTFLKSKS

>Bcuc_XM_011192811.1

MEELKALFPSIAKQKRLAEINESKEGIIGSGGIYRLEYYEEKSRTIMQFITRYFMFAYVAYNSIPVMQLCFAVITQQEHIT
YRAQANAWYPWHNHNDHSSFMGFMLSYLTQAIVEYTSIAFVMSGEFIFCFFTTQMLMHYNYLCSALSALDASAP
DAVRQLKALISYHTHLLRLSKLINSIFNLTFALDLIITTFAISLMGLAIVLVNFADALMFSAGFSFFLLLGYLFCNNGDEIL
RETMQINSAIFYSNWYEGSPEYRRLIIFFIMRTKTPCQYQAYGYTPLSMETYMRILKLSYQMFTSVRAIE

>Bcuc_XM_011192799.1

MSETYTLNDCLKYPYFTLDLASTEPFTWSGAPTHSYRKLWLRRALFIFGALNLVYQNVGMLIYLFMPHESSAETTIAQ
ITETGGIMGLTMVGTSNMLVMFWYADRIAILLEKFQQLFPTERVQRQVKLEFPHRIEYFAVKSNKLMKLATTLYMY
AFAYYNSLPIVEYLYEWSTPGIVWKYRYQSNTWYPWQNERNSKSFLSFAVAYICQVQSSLTGVAFIMAAEFMLCFFT
TQLQIHFDYLANALETIDAAAPNANEDLKYLINYHSQLLSYSKETNAIFNVSFMVNLCTSSIAICLMGFSMVMISLAH

AFKYSIGLTAFIVFTFFICYTGKELTETSDKLLHAAFYGNWYDGNLAYRKMILFFIMRCRIPTELRAYKFTVSMPTFTAIL
RSSYSLFTFFQAMGK

>Bcuc_XM_011182964.1

MDFVHFFWFPNALYRVVGYDFQQLARAHWRQVIMKAFLIFTTISGICTRIYMLFQLRELILNGDILNSIRLGVYISYAI
DSNVKFFVFLLNAQRLRVIYQTLYNEYPVTPIERKLYQVDKYSFKRAHLMMVVYLSVTNSILLGPMLQSIFMYFVNLF
HYGYAVAEFPYLHPTPVLYNFNYCTPHYYILIYISEYLNGHFCTTTNLGADLYVCTFAGQFCMQLEYLGNSLETYEPRV
ENSKTDCEFLMEWIRKHQLMLDLCCELNEVFGTTLLFKLISNCTVFCAIVVQLKLEGFGIGFFNFLSFFFVTVAQFFMV
CQYGQKLITISEDLSLCAYKNRWYNGSQTYKILLFNIIARAQKPVKLTARGFQPISLATFQIVMTMTYRVFAVLQRALD

>Bcuc_XM_011192722.1

MLPPFKSREHAPTVQDFVYVPLFQIRFMGVKLFKWTPEERTSKLQITLMGTFCVFATFNFVSMMLFVIYDELPTSLDI
TEFILFWGFALNAMMKGGTMVFFRHEIESVLKGLIARHPKTEAERVAFQLVPYYKTINASNKYLSIWHLSITSIFALHP
MVASILGYIWREDKNDAYVYTLPFMMGYYYDTNHPFPYAISYFIQCCGAFYMSLLFLSGDLLLISMVQLVNMHLEYL
IYRIESFQPTGMDADMKVLGPLLEYHNEILDYAERIDGTFSLATLLNYGGSCLVLCLIGLQIVLGSEALSVIKFIGFLVSTI
VQVFFVSYFGNNLKELSTGISDAFYNHPWYDGNYKYIRMLVLPIARAQRYAHLTAFKFFEISMDSFKSLCTTSYQFFTL
LRTSIEEEGFQ

>Bcuc_XM_011181747.1

MSSEARTFSEFIRIPIRFYQTIGEDLYEHRSPYRTRRLLLKSLLYIGFINFNLLVVGEIIYFIKALNSFATVLEATGVAPCIGF
SFVADFKQMALTAHRQTLREHLDQMEELFPKTTAQQVEYKLPQRERVMRRVMAVFTLLCLAYTSTFSLYPALKASV
QYWLLSAPVYERNFGFAIWYPYNATQKTWVYWLTYMGQVHGAYLAGVAFLSADLILVASVTQLCMHFDYISCCLE
DFAGATPKRSAQEDLQYLQALVVKHAKCLELSEHVNSIFSFSLLLNFLTASLTICFIGFQVTASSTEDIVKYIIFLTASLVQ
VFVVCYYGDELMTASLRVGDAAYNQNWFDCDRRYKRLLTIMIMRSQKPACIRAPTFPPISFRTYMKVISMSYQFFA
LLRTTYSGKGN

>Bcuc_XM_011194223.1

MSNQPIQFERFTILANAYYTSIGLDAYEKTGQRTSNIRRRLLSIFFIFTIANMNITLLSELLYILMAFVSNNNFVEATMLL
SFVGFVIVGDVKIYSIWRQRERISAMIQALHALYPQTLAEQAKYAVERELQRYKRFAYAFVLLHELLVWSYNLFPLLNY
FIYEVWLTWRVVGKTLPYNCWTPFDWHNSDWRYYTMYLTQIAAGQACLSGQLANDLLLSAVAVQLIMHYRQLAR
RIEAHVADSRDDSASKRRGESANECAAHDLHFLRTIIAYHQQILNISQVLNDVFGISLFINFTSTALIICFVLFQITIGANI
DSIIMLAFFLFCSLVQIFLICYYAQQLLEASEYISFAVYNHNWFVADLRYRKMLIFIMARAQKPSKLQATSLVTVSMST
MTDLLQLSYKGFAVIRTMYAREPKSVSN

>Bcuc_XM_011194222.1

MAPLRVCPSIMSKIIRFEAFLRIPNFFYRSVGVDLWNTDGGPLQDAVFYFGLLNVNVWLLSELVFAVLMVSKNFIQA
TMTLSYAGFVLVGSIKMYFMWRKKAEMKRFLQLMNAIFPRTEQQQKIMHLRRHLRQSTIVMSCFAMMFMVLIW
TYNLYPYMQRQIYDRLLDVRSINKTLPYESYIPWNWHEHWTFYLYYTLQSIAGYHAASAQIASDLVLCAMATQMIM
HYEYVAQKITEYQPQVGADSGSKNEAYCRDMKFLCDIIAYHANVLSLSDIMNEVLGVPLLVNFMTSSFVLCFVGFQ
MTMDAEPDYMVKLFLFLFSSLVQICLICQYGQLLIDSSSNIAHAVYNHDWVHSHVHYQRMLVLVAVRAQKPAMLQ
ATSFVRISRGTITDIMQISYKFFTLIRTMYSN

>CcapOrCo

MQPSKYVGLVADLMPNIRLMKYSGLFMHNFTGGSGLFKKIYSSMHLVLVLVQFLLILVNLALNAEEVNELSGNTITV
LFFTHCITKFIYLAVTQKQFYRTLNIWNQVNSHPLFAESDARYHSIALAKMRKLFTLVMLTTVVSAVAWTTITFFGES
VKFAFDKDTNSSITVEIPRLPIKSFYPWNAGSGMFYIISFAFQCYYLLFSMVHSNLCDVLFCSWLIFACEQLQHLKGIM
KPLMELSASLDTYRPNSAALFRSLSANSKSELINNEEKEPTDLDVSGIYSSKADWGAQFRAPSTLQTFNGMNGTNPN
GLTRKQEMMVRSAIKYWVERHKHVVRLVAAIGDTYGGALLLHMLTSTIMLTLLAYQATKITGVNVYAFTTVGYLCY
ALAQVFHFCIFGNRLIEESSSVMEAAYSCHWYDGSEEAKTFVQIVCQQCQKAMSISGAKFFTVSLDLFASVLGAVVT
YFMVLVQLK

>CcapOr1

MDKLEALSSRIFPSDPSIGKIGSIEYNVWLAQLFGVPVLGLKKETPRMRIALAVYGVVATLVVTFLYTGFEIYDMIFCW
PNLDKLTQNICLSLTHVAGALKVINIIYRLKEVAGVVRKIEYAARYYVISKNQLKAFYRGEFENKIPLTIYASLVGFTGILG
IAYLLHNPTGVAGEIFPYRVKLPHWMPFGLQLAYMGFSVLVFALQIVAIDYLNVTMINQIRFQLKILNLAFEELKFVSG
QAAHELSLDRRLRTIVDHHNLLRNLRNEVEEIFRLPVLVQFFTSLIIFAMTGFQAIVKSENSNGASLIYCYCGCIFCELFV
YCWFGNEVSEQSKTLTTSGYNCHWYQFGPRYKKSLLIFMFNSQKPIVFTAGGFMALSLPSFTGILSKSYTVIALLRQF
YGR

>CcapOr2

MHRLLCKFEQVAMRVFPSDVRRGQIGSIEFNIWLSQMTGVPLPPSFVPLRRRFVSNCLLLLCGSFLCFVHFMYTIFEF
YDLYIVWPDFYAFTQNFCLTMSHFAGALKIINLLCRLKTVQRAILKLKQATKTYIRTDAQRQIFDRAEVENKLFLVIYAT
VIGFTGFTAMILVFINPDMAGKIFPYRVALPAWLPLPVRVAYIGTTDFMFAVQIVTVDYLNIGMMNLLRCHLNVMK
SSFDELNFNVKCMKSDIKRIRDPNERLADIVRHHCVLKSVRDDVEQIFRLPVLLQFFTSLVISAVTGFQATIYSSNFKSE
LIIYFYCFCIFTQLFGYCWFGNEVNEQNKTLAARGYSSSWYYFDNRFRKSLAIFLVNAQQPFDFTGGGFVALSLPSFT
GIMSKAYSFIAVLRQMYDR

>CcapOr3

MRIIVLKDITPVVDAFACIGFNIDLNKAKGSFSQPVRYFVLLIGVIAWTAALAAYTMQYLTDVDKMVAAMTINVQLF
LTTSKNLIFLARRKRFLHLNEALERLALNGNESERILWNTTNRFVLPITRAYRISSELTVSFCVLLPILKLLYYYIFHSEVVL
TLPLPGIFPYNYTLPFYFILTTILTILLVYLCAYTIVAIDGLFGWFIYNISAHLQIMSLRLEQILQLPIEDPRFHRHFVDLVNY
HKEIIRLSLELDAVYAPIIFLEVTSSSLPICFLAYQLSYLSDPANVPFMCLLMSSIVIQLMIYCFGGEKVQSECDQLCENIY
LLIPWQNLPQKHCRLLLNPLIRSQRVLVLTGYFFTANRSLLVWIFRTAGSFTAMLFALKEKDV

>CcapOr4

MSKYLRIQYFSFRMIGINLWAKRDQRIMSAPYRYCIWSMATAIITILMGFFIYSNEQDQAITVLTVFLQGVLSVFKSG
MFVLKSGRFIELIRNLDMLAEKEHSAWRLENDWQQRIVSVYYFCCTSTGILYCTVPALVLLYTRYFREQTVFILPFEAS
FPYDTNQPLFYILSYIWCISFIIYAIHAIVAMDSLFCWFIFNISAHFRILQQKLTDVSVSPNASTDHAIFQQDITQTLFYH
QRIIELSAEFDELYAPIVFVEISVSYLKLCFSAYNLINLDDISSLPVIAVGLVTITFQLCIYCFSGEKIKNASEQFANHIYLSFP
WERVPPSLRRLLFVPIMRAQRPMHLTGFLFIVDHSLLVWIFKTTGSIIGFLSATKKENTNI

>CcapOr5

MFHSDLVINGYFQLQKHTFQRLGIDMTTRNASITHIYFLALQIVALVTITMPMVIYSCQHLQEIAEVTNAMAPFMQ
ATITLWKIWRVIYRRKMMAELVEDIYSVSTKATKQELTHLRRENNRERIMNTFYYYSVFNTGVLALIAPVLVSFIQYLR
LGEFSYIVVLKATYPIELARPLNYVLIWLWSAIAIYGVIYGSVSVDSLYSWYIHNLVGNFKILQTKFVTAELIAEVSERRA

SIYYCLGYHQRLITMSEQLNIIYQPIVLVQFSLNALQICFLAYQIGSGDVAVVDLPFLFLFMTSVGIQLMIYCYGGQYLQ
NESENVAKFISQTINSTIWPIDLRKVLLFSMARAQRPCKLTGIFFDVDLRLFLWVWRTAGSYVTLLRSVDQQSS

>CcapOr6

MPEDLFRIQRNCLRLMGHQDIYDDDGDDNNSSNNNDTEFSSVERKSLRQRLCFRHLQTMKYVLLLLLMMSAQLP
MMDYIIYHIDDLELATACLSIVFTNILTVIKTSTFLAYKREFKSLMTEFELMYDELRGAGAKPLLVTVNVGAKRFVKLYF
YSVSCTGLYFTIKPLIGMFWAKFQEKPLLLELPMPMRFPFDFESTPGYQIAYVYTILITIVVVMHATSVDGLFVSFTTNL
RGHFQALQYFIETNTFNKSEARLQKELRFYVEYHVRLLGLAQSVQRIFKPIIFGQFLMTSLQVCVIIYQLVMNMGVIM
EMVIYCTFLSSILLQLLIYCYGAEFLKIESSAVGTAVQMSQWYNLPPRHRHVLRLMMVRSQREIIISAGFYEASLANF
MSILKAAMSYITFIQSIE

>CcapOr7

MLFNPKPLKDPVNFKFPLQCIWLKLNGSWPVNSKTSSSFKNYCRFLYSLWAWYVVAMVGITIGFQSAFLAKSFGDI
MVTSENGCTTFMGVLNFVRLLHLRLHQRDFHQLIAQFVKDIWITKSTHPAVEQSCARTMRVFQVISVLQSCLITMY
CILPLVELYMLNANLDQESLAHIDKPFPYKMLFPYDANHGWRYALTYLFTAWAGVCVVTTLFAEDSLFGFFVTYTCG
QFRILHTQIDNIIPAAYAATRAGRGTEADYQRECVRRLDKIAGKHSILFNFVSRMEEFFSPIFLVNFLISTVLICMVGFQ
LVTGNNMFIGDYVKFLVYILSSLSQLFVLCWNGDKIIQNSLEMANHLYACNWESDIVLTNTHTNNNQQMKNPTRII
YYSTGAVFRKNLQFMIMRSQRQTCITAMKFSILSLSSFSGLMSSSMSYFALLQSFYEDEEN

>CcapOr8

MRYLPSSYHKPLLPNGRHPPIDWQLYGFVCSNCWPLARDITKTRRIIDMVITAMQFMSESMVLSGETIAMRNNLD
DISFVCMVLAPYLILIELMLRAYNIIYKRNSFRTHIEEFYKKIYVQRTWNPELFEQIRRQHLPTKYSTFTYIITLVTYVYVPI
SGLVKNERLVPFPIQFSFDFTVPWPRYVVFLIMSIWTGFAVVGPLVSEANMLAMQILHLNGRYSLLLRDLRKIAREAI
EEHEKCKGRDKISVTQRFRYSLFDIIRRNVELNEFAKSLQDQYSFRVFVMMALSATLLCVLGFLTATLGLTAENIRFVS
WIIGKVVELLIFGRLGTTLSTTTNDLSTSYYCCDWEEIIFHSADAEENRKTMKLIALAIHLNSNPFQLTGLNFFVVNYET
VVSILRGAGSYFTVIYAYR

>CcapOr9

MLYRPRLPNGKLIPLSWPLAAYRLLNNICWPLRDNANRLERLFDRFCWALGFFIFIQHNDAELRYILSNNNNLDQML
ICGPTYLILVEAHLRAFQLGLKKNNFKNFLKRYYAEIYIDKPTHPKLYANIQKRLRPIWFYSFLYYSTLFSYVITPLTNYLK
NVKAPLFKMYYPFDITPNPIYVAVVLSNIWVGFTVISLVAGEDNILSEVMLHLNGRFLLLQQKLRQNADRLLHNTDG
RYIADALQDQIIEAIEENVRLYEFAKGFEREFSFRIFVNLSFSAGLLCVLCFKVYTNPMASYGFMFWICAKIMEMILVG
QLGSTMIYTTNEVSSTFYECNWELVLMKSTDTKANVRLLKTLALAISTSGKPFVLTGFNYFSVSLTAVLKILQGAGSYF
TFLTSMR

>CcapOr10

MSIKFLTQSYPTEKSLFLIPKFVLRIVGFYPEQEKSTIRRNAWTMFNLIMLIYGSYAEFMYGVHYLSIDAVRALDALCPV
ASSIMSVVKLSFLWWHRVELERLIRRVSVLTAEQDSRLKNNYKRRYFTIATRFSAALLCLGTCTSTLYTIRAALANYFSY
VRGENVPYETPFKMIFPQTLLSKWIFPVTFTFSHWHGYITVAGFTGTDGLFLCFCMYFGTLLKALQIDLKDLLKDMD
CGQHEGLSERDIEECMKKTVMRHNEIIDLIGDFSAVMSSITLTQFVLSSVIIGTSVVDMLLFSDYGILLYFVHALAVTTE
LFLYGIGGTTVIECSSQLATAVYDSNWYSHNVEVKKMVLFMILRTQRSLVIKVPFFAPSLPALTSILRFTGSLIALVKSV
V

>CcapOr11

MKFKFLSRTFPLRDYYFYVPQLCLGSMGFWPMDTCRQQAANVGAWMNLIILAIGVFTEIHAGCTVLRTDLELALDT
LCPAGTSAVTLLKMTLIYYYRQDLAWVLERMRSLVYERDGERIIRAHAVMAARLNFIPFVMGFITCTSYNLKPLLITLIL
YMQGQQPMWKLPFNMTMPAFLLRAPYFPFTYIFTAYTGYITIFMYGGCDAFYFEFCSNAAALLKLLQEDLKSIVSFE
EQLVFTAQESTLLEWRLVRFIMRHNDIIELTRFFCKRYTIITLAHFVSAGLVIGASIFDLMTFTGFGIVIYIAYTIAVLGQL
FIYCYGGSLVAESSVQLATVAFGCDWYACNPKLRRYVLMIIIRSQRAISMSVPFFSPSLITFTSILQTSGSIIALASSFK

>CcapOr12

MLQPLLGSQVPIEQSFFRIPRISARIAGFWPQPAVRPRTWLTVLRFCVNTFAVAVGGFGEVTYGFFYLYDLFSALEAF
CPGVTKVISLLKMTIFFGRHERWQRVIHGLHTLLLLDTSAGKRRIMEPLASFASVLSFVLLASGSLTNTFFNVLPLLKM
AYFKWRALDMQLLLPFNVILPEVLVNLPYYPATYLVLTLSGAMTVFTFSAVDGFFLCACVYATALFRILQHDIRNAFA
ELQEQESSSFEQNMRIQHRLSVLVERHNKIIDLCSDFAAEFSLIILMHFLSAALVLCFSILDLMLNSASIGVLIYIFYSIAAL
TQLVLYCIGGTYVSESSLSIAEVIYDVDWYKCDVRTRRMLLLMMCRAQRAKTIAVPFFTPSLPAFRSIVSTAGSYITLLK
TFI

>CcapOr13

MFDDLQLIQMSVRILRFWSLIYEHTWRRYACLSMTTFLVFTQFYYMFRTSEGIDSIIRNSYMLVLWFNTILRAYLLLY
DREKYEELLRDLENFYYDLKKSKDFYIQDLLNEVNSTGKYMARGNLFLGLLTCFGFAFYPLFATERVLPFGSMIPGVDE
YKSPFYEFWYIYQMIVTPMGCCMYIPYTSLIVAFIMFGIVMCKALQFRLKTLHRCRHVDGLIHKNVKECIRYQLSIIDYI
ARVNGFTTYIFLLEFLAFGTLLCALLFLLIIVDSSAQAIIVCAYIAMIFAQILSLYWYANELREQNLAIAAAAYDTEWFTFP
IPVQKYILLMILRAQKPPAIMVGNTHPITLELFQSLLNASYTYFTLLKRVYI

>CcapOr14

MPITRIEDNPLLAINVRLWKFLSVLFARNWLRCAAFVAPVCLMNAMQFVYLYQQWGDLATFILNTFFATSIFNALLR
TCLVIKNRDKFEALIQELVTMYDDIEATGNDYAKRELAAATKAARKISIFNLSASFCDIIAATLFPLFQENRIHPFGVALP
GIDVTRSPLYEIVYISQLPCPFTLTSMYMPYVSLFASFAMFGKVALKVLQDNLRNLCDNMQHKSEMELFNLLRSNISY
HARISKYVNDSNELVTYMVLIEFMLFSCVICSLLFCINITNSTAEKISIVMYIGTMLYVLFTYYWQANGILEQSLLVSDA
AYEMQWYKCSQRFKRTLLIFIGRTQKPLQIRVGQMSPMTMEVFQSLLNSSYSYFTLLHNLYND

>CcapOr15

MKKLNFVTKLFGWNIKLWQRERFLDEHKRWIAYIALFVPAIMIPPIFCNLYLGHDDLGEAIYDFFTAMIDITGLIRSIVI
LRKQRKFLNLFGVVESWYEDLKQPNEHKALETLNQIVAKVQLYSKCCLYSLLMVDVTYAFEPIITHYGKLVVELQLPSI
DLHQSPVYEMVYLIQALWLVPLTSVNYVSYSNSLLIFTIFGVFATRHLQQKLMEISQMEDDEALANLKQCVVYHSKII
KFGENLEELYSLMSLLDISLYCISVCLMLVYLTMDFTWPLLFKGVIVILFLTTLIFLTYHVADVLTHESMNIAELAYNTN
WMDRDKEFRSCIQVIIVRSQRPLMLTAGGFQPMNMKTFLAIMRASYSFFSVLRSTV

>CcapOr16

MATERKSMPNVERLFNWNLEAWTRLGYLDKRKRLLACTIISAPIVVSMFVMPTFFMGVNTFEQHIYNFYMIIVTSS
SVARAILIIIKQRKILDLLNDMENWFVEVQEQNDNNALETLNKLTQKVRRYSKYTLWWVIIFGAFLSFQPICTGYGKF
VYDTQIPGIDLHQSPLYEIMYGFQSLWVIPMACVSSISYADTLLIFISFGIFATKQLQRKLKDISQMDEQQGLENIKKC
VQYHWKIIKFGEDLENAYSLMCLLDFSLYCVTLCMLLFYSVMDFTWALMFQAVVVELILTLLIFLTTFLADIFTQESLN
VAQTAYDMNWLQRDKAFRVAVLLIILRSQRPLILTAGGIQPLNLETFLAIMRSSYSFFSVLRGVM

>CcapOr17

MLYSEEIYNWNLVFMRSIGYLGNRRRAYFLLSLPVLICFGAIYGTYKMWSDFDKVIINLFKTSGLLTVTLRSFVIIQKEK
KLYDFFDYISQLYRELQAEGDETTLKRMHEFARKTKRYTKGLFILMIACTFYISLIQVMSTFGIGLKKFLIEMELPFISVN
ENPYWDIFSTLQAVWLAPSILLSYISYLCIIFTTISFGILLMKDLQFKLGNMNEKNDLEAYEYIKNCVKQHVMIIKYHRQ
MEVLFSLGSCAEVCNFCIIPCVIIVYSTMDYDLAFLMTDIQLAVIAVSSTFFNFWLANNFCVESLNIAYAAYNSNWIDR
NKEFRKYIVLIMTMSQKPLQLTAAGLKPINMEFFLAILRAAYSLFTVLQ

>CcapOr18

MVIEKPTESNTLIKHAYQNLPLYSANVKLLIKWGYIGTPSRLQRFLLGLLPVLTLIGQVINIFKSPDADMGETGMNFFL
LAIMTNSILKHFTITRKDEYFQRFLQSMQQWCNAMELYEDQRIPGLILDITRRSQKLSRITFYGSVVGTVCGVAYPFT
FEHRKFIFDVQYPLFDIKRTPFYEINFLLQAFVLVPSFLCVYMTFTNLLFTFLMFGEVTLLDLRLKLQNISKDDQTKMLK
DFKDCIAYHNQIIDFRDDLENLVSMALFFEVALFGLMFCMLLFFISLVHDYQLIFTAVTFIAVTLYMIAVSFYFASKFTS
ESLEIANAAYDTPWYDGNLEMRKCVLTMIARSQRPLLITAGGIYPMTMENFQAILRVSYSYFSMLQGLN

>CcapOr19

MITPRKASSIPIIKYTRITMEEPKKIVNCFYERQFKFFKFLGLFGLPPNYSRFCQILFKLYFWHVTVVWMLLFDISMWV
KVIGNITDLNEIVNVFYICSMAIAVMAKFVHIRKKNSRYVAFFARMHNDDLLPANPSELKKFIKSVHLSCAVRNCYM
GLSLTSLALVFVPKLISDPGELPLSIYIPLNVEHTLCFLVAYIFQFVGLSLCCFLNIAFDSLSASFFIYLKGQLDILSNRLENI
GKFQNITQDVITLQLKECIRYYAKLRYITDIMEDLLCIPMSVQVISSVLVLVANFYAMTFLTDPSDYGTFMKFLVYQLC
MLSQIFMLCYFANEVSLRSAQLSYALYSSEWTHCNQINRRLMMLMMAQFDVPIRIKTINRCYSFNLPAFTSIINSSYS
YYALLKNMKD

>CcapOr20

MNNSRLLITMLFILGLWSTTRIPDSWFKPYYRCYKSFVNSTLVYSFILLQFLEFILNENMENADEDLKLLITEICYLAKSL
NLFYHLELAVELLHEWEISDSFNLRRSAEREMWLQGERAFRKVIYIYIFFCLLTVFLAIGSTTLSGTPTLLYQFWLPAKW
REYNIWPAFFYESLSLNFNCMCNVVLDSFQCYLLYHLSLYTRLIGMRMERLGYEKTNVETTSSLENIIDMHQRLKAM
TRCCEKILSVPLLTQITLSAFTICFSIYSLRSLSFIEHPMSLLSAVMYLLCMSIQIFMPCYFANNLTAESQNLSSHLYNCN
WVDLSVYNRRQIFLYMEYLKLPLFVYAGNYFKVGLLVFTKIMNNAYSLVALLNNINNDDQ

>CcapOr21

MTFDNIANSRFLTRALVLLGLWPVITVHGSWLQRNYCFYQLFLQITFTFSFTFLMLLEVICSESLDHATEVLKFLLTEM
ALVFKILNTWYYARKAAAFLHEWETGEMFVLRSNAEKNMWAKKQSTFRKIMLGYIYWSISSAVCALLSCLFINDQA
LPFPYWTPNHWLEDYYWLMYFYELLTMPFTCLCNIEIDVFQCYLLLHLALCLRVVGMRLERLANAGDENAITREFLK
TIKMHRRINDMARNCEQIISLPVTIQIMLSALIICFIIYRMQSVHFSDNPTEYLAMFQYVVAMSMQIFLPCYYANELT
VQSQNLSKSLYNADWTGMSAYNRRLMLLYMQYLKLPLVLYAGSFFQIGLPIFSKTMNNAYSLLALLLNVSDDKDKK

>CcapOr22

MDLQEYDNNSGGRRIIQVMKLLGLWYYEGSAKMPYLLYSCLLHFTISIPFTIFMAMDVVHATDLEKFTNIMYLTLTEL
GMVAKLFNVWFYAKLLVDFFETLSGDKYFELREKDERLKWQHAQRTYARIVLFYVFIGLGAMFTGFVGVLFSAKYEL
PFPYAPPFNWHTPHGYWCAYLYELLAMLITFFANYGFDMIQCYMLLQLSLCFKLICGRLECMGELRSGTAVSRGFSE
QQLYRQFVDIVKLHARIKNLSRLCQTYISFPFLIQIMCSSFVLCFSAYRLQKLSILSDPMQFLTFVQVNLIMILEIFLPCYY
GNEVIAQSSALNNATYNSEWFRCSPGLRKYLVIYMAMLQRPLRVRAADFFDISLEIFTNTMKNTYSLMALLLNMN

>CcapOr23

MEYDNINGARRVIRVLQYIGLWRFDTPWQPLYATYSYILHITCTFAYTIMMWLDVVQASDLEKFTYIMYMSLTELAL
LTKVANVWRYSKLFVNFFHTLADDDVFRLRNAVERTLWQTVHRYYGWIALMYFTMSITLVTSAFVGVLFSAEYELP
FPYAPPFEWRNERGYWYAYFYELIAMPVTCFSNCALDMIQCYMLLHLSLCYKMIGLRLEAMGKWQEQLRCSSCDF
SELKFLDEFVDIAKLHGRTKKLTEDCETFVSFPFLIQIMCSSFVLCFSAYRLQKVSIFENPSQFCTLILAVIVMILQIFVPCY
CGNEIILSSGALNNAIYHAEWLQCSPKVRKYLIIYMEMLQVSIKVRAGSFFEIGLPVFVKTMNNTYSLMALLLNMNK

>CcapOr24

MATAIELQERIGAARVLMRMLQYLGLWPISQTGLQRTRHTQCRARLAHYYRYLLHLPLTFTYNTFMWIEALTRWER
ADHILYISITEVGMMALTLNFWRMERRAWQFMQEISHDSGLTLCNEVERIWWRKQQRTFTLIVICYIGGGAGVLFT
AFGATLLMSGYELPYDYWLPFEWHNARNYWYAYGYELVAMSLTCIANVTMDMMMCYYLFHVALLYKLIGMRLIA
LQYLSEPFAVQKLRNIIELHKKVKRLTVQCESLVSLPILVQILLSAFILCLSAYRLQNMQINENPGQFLAMLQFASVLTL
QIFLPCYFANEITINSDALMTCVYSSNWEGFSPATRKQMYLYMELLKKPICIKAGNFFMVGLPVFTKTMNNAYSLLA
LLLNMSK

>CcapOr25

MTKNSRSRILSPNSRISSGRIIVYVLQAIGLWQWAEDRRQYERNRNLQRLQRLYGLLLHLPLTFTFILLMLTAALLAHD
LEETSGVLYMLLTELALVVKILNIWRQGSSAWLYMDELAHEVLYSLRQESERLQWQSELRSFDIIAYSYTLTSIGVVVF
ACVGVLLTATDIYVLPFDYYVPFEWRHPRNYWYAWSYCSVSMMMTCISNVMLDMIFCYFMFHLSLLYKLIGWRLA
ALRRSRADNGVGGRGDEDGVHREMREIFQLHMNVKRLTTQCESLVSLPVLAQIILSAFILCFSGYRLQRMHIMENIG
IFLSTIQFVSVMTLQIFLPCYYGNAVTANSNALTNDIFNSDWTGFDMRSRKFMIFYMELLKRPATLKAGGFFQVGLPI
FAKTMNNAYSFFALLLNMNN

>CcapOr26

MSVKVNSWEAFKYHWRVWELSGFLGPQKGSFWHVPYKLYTIVITLLFPIYYPICFTVESILTDNLNDFCEVIYIAMAV
VTLNIKFFTLFMVRNQLLQLQPILKRLDGYAQTDEEIHVLLEGSKSAKKCFLTILKLFYSAFVTSQLMVIFSSETRLMYP
AWYPFDYKASRINFWIAYGYQSVGFLVQCTQACSVDTYPQAYMRILIAHMQALSLRIERIGLNANDNTSTSLKYLTK
EEMKRNYYELVSCIKDHKTIIELFSTIQKPISGTSMAQFVCTGVAQCTIGVYMLYVGFNLSIMLNMAVFFISVTMETLI
LCYYGDLFCQECEKLSKAIYNCNWTIQSSEFKRALCIFLLRSQRVNVLMAGNWIPVKLPTFVMVVKSSYSIFTLLSNFK

>CcapOr27

MQIPPPTPSSSLAATVTTTTVDSSHFFRMPVAISWLCTPAALFNSTNLNNILQMLPMPAAALACSVKYISYYRRLDLV
RQVEQIFNALHERVQLAEDRDFYAGIQRRANLIMKTLRAVSFFFFLITVMSFVSSIEERSLAFIVELPFNWRASTKAYM
CAVLLELLLLTCDLLQSLVNDSFPPTALCVLSNYTRRLGERLARIGYGGSKDVHGSIAELRRCIVDHQHLYSSLNAIIEDII
SVPVFVRYAVTAFQDCFTLVTFIFYTDTTSDKVLYFTYLLALQLQIFPTCGSDCAQSSADLLQRVYASNWMEQTRAYH
RLMRVFSPRSMKSTTTYAVGSIPIHLGIFVRTLQGAYSCYKFVNGMRKL

>CcapOr28

MSSPLISSSSSSPSPSPSPSPSPSPLPFPLPQRLAATAVDTRSFFKLHWACFKVLGVVAPTADVFYLVYSVLLHLLVNLC
YPLHLALMLFRSPNSSANIQNLAVCVTCMACSVKFVIYTMKMWRIRELESIVAALDARACSPRERGYFLKLRKDMRR
ITIGFLSIYAFVGVTAELMFIFCNEHNLLYPAWFPFDWRASKLKFYAAHFYQIVGISYLLLQNFVNDCFPTMALALLSA
HIKLLGIRVSQIGHEAKSLDANELELLRCIKDQEHFMLNTIQNIISLPMFLQFTVTAINICLAMAALFFFVDAPFDRLYYL
AYFLSMPLEIFPTCYYGTDFQLLFETLHIEMYASNWVEQTQKFRKHMILFNERSLKKEVAMAGGMIRIHLDTFVSTC
KGAYSLLAVIMKMNE

>CcapOr29

MQQPIDSSVFFKIHWLGFRICGGDSSVRKYRLLDYIYAFAISTLVTICYPLHLALALFRNNSLASDIKNLAVCVTCVACS
LKFVIYTRKLRLVHKIEQTFAELDARVCSEVERKHFDRMRSSVKNIFYLFVCAYTAVGVTAELAFLMSEERGLLYPAWF
PFDWRTSARNYSVANVYQIVGISYQIFQNFIDDSFPPITCCLLSGHIKLLGIRVSRIGYECADVLENERELVRCIKDQKN
LYRLFDLLQAVMSLPMLIQFTVTAFNICVAMVVLLFYVDTPFERAYYFIYFISMPLEIFPICYYGSSLQLLFGQLQYEVFR
CNWMDQTPRFKKHMILFTERALKVIIALAGGMIKIHLDTFFATVKGAYSLFAVIMKVR

>CcapOr30

MLKNVFIFKPAGNAAVDSVACFDIFWMCWKLNGIAVNSNKWYITLYDIAVNIFITIFYPIHLTVGLFMVPTLADVFK
NLAINITDVACSTKHYLFRYKLPKIRELQRLLKQLDERVLAPNEREYFDKKIRLGVRNIMLLFCASYAADALASAIDVLSK
NERELMYPAWFPFDWSANRFTYYGAVFYQIFGVSLQIVQNLAHDTFAPVGLCVISGQVRLLAMRVSKVGYDESKSL
AQNEQELNECIEDHKKLLRIFDLMQDVFWYTQLVQFSSVGLNICLTVVFLLLFVDNLFGYVYYTVYFISMAIELLPACY
YGSNMQEEFQNLPYAIFKCNWIPQRRGFQQNLRIFTELSHKQLTPTAGGIINIHLTSFVATCKMAYSLYTMLMNI

>CcapOr31

MFQRQIETRAIFRRLFMTWRVLGIILWPFNKYLRIIYDILMNIFITFAFPVHLTLGVIFSSNQEQFFTNLIIGIASVSCTFK
HLLWRSRLAEMQQINEILAQLDDRVRVREDYEYYKRSIERLCNFMINFFTRCYFSVGVTALFIALITGELLYPAFMPLQ
WRTSFWNYVAAILFQFVGVMLQIVQNIANDVYGPVVLCMISGHVHLLANRVSRVGHDTEENTQSNYEELSKCIED
HKLLMSTSKTVERIASLSYLVQFVAVGINLCIGLVYLLFFADNYFAYVYYTIHITAIMIELFPCCYFGSMLECEFHDLSYAI
FSSNWPTQPRPFRRNVVSFTEMTLREVTMYAGGMIRINLDSFFATCKMGYSFFTVIQTMK

>CcapOr32

MSKSKVDAVSIFVRLIFFWRVLGFTTNYNKHLVRIYDVFVTIIVTLAFPMHLMLGVIFAKDKETIFINLAIGISSIACTAK
HFMLRPSIDKILYVNNILRQLDERVQNVADTDYYVKQMREKAIFMINFFIVVYFSVAIMALLSALWTGKILYPAYVLL
DWQSGTWRYLAVMTFQTFGLNMQIVQNLTNDAYGPMVLCMLSGHVHLLSNRVIHIGHDHETDVDDNYAELVRS
IEDYKLLMSTTKLVERIISSSYMVQFTAVGVNVVVGLIYLLFFADNLFAYCYYLFHIVAIMIEIFPCCYYGSMVQAEFHA
LSYAIFRSNWLTQPRKFRRTAITFTELSLKDVTMTAGGMMRIHLDSFFKTCKMGYSIFTVLQSLK

>CcapOr33

MAFEENPESVGSLFRTHWIVWKCLGQVPDPRYPKLFKVYAVLLNVGFGLGYPLHLLLGQLGLQTLEEVLLNLTISVPV
AVCALKFFNIWRNLRKVRHLEKMFNTLNTRINQRDEWIYYRKVTIPNALKVLHLFYFICVGTALASELTLLIMGFAYE
WRLMYPAYFPFDPYATTGGYVVAHTFQIIGLLVQLAENLVSDTYGGMCLALLAGHAHLLGKRVAIIGYDNQKTEMD
TGRELANCIVDHNMLFDCHSILGEIIGIGMFAQIISASLIMGIVVIYMVFYVGNAFEYVYYSIYLFGCAMEVFPTCYYAT
NFEFEFDKLTFMLFSCNWMDQNQSFKKSLMISIEQSLKTRSFRVGGMFRINLQIFFATCKGAYSVLALALKFK

>CcapOr34

MIQSDKISTIMSATLEETFVTGLSVFHFHDVTWLYLGQMPPSTPLYRYLYYLYSLLLNIIITIGYPTHLMIGLIRSEKQSD
VFKNMSINFTCLACSIKTFAFWWRLAEVQKIYAIISKLEKHISELDDFRLYKTIALGRAKSILYFVLFIGLGAAITSEVATII
AGILGDWHLMYPAYFPFDTDRSIFGYTTAHFYQCFGVTAQIFQNLINDTFPPMALAMLAGHVRLLNLRVARVGHA
SSGGSTKQTHNAQFIECVEDYKDLLEFRIAIQRICSLGTFVQILVTAFNMGVVIFYLIFYVNDIFSYVYYVVFLLAMPLEV
FPLCYFGTSAQMEFEQLSYAIFSCNWVAQNTAFKKNLLIFTEQSLRKQIVIAGGMFAVNMDTFFATLKFAYSLFAVV
VQMK

>CcapOr35

MCSARLLQINDNGTPLNSAHAFRYLWLNWRLIGMHATRRHRLAYHIYSGFINLTFGILLPATMIAKLFFIENLSQLIGL
LYLGVTLTMATAKQYSLWLHRPQLLAVNSYLAKLDARCDRHAVDRQHILTAIRICHLFYFAYMFTYELSSSGFAYMG
FSLRQLVYEAWFPQFFADPAKNLTLTLLYQNFAVMTFFVLQNVNNDMYPQCYLAIMIGHLRALAARISRIGKDDGR
LSDEENIVELINCIEDHKNLLGYFACIGPVISRTIFMQFGITAFVLCLTAVNYVAFERDTAQMLIAATYIFAVLIEALPCC
WYVNSLMEECAQLTTAMYECRWYEQNRKFRKMLIIFMQRSQLTLALMAGNLVPITLQTFVNIIKFSFSMYTILKG

>CcapOr36

MSNKAATAAQSTTPLQSSDAFKHIWLCWRYLGMHPTTRHRHLYLISSLLVHLFTGLLYPSLYLASIFVDIDFSDKLANL
SVAMPLYYTAVKQLVMFYYIQTDLPHATKHLQALDRRVEERPEDYVYLRQTVRYGAWSFFAIFLGFWSALISYGLIGL
LRHRLPFEGWLPFDWKNSMGAYVGAGLIQLFAVGMLLTNAVCCDSYPLVYLSLLVAHLRILNKRIARLGTSRKATAV
EHYQQLAACVEDYRECMSYYQCIRASIGGTIFVQLVSTALSLSTPAVTFISGDFNFSQMLKFLLFSSAVIVEAAPCCWL
MDVVLLEMRSLTHSIFSCHWHVQNARFRRSLIIFMQISQKVDPLLAGHIVPVSLDTFTNIIKFAFSLFTLLNQIKSK

>CcapOr37

MTKQEQATPTRLDSGDATRYVWLFWSIIGIHPFKKHRTLYWLYSVLLNFCCSAFFIAFYAVTFFVVSDLLEILANLSV
MVPLIYNTAKQLVIFYHIRRTLPQAALHLHALDRRAEQEPAAREQLKRLVQLSHRIFLTALTGIGICLTLYAMGGILRH
RLPFDGWLPLDWEHSVGAYVAACAYQLFCLIVQCIAALCNDTYTVIYLLLLATHLRILNARIASLGHGECTEVENYRQL
AACVRDHWACMNFYNSIRPAIAATLFIQFFSTAITLCTSAVAFVNAEDSVAQLFKFLPHLLVVVCEILPCCWLMDKA
ALEMQDLTKSLFACRWYEQNQKFRRSLLIFMQRSQKVEKILAGDLVPVSLETFVNIIKFTFSLFTLLNQFK

>CcapOr38

MRKIGDLCYGRGKNNVYIKESFRLLFFSWSLTGIAPTKTPRLFNTIFMIICWCGILMCPYCFIAGAVNSMKTSVITVTL
VNLQAALNGIALPLKAITIAVNVKRLRSIDNIFKELDNSYTDPVHHELIKKSVMRCTRLFVVFLTVYWLYGITSCTAALF
SHKYPHSMQIPFIDWLPDSDVKYWLHYILEASYFFFLLLVNLTNDVFPAIYIKAIRTHLYLLTERVSTIGKKSETTAEQNY
DTLVECIISHQKLLRISDTVGDVISKSIFFQLAVYSTILCICMLNMLIFADTTYILVTLVYLIPVLSQTIPSCYQASMLEAES
TKLSVAIFHTNWWNLDKRCHKLLIYFIQRSQQEMVFTAVKLFQISLKTNLTIAKFSFTLYTFINKMGIGETWKN

>CcapOr39

MRKFSELFYGEGKENFETNESFELLYWNWTFLGTRLVKPYRVRNLMSSAFAWTCLLTSPLFFFVGIAKMVNTSSMT
ELLTLIQAALNVITLPLKTIVIAYYMKRLSSVKPMFKRLDERYNTPREREQIKESVKYSTRIFAIYFIAYFIYGTMTALLGLT
LHSQPLNSWLPFTDWIPMQTLRFWLHFCYEQFTVYLLLLNQVSNDAYACVYIHALRTHINLLAERVSRLGTNSEFDD
EQNFKELIDCIAAHQELLEIVKTVANVFSLTVFMQFTVAAAILCVCMLNIFIFADTFHQVVTVIYYMCVSLQTLPTCYE
ASMLEAESAQLALAIFHCNWVDMDKRSRKLLIYFIQRAQEEISFTALKIFQINLRTNLSIAKFSFTLYAFMNEMGFGE
NLKDKK

>CcapOr40

MPQLSASIVISSKTGFNNWLVGKIICKINKKFFRNQAKMDKLRAVIFDRVGLSTKDSFDLLYLNWWLNGNTSWKPH
RLGHILHMTICWCLKFFAPVTYFKGFLIALSTSTITTALYNLQATLDVMVAPFKAVVIAKHMHRLRTLTEVFNRLDDR
YHNPRERAQIDEGVIICRQIICFYCAVYSGYAVMTWLGALIAGKMPHYLWFPYFDSIPNETLRYWLQFTFEALFIHFM
LNVSYTNDVFPVIYMRALRTHVKLLAERVSRVGSNPELSAEEHHRELVDCIVAHREILYIVDVVGAITSLTIFLQFAMA
AATLCACMLNVLIFAERIGQIITIIYYMGVLLQTGGSCYQASMLEAESSSLATAIFHCNWLNLDKRSRTLLVYFMQRA
QEDIAFTALKLFQINLKTNLSLAKFSFTLYTFMNEMGLGNDLAKSQS

>CcapOr41

MHRVMEYIFGRRRLVVKSGTNDSFELLFLIWKIIGVEHSRSYGFFQLFHVFCCWALLLYSPAAYNMGFLRALKTLPM
ASALNILQTDINVSILLFKVVIIKFHLKRLRSLRDIFKRLDERYHNPEERAQIDESVAICRRIIYIYIFVYFTFAFLSWITAIM
AGELIYSLWLPFVELIPHQGWQYWARFSVEAFYLYFLILVCLICDVYPAVYIRAIRTHVHLLAGRISRLGSNPDLSAEEN
HQELVDCILSHQELMRVVEVVSAVTSLTLFLQFTVAAMILCVCMLNALIFADRAGQIMTVGYYMGVLLQTGGACF
QASMLEAECVKLPLAIFHCQWLNLDRHSRSLLTFFMQRAQVNVCFTAIKLFQINLRTNLSLAKFSFTLYTFMNEMGF
GGDTNEKIS

>CcapOr42

MSDLLFGRGQIVYKSRHALTYLFNIFTFVGTNPLGGQSYAYYSLYYLYSLLVNFVCCIFCPISFHIGYIKLLNVLNTNELLS
AIQNAIQVSGIPIKIIVIAWYMKRLQSVCEILDKLDENYKRAEDLNSIRRCVRSCCKIIAAFCVPYYGFELSTIAFGVYQN
RAPLTIWLPYFDATRTTWEYWTHVGWDVFIMLFLLAHQLGSDTYPPVFISVIRLHMQLLVERVKRLGTNKALCREK
RYAELLVCINTYGQILSIANIVAPVISITLFTQFATTATTVLNWFGNMKFPDNIIPMAFFSCQIMQILPCCYYASQLIAD
CEQLPYAIFHSDWLEEDRRYRKTILFFLQHTQTPIRFSCLKLFGVTLATSISIGKFAFSLYTFIQGKNH

>CcapOr43

MLDLLRGRGRRVYKSRHALTYLFNVFTFVGTNPGKTRTHKYYTLYYTYSLTVNFICCLFCPLSFHIGYIKSWHLLNTTEL
LAAIQNAVQVTGIPIKIFFITWNMRRLQSVIPILDELDENYKSAKDLLKIRKCVRGCCKMIGFFCLPYYSYEITTIALGVW
QNRAPLAAWVPYLDGQRAAWEYWTIVVWDIFVMFFLLSHQLGSDTYPPIYINIIRTHVQLLVERVELLGSDKTKSAE
EHYAELLGCIRTHGQIKRIVNLIAPVISVTLFTQFATSATTLLNWFGDVEFPENIISFAYFTCLTLQILPCCSSASYLISDCE
LLPNAIFHCNWIERDRRFRKTLLFFLQRTQTPLRFSCLKLFVVKLETSVAIGKFAFSLYTLIQGTEVGGKTEN

>CcapOr44

MFELLSGRGIGNCPSSYAFIYLFNTFTILGTNPPSDAGPLYYIWSAFLNTFCIIFSPFLCTVGFIMKYMQSTITTMQFLS
GIQAGTNVLGIPPKCLTLAFSLKRMRSIEPLLDVMDARYTDPEDVALIRQAAIMGNRLVFGFGMTYLTYMLLTITPPL
ISGNVPLSIWIPFLDENQSTLHHLMQVVMDLFLMFFLLFHQVVNDSYGTVYIYVIRTHLRLLIRRVERLCVNGEKSVE
DNMAELVDCVTTHQQILSLLTIIEPIISVTMFTQFLIIAIILCVTMVNMFIFADLSTQIASTFYFMCVLMQTSPCCYFATE
LKADSERLPLAIFHCRWMDQDQRFRKVIIYFMHRAQSPIELMAMKLFPINVATNISLAKFSFTLFTFIKEMGVGQDA
RE

>CcapOr45

MFDLLKGRGYRELNSRDALIYLFNMLSFVGLNPTAHCRLLYYFYGSIITLFVVVLSPLIFNIGWIRDRNVLSIMEILNCV
QAALNVIGVPIKSIALMLCLDRIHSVEPLLLKLDAHYSKWDDMLRIRQCAIMGNRLVFSYIVPYMMYETLTVVSAVLG
GHAPLTLWLPYVDWHRSSREYWLQVCFDAITLFYLLCHQIINDSYPAVYIYIVRTHVQLLERRVSRLGYVPQKSEHEN
CQELQECIVTHQEILRLVHTIQPIISITMFVQFIIAAAIMSITMINIFIFADLATRLASFVYLICVVLQTAPSCHQASYLQG
DCEKLSSSIFHCNWIAQDKQFKKLLIYFLQRSQADMPLIALKMLPINLATNVSIAKFSFSLYTFIQKMGLGAHLND

>CcapOr46

MFDLIKGRGRTVFASRDAVIYLFNSFRYLGINPPDKYRIPYFLYSAIITFFAVLFSPVIFNVGWLRDRNKLSVMEILTCV
QASLNVMAVPLKCITLAMAQNRLRSIEPMANELDDYYRQPADKVKIKKCAVTGNRLVFGFAVSYLMYETLTVVSAL
VGGHAPLSLWIPHVDWHRSTWEYWLQVSFDAAVLFFLLYHQVLNDSYPAVYIYIIRTQVQLLAHRVENLGYDETKS
DDENYKDLLECIVLHQKILKIVSIVEPVVSVTVFTQFLVAAAILGVTMINIFIFADLTTKIASVTYFFCVLLQTSPTCYHAS
YLLADCDELRLSIFNCNWIAQNKRFNNLLIYFLHRSQDSIPFFALKLVPINLATNLSIAKFSFTLFTFIQEMGLGENLKG

>CcapOr47

MFDLLKGRGRSVFASRDAVIYLFNIFRFLGLNPPPQCRFLYFFYGSIITLFAVLLSPFIFNVGWIRDRHILSIMEILNCLQ
AALNVIGVPIKSITLALSLGRLRSAEPLLMKLDARYTDPEDVARIRGCAITGNRIVFGYIISYMMYETLTVVSALMGGH
APLTLWIPYVDWHRSAREYWLQVSFDAAMLFFLLFHQILNDSYPAVYIYIIRTQVQLLTNRVRRLGTGGSSRDDAYH
ELQDCIITHQEILNLVGVVEPIISATMFVQFFIAAAILGTTMINIFIFADFATRIASVTYLFCVLLQTSPTCYYATHLQSDC
ERLSMSIFHSNWLAQGKRFNQMLIYFLHRSQADIPFFALKLVPINLATSVSIAKFSFTLYTFIQKMGVGKNLKQ

>CcapOr48

MNRFRKQSARAAETENNKLALALSKEKLSGLKNGEEQRIARKPAKPASAVREKQGEFVASPASSKEATNYFFKAAFG
MGIMLPTRHRILYILYSFAVNSMATLYFPIGFTLIFFTLPEDDLDVSNLLTSLQVTFDVYAGSIKLIIMAFLLGKLRTSEIV
FQQLDNRCRTPDEMNELRKMQQFGRKVIIFYMTIFLIYSSSTFLGSVTFGYPPYSLYFPFLKWRRSRIEFIIASLLEFLIM
DLACLQQTVNDGCPVVYVNILRTHMKILRSRVEKLCTNAALTKEQNLLELKLCIKDHQLLLELYEIIASIVSITLFLQFTV
SAICVGTTLINFVIFANGFSTRVACFCFILAVLIEIYPICYYSQCLITESEGLSDVIFHSNWIEQNKEYRQLLIFFIQNAQRP
MSLTAGKLYPVTLSNFISIAKFSFSLYTFIEKMNLKERLGIE

>CcapOr49

MADTAINSQRLQTDTLNNGLKKKGDQLAVRTEHATNYLFNGFRVLGIYMPARRKWLYSLYSLIPNSLVTLWLPLSFV
FSYFTMSAEDLVPSSLLTSIQVAINVIGCSVKIVVMAFLLPKLRKANAYMDRLDARCKDEDEIAELRKIVKQGNRFVVL
FAMSYWSYASSTFIGSVTFGRPPYDLYNPFIDWRKSKLEFVAASLIEFALMDVACFQQVVDDSYAVIYVCILRTHMNI
LLKRLGKLATCTEMSLEQNLEELKLCIRDHKNLLGLYNIVAPIISITIFIQFMITASILSATLINIFIFANQFSTQVASCFYILA
VVVEVFPLCYYAQCLMDDSDRLSQQIFHANWIEQDVRFRKMLIFFMQRTQRVMELNAGKIFPITLGSFLSIAKFSFS
LYTLIEKMGIRERLGLE

>CcapOr50

MSNLLQRLLNQLLPSRTTQKSIDVVKQVSPSLSNAELIRIQFERATRTPKERSAPVGRPYQAVHDIHSRDGLIYLYRSFS
ALGVLMPDKHKILYCLYALLPLGLITFYLPISFALSYFYLDYSTVKIGNLLTSVQVFIASIVGGVKLIVMAFKLPKLRASEAI
MHQLDARCKDEDEIEVLRKVVRQGNRVFVLVLICNLIYSTSTFLAAASKGRPPYNLYNPVVDWRKSKGAFLWAALW
EFILMDGLCTEEAITDSYAPIFVCIMRAHMKTLLMRIQKLGSNPERTLDENYEDLKMCIKDHKLLLELFDVVHPIISTTY
FLQFMTTSLMVGCTLLNIMIFAVDNLARVGHLAYVMALLMEVYPLCYYGQSLLDDSNRLANTIFHANWIKQNEKFR
KMLVVFTQHTQKPMELLAGKLIPINLTTFVSIAKFSFTLYSFINNMGVKERLEGL

>CcapOr51-CTE

MSTDKPEPQANAEHMGSPLDVQRRDMFTFIRWSLWFTAMCRLPLEYYLPTSLRFLASTLNWIYEVFLYFTIIHIDILF
MCTIYLYKDKGDLALLVNSLIQAIIFMWTLIIKVYFKRIKRKKIEELMQFLNNEYRTQSAAGFTYVTMKESVDLSNTWT
KVFLISCYVGGAFWLFVPFLRRDRSLPLPCWYPFDYKSPIVYESIYLLQCLAQMQMAAAFASTSAFYLLVAVVFSGQL
DVLNCSLKNVIATTYLNLRKPKSELIKLREEHNIENFEINQYYCAEEHKTDLDCLPHLLDGENPEPQNFYAAFKQAFKH
CVTHHQYILCGLQMLEDIYSYLWYLKTMKATVLACLFA

>CcapOr52

MALADRKTPPTDTEHIGCPVYSTQRRDMFRFMRWNLWFTAMCRLPLEYYFPTCLRCLANTLDWTYEVFLYFTLLHI
DILFMCTIYLNKDKGDLALIVSCMIQTVIYTWALIIKVFFKRVKPKRVAKLMQYLNNECRTHSAAGFTYVTVKDSVELS
KNWISIFLICCYAGVTFWLFVPIFNADRSLPLACWYPIDYKANVPVIYESIYLLQTIGQLQIAGAFGCTSAFYLLACVLFS
GQFDVLNCSLKNILATAYINLGKSKSELCKLRDKQYIADKELNQYYCSKEYKSDLDCLPHLMNVATPKPKTLYAAFKQ

AFIPCITHHRYILYGLQMLEDIYSLLWLLKTMEVTVLVCLVAFAWVKSTTAKSFLSILSLSQYLLLALWEMFMICYSGEII
FLNSQRCDEALQRSPWYLHANEIKQDTLFFILNAQRPFRLTGGKMFDLNVEKFRSILTTSFSILTILQKMDVRPTQPK

>CcapOr53

MILTDAPTAHAAAQLCASPNGCLSNVKRRDLFRYVRWLMWGAAIRPIPFENHLPRRLGNYSAIINVILEIFLLLTVIHI
FVLFILTLYLNYGSGDLEFFIGCSIQSMLYFWAIIIKIIFRRVRPELVRDIMDYVNEKYIVHSAVGFTYVTMNECLEQAER
GIKYFVLSSLVAVIFWLFQPVVYEERTLPLPCWYPFDYKAPFIYPLAYFLQVIAQLQLALTFVTNSIYFTVLCFLLCGQFD
VLNCSLKNILATTYILMGASRKDLIELREHQCNADDEINQYFVAEELHINLDCIPHVLTPATTAGTMNFRDAFHCALG
QCVDHHIFILNALRKTEKLFSMVWFFKTLEVTFAICTIAFDVVKSTDDKSFLQVLSLGQYMILVLWEMFMICYGGEIV
YINSQRCDLALLRSPWYLHSREMRAEILFFLLHAQRAFALTGGKFYPLKLEKFQAILTTSFSFYTLLQNMDQRN

>CcapOr54-PSE

MQNTAGACSIILYSEKDKPRVCDLFMAQVLAFKATGQIPFNWRWRLGYIYCFIVIAQTFLLAVLFLKSSYIMLLSGKLE
EITDALTMTIIFWFSVYAACYWLLRWRRLMAFLELINQQYWHHSLPGLSFVSWQRTYLLAKRMTIVWTVACVLGT
VLYGLAPLVMGVRALPLKAWYPFDPLQPYVYELVYVMQLSAQIIMGATFGNGSALYVSLVILMCGQFDVLYCSLKN
LSHSARLRCCSGVEILRKEQAALPKSPDDELNQYMYCREHLTNLSILQHLYTQQPALTLPEALHLGVVQCVQLHRFIL
DACKELEELFNPYCLVKSIQVTLQLCLLVFVGVAGERSMVRIVNLAQYVTLTLVELLMFTYFGELLRGHSVRCGEAFW
RZQWWTHTIPIRQDILILLANSKRAVRLTAGKFYAMDIERLRSVVTQAFSFLTLLQKLAAKNQK

>CcapOr55

MLHECSDKSTISNTIFNHQSYRRRSRKYSSSKLETILAPIRTLKEMLLSGDAAHPSHTCLYYVRAYIRLLGLWPSQRGVE
QPMYYAYNVLIMTVFSFFVATIIADLYVASSDFVLLGEDLVVALGLYLILFKMILFRMSTADVDVIVDEFDALHMKFA
RDTSDSPHIRRIRQLQRSFFLGEASFFCGFFFLSLFLFAAMSLQPLLTHQALPFRCVFPFGLHDPDKHHITFVCVYAFQ
CFCTLYMLVSIVVMDSLGGNSFNQTTLNLQILCESIRHIGYAGGRSTTITEAVLWRELRENVEFHVKIIELVDGINHTFY
WNYVSQMGASTFMICLTAFEALLAKDQPMVAMKFQTYMFSAFMQLFYWCFMGNRTYYDSMEVATAAYEVYA
WYEHSPRLQRNLLFMIKRAQKPLEFRSKPFFGFTFASFNSILSTSYSYFALLRTMND

>CcapOr56

MIRAQQLNGHKVPSDVVTMILEEVEDVYKRNYNSIKVLIRVSFGLGVNLTAPTRFKDSLRIFNVILVTTSILSLYAHWC
YLIRHFDNIPLLAETVCTALQTLISAVKMVYFLFTQRTFYRLIDQALTHEVIRKIEIFKYDFPINRQLKQEIDDIMSGVWR
HARRQILFYFCCCIGIVFNYFFGAFFVNLYHQLKQTPNYNFILPFPALYPFWEAKGMTFPYYHLQMYMTGSAVYIAGI
CAVSFDGVFIVLCQHAVGLVKVHNLLVLRATSPLIPPERRVDYLRYVIFTYQRVRVYVQQVQTIYKHVSLSQFVLSLIVF
GFVLFEMSFGLESSITIFIRMIMYISAGGTQIIIYCYNGQQLTSVSEEMPLAFYSCGWYEESKKFKQLLRMMIMRTNR
HFYLEVSWFTLMNLATLIALFRMSGSYFLLLRNLQES

>CcapOr57

INVLLIIASCIALYPHWLMIEHAEGNLSLIAETSTTALQTTTALVKMAFILFKQHRLHELLYKAEYHELLQGIQIFMTDM
PIRISLKNEAIRIMDTTWQEARGQLLFSLISCICIQANYFFYAFFKNLYHHLQGTPNYVYILRMYSFTGYPMFHHKGM
SSFYYIMDMFFGACSLHCAAMCAICVQCTFMVLCKHCCGLVQVQCLMLLRSTSPLVPKVRRVEYLRYCVIQHQQIL
RFMEGINQLFRHICLSHFLHSLAIYGFVLFEMSFGLESNKVIFVRMMMYLCAALTCDCMFYVNGQFLSTELEKIPLAC
YSCEWFHESREFKMILKMIIMRSNKPFYFQISWFTVMSLATLMGIFKASGSYFVLLRDIDEA

>CcapOr58

MATKTIRPTQKFAKMIKIVRFISSLVGADVADENYRINIVTVLVILCIIIYFIFTGTTVASVFAENWKYLLEASCMVGSVL
QGITKLISGVGCTKMISSICKELENLYQHYETKGEAYCKVLNEGCERVWYSIKMVGHIYAAAIYGILLLTGFLILTTNEK
VYVMHFFIPGVDVETTYGYLFTLAVHTVVFLAGAFGLFAGDLFFLIFLGQTQLFRDILVLKVKALNEAAAENAKNTESL
LIDIIEWHQYYTDYNKRCNDVFYYIITMQIVTSGISIICTMYILLMGDWPGAYLYIFVAFCGLYLYCIIGTSTQTCNAEFF
DEIYNINWYELDVKCQKMMVFIIKKSQSPAEIKIGGVLPLSVQTALQITKSIYGLFTMIIGVIEENN

>CcapOr59

MAAEKVSPSESFAKIVKIFRLICSLVGADVCDVNYRINIVTAIVIFCIIIYFIFTATTVASVFAENWEYMLEASCMVGSVL
QGITKLTSGIAFAKEICAMRFELEDLYRLYETRGEEYTAVLHLSCKRVWQVIKMVGQIYLAAGVGILFMTAIFIVATDE
KVYIMHFFIPGLDVHTQMGYLLTMAVHTVVFLAGAFGLFAGDLFFLLFLGQPMLFLDLLTLKVQALNVAADRCSNE
AERLLIDIIEWHQYYTDYNRRCNHLFYYIITMQIVTSGISIICTLYILLLGDWPGAYLYIFVAFSGLYLYCIMGTKIQICND
AFCEELYNIDWYKLNVKSQKMLVFILKKSQKPAEIKVGGFLPLSVQTALSITKTIYGIFTMMLRFLDEEN

>CcapOr60

MKSQVKRTLEPSESFSKIVSVVRFCSRLIGVDVFDKDFKINPKTYFVMVAFAAYYLCALHTLSKYIATDWTVLLDIFSPV
SCTTQGLVKFISALLYPSLYHRLSVEIGDIYEKYQWMGKKYEDKLMEWNKSMKKILLSCAILYFLTALIVICTPVVLYVL
KGERHLTLLCEVPGFDVTTTQGYLVTNGFNTVCILIAAFGLYAGDLFLFIFLTHSIFFYDILALKISDLHEIIEENDRDERL
GKMVDDIVEWHQFYLGFNDTCNLLFFWTISAHIVCTTLGILSTLLIIMLKDWPGAYAYILVCFLWLYMYCLLGTRVEI
CNDQFCTGIYDINWYALNVRNQKIVRLMLMQSQAPRNITIAGVEPLSVSTALKITRTIYSLVMMVLRFQSK

>CcapOr61

MQQPTTKRPSDKYNKLLSIIRFSSTLIGVDVIAENYKFNWVVGFVFAAIGWNFFCSSYTIWKDVTTDWTVLLDVFSPI
SCAAQGTIKLFSLVCYPKLYRKLALDLGEIYEKYQLLGQKYEKKLLTWNKDMKRLLIIGGLIYFASAWIALITPLGLYILKG
EKHLIIMCQMPYIDGSTNQGYFILIGYNLICVFVASFGLYAFDLYVFLFLTHSIFFYDIFALKVDDLHEVLRQNDKDKRLL
SLVNDIAEWHQYYLEFNDQCNLIFFWPITSHILCTTLGILSTLLIIMLKYWPGAYPYIFVCFVWLYMYSFLGTRVEICND
QFCDGIYDIKWYDLDLRNQKTVCLMLTESQVPRIITIAGVEPLSMNTALKITRSIYSLAMMVVQFNE

>CcapOr62-PSE

MFSPKQPSENHAQLIKVLRFCARQIGCDVLEENFKFNRTMGAVLIAIIWYYLCSVYQIAKDFATDWTILLDVCSPVSC
TTQGLVKLISVLLYPKLYYQLAGEIKETYEKYEKMGTKYKDILHRWNKAMKKIIVFLAMVYLLTAVLMLSTPLALYIFKG
ERHLILLLQMPFTDVTTTRGYLSATIFNVMCIFIGSFGLFAADSFLFLYLSHSLFFYDIFAQKIEDLHELLEHNRQDERKT
ALMNDIVQWHQFYLRFTDNCNLIFFWTISAHVICSTTGILSTLLIIMLKDWPGAYVYILVCFIWLYMYCVMGTWVEI
V

>CcapOr63

MNAIKRNTNFLRFTAGPVKYFKVIGICLQPPETLSIKFARFLTVLTTLLLFVHQIAFLVTPGRTFVELSAAVGLLNYTTVG
AGKILFLILNRHLLLKSYTQLQAIYPSEAVERHYKLDRYLLIYKRVETLLYNFFKYILIVYLVSPIVQSFYDLWSNGAYSYR
MPTIIWYPVPLEESLLEYVVYLIFESYTSFSLGTIILSADLCLFSSVSQLMLHLDLLAQRILELQPAEQGSMNDLKAIIEYH
QRILTLAQDVNSIFAPSIVFSLASSSFILCFSAYQLLEDVSFIFALKVLLLLGYEMKQVVITCYYGDKLMDSSANLFNVVY
AHDWTDGTPAYKRLVLIMLIRTYRPIALNVAGIADVSLITLKQVLSTSYQIFAVLKTA

>CcapOr64

MALQAGRGGEPKLRSIDDLCAILHPIQRYLSINFLDFTRINGRFAIPSSMLLNVGIVLSVLDCMGNITKVCMAINDRDL
TKAQETFAVLGMAFVMTMRGMMLARSRVRLSELYNSIDRIFPNSSELQTHMEVAKTHDYIKRRFFLLHQGLSFALV
LFCTMPAVKLVFFYDFEAQEPVADEFHVNPSWVPFQVKETISSYGYIYVYEVILALVAVNMIITWDEVFVVLISQLCM
YYQYLAKLLTALDVREANDPKKAVAFFKRLHLYIYIHQYLNSLADELNDLFNLSILVSDMGTAMSICFNLFLVTGAKDY
LQIPSYLTPCFVETWLIYDVSKWGTMLETVTARINEVLYEQKWYDSSVRFGKYTTMWLQGTNEPIRLTAYHIFDVN
MKHFQDMMMLAYQMLTFMKSKS

>CcapOr65

MHFRIYNIKDYLIYPEFAFKLGCFEPFIWSGNRHRHQQTVSYHLKSVICIFDASTVICQIFALVISLFVPRTTAEAELYED
SESRVFEAVALICYFACGFYKFWNIFWRRNDIGLVLEELKNLFPSVSKQKMQAEAKANSGEKNDAIACYRLAYYEDK
SRIMMQRLTRYFMFAYFYYNIIPILQLFAEVISHQEVITFKSQSNAWYPWHNHNHHSTFVGFVFSFLIQASAEFAAIS
FIMSGEFLFCFLNTQLQLHFDYLTGALSALDARSPNALLHLKTLINYHNQLLRLSKLINSIFNFTFALDLITTTFAISLMGL
TMVMVRFGQAVMFSAGFSFFLLLGFLFCNNGDELINATKNLGAAIFYSNWYEGSSEYRRMIIFFIMRTKTPCEYRAF
GYMSLSMETYMRILKLSYQLFTSFRAIE

>CcapOr66

MSGVFSMNHFLKYPNFTLDLAWSNPFAWSGARQYGYHMLWIRRLLFTFGAANLVYQNFGMMIYLCMPHEVSN
ESTIGQITETGGIMGLTMVGASNMFVMYWHADRIALLLEKFQRLFPTKQLQWRAKHANGRLRGVKFPHSVEYFAL
KSNKLMKIATTAYLFAFSYYNSLPIVEYLYESLTPGVELKYHYQSNTWYPWQNAHNRKSFIAFLAAYICQVQSSLTGV
AFIMAAEFMLCFFITQLQMHFDYLANALETIDAAGENANEELKFLIHHHGRLLSYSKEINAIFNISFLVNIFTSSIAICLM
GFSMVMISVIHACKYCIGLLSFIVFTFFICYTGKELTDASDKLLYAAFYGNWYEGDLAYRKMILFLIMRCRIPTVLRAYK
FTTVSMPTFTAILRSSYSLFTFFQAMGK

>CcapOr67

MDFEKIFWLPNTLYLVVGYDFRQVSKSYLKKILMTAFLILTNITGICIRIYMLIQLRELVLSGDMLNSFRLGVYISYAFDSI
VKFFGFLHNAHRLRKIYESLATEFPQTFSEQQFYQVHKYSFNRSRILIFAYLSVTNSILLGPIVQSIIMYIIDAFLYGLSGA
KFQCLHPTPITYNFNFCSPRYYIPIYIVEYLNGHFLTTTSLGTDLYVCTFAAQVCMHLKYLGNSLEGYEPSADNSKADC
AYLKEWIKKHQLMLRLCADINDVFGTTLLFKLISNCTVFCIIVVQLKLEGFGWGFLNFLCFFFVTVAQFFMVCHFGQK
LINTSEDVSLCAYKNRWYNGSKAYKTLLFTIIARSQKSCKLTAKGFQPISLQTFQIVMTMTYRAFAVLQRALD

>CcapOr68

MAPYFHTREPAATIPDFVGIPFFLISLNGMQLFKWTPNEEASRRKLLLITAFSVIVTYDCVSMLSVFAFVKLERLDYTTF
ALYWGYALNSLMKGGTLWFGRRQLEFILKSMVEKHPKTIAERQEYHLVAYFTKIKSFNKYLTIFHLCTTSLFNIQPMV
SSIVEYMGRQDKEEEFKYKLPFIMYYYYNERQPVLYLFSYFLQCMGGFYMSYLFLGGDLLLMTLVHLVNMHFEYLIR
RIESLQPTEDSEKDLNLLGPLVTYHLEILDLSILLNYIASCLCLCLLGLQIVMGSDLVTVVKFFAFLVSTMVHVYYISHFG
NNLIDLSTGISDAFYNHPWTNAKYKYSRMLVLPIARAQRYAHLTAFQFFEISMHSFKSLCTTSYQFFTLIRTSLEEDFH

>CcapOr69

MPFAFQQLCFELQLSLKYSVPAMPLKLANNEPAATIQDFVGIPLFLLTFMGVKLFKWTPEEASSKRQLIMLGVFCVF
ATYNFATMILYIMYEPLNSSLDITEIILFWGFSLNGMMKLAIMILYRNELKSILRGLGARHPQTAEERSIYRLVPYYNKIL
IYNKYLAAWHLSITTLFSFHPLVASILGYIFRRDSSDGYDFTLPFMMWYYYDTTKPILYIFSYVVQTFGAFWMSLLFLS
GDLLLISLVHLVNMHFDYLIRHIESFQPNGTDEDMKVLGPLLAYHQEILDYAERIDSTFSLGTLLNYAGSCLVLCLIGLQ

IVLGSEFLKVVKFIAFLVSTIVQVFFVSYFGNNLMDLSIGMSDAFYNHPWYDGNYRYSRMLVLPIARAQRYAHLTAFK
FFEISMDSFKSLCTTSYQFYTLLRTSLEEEAG

>CcapOr70

MPFAFQQLCFELQLSLKYSVPAMPLKLANNEPAATIQDFVGIPLFLLTFMGVKLFKWTPEEASSKRQLIMLGVFCVF
ATYNFATMILYIMYEPLNSSLDITEIILFWGFSLNGMMKLAIMILYRNELKSILRGLGARHPQTAEERSIYRLVPYYNKIL
IYNKYLAAWHLSITTLFSFHPLVASILGYIFRRDSSDGYDFTLPFMMWYYYDTTKPILYIFSYVVQTFGAFWMSLLFLS
GDLLLISLVHLVNMHFDYLIRHIESFQPNGTDEDMKVLGPLLAYHQEILDYAERIDSTFSLGTLLNYAGSCLVLCLIGLQ
IVLGSEFLKVVKFIAFLVSTIVQVFFVSYFGNNLMDLSIGMSDAFYNHPWYDGNYRYSRMLVLPIARAQRYAHLTAFK
FFEISMDSFKSLCTTSYQFYTLLRTSLEEEAG

>CcapOr71

MLPAARTFGEFIRIPIRFYQTIGEDLYEHRSPHRIRRLILKALLYIGFLNFNVLVLGEIIYFVKALNSFATVLEATGVAPCIG
FSFVADFKQIALTVHRQTLREHLDQMEELFPKTVRQQAQYKLPQRERVMRRVMGVFTLLCLAYTTTFSVYPALKAT
VQYWLLGAPTFERNFGFAIWYPYNATGKTWVYWLTYMGQVHGAYLAGVAFLSADLILVASVTQLCMHFDYISRCL
EDFAGASKKCAEEDIKYLQALVVRHAKCLELSEHVNSIFSFSLLLNFLTASLTICFIGFQVTASSTEDIVKYIIFLTASLVQV
FVVCYYGDELMTASLRVGDAAYNQNWFECDTRYKRLLIILILRSQKPASIRAPTFPPISFNTYMKVISMSYQFFALLRT
TYSDKGN

>CcapOr72

MSKPPIHFESFCYQANIFYTSIGLDVYDKPGERSTSKAIQLURQQLLGIFFAITIINMNIVLLSEFMYIFMAFVNNNHFV
EAIVLSAFMGFVFVGDFKIYSIWRRRTHITVMMRDLYALYPQTPEEQSNYEVKMELQRYSRYAFAFIMLYELSFWSY
NLFPLLNYLITDFLLGVRAVDRTLPYNCWTPFEWHTVNWRYYSMYLSQIAAGQACLSAQLANDLLLSAVAVQLIMH
YRQLAKKIESHKAGSTTGKQWKETPFERNVDLQFLCDVIAYHQKILCLSQALNDVFGISLFISFASTSMIICFVLFQITIG
ASIDLMITLAFFLFCSLVQIFLICFYAQKLLKASEYISYAIYNHNWFDADLRYKKMLVFIMKRAQKPAIMQASSFVMVS
MSTMTVLLQLSYKGLAVIRTIYAREPKGVSNK

>CcapOr73

MSTKIVTLDAFVEKASFWFSLYGVEAFDDFHDRSAEHHNWKWKNYLRKLFYYISFINVNWVLLHEAAFVVVNFLEN
GDFLQAARNLSFMGFVSVADIKILITMQQRTQLSKLMRKLYELYPKDVGDQRHYDLQRHLQHYSCISFLFSFSHTFT
VWAYNSLPMINYLIHGHLLQQKTVERTLPYSCWVPFEWRDNWLYYLLYTSQAFAAHSCLAAYLATDLLFCAATVQL
IMHFRKLAGDIKQYQPDYASGRQVGINTDLGKDLRFLSAVACYHHTVLEINQLINDIFGLPILINFISTSFVMCFLAFQF
TVGVPLDALITLTCYMICSLVQIYMICSYGQELITTSEDIGHAVYNHNWLVSDIRYKKLLIMIIRRSQKPAVLRATSFLN
VSMGTITDILQLSYKFFALIRTMYSR

>CcapOr74

MSIIIRFEEFLRLPSFFSRNIGIILWGQRGKLFDRFMFYFSSINLFLTLLAELWYIISTISTDFITAIMGLSYVSFVVLAEVKF
YYLIKYDMKVSTVLKRLNALFPHTKEEQENIQLIKYLKMSKFYTLFYTVTFMLVIWTYNLYTVSQRFIYTKILQVREIERE
LPYPAIYFWNWQDNWSYFMLYISQSLAGWHATCAQILTDLLICILISHLIMHYDHIARSLLNYQSKFAELYGKESTMK
CMPKLARVMMEERAVRADMKFLADIIAYHTELLSLTESLNDVFGVPLFMKFMSSSAIICFLGFQMTVNRGFDLLTKL
ALFFILSVLQVYLICHFGQLLIDASTNVSTALYSQDWTNADVRYQKMLVLIIKRAQRSATLKATNFIIISRATMTEIMQ
MSYKFFALIRTMYND

>CcapOr75

MCENIESFEAFLRIPSFFYRSVGVDLWNTNGGSIQRFIFYFGFLNVNLWLLSELIFAIITVSENFIQATMTLSYAGFVLV
GSIKMYFMWRKKTEMTQFLKLMDEIFPRTAEQQKMMNLRRHLRQSTIVMSGFALIFMILIWTYNLYPFMQRQIYD
CWLDTRSINKTLPYESYIPWNWHNHWSFYLYYVLQSIAGYHSAAGQIASDLVLCAMATQMIMHYEYVSHKIRSRYR
GERKCVDSKSVSCLNALNHWTEEQVATHKDMRWLCETIAYHSNLLSLSDVMNDVLGVPLLVNFMTSSFVICFVGF
QMTMDAEPDYMVKLFLFLFSSLAQIYLICHYGQLLIDASINVAAAVYDQDWFDLNVRYQHMLVLVVARAQKPAML
KATNFVRISRGTLTDIMQISYKFFTLIRTMYSD

>DmOr1a

MSKLIEVFLGNLWTQRFTFARMGLDLQPDKKGNVLRSPLLYCIMCLTTSFELCTVCAFMVQNRNQIVLCSEALMHG
LQMVSSLLKMAIFLAKSHDLVDLIQQIQSPFTEEDLVGTEWRSQNQRGQLMAAIYFMMCAGTSVSFLLMPVALTM
LKYHSTGEFAPVSSFRVLLPYDVTQPHVYAMDCCLMVFVLSFFCCSTTGVDTLYGWCALGVSLQYRRLGQQLKRIPS
CFNPSRSDFGLSGIFVEHARLLKIVQHFNYSFMEIAFVEVVIICGLYCSVICQYIMPHTNQNFAFLGFFSLVVTTQLCIYL
FGAEQVRLEAERFSRLLYEVIPWQNLPPKHRKLFLFPIERAQRETVLGAYFFELGRPLLVWIFRTAGSFTTLMNALYAK
YETH

>DmOr2a

MEKQEDFKLNTHSAVYYHWRVWELTGLMRPPGVSSLLYVVYSITVNLVVTVLFPLSLLARLLFTTNMAGLCENLTITI
TDIVANLKFANVYMVRKQLHEIRSLLRLMDARARLVGDPEEISALRKEVNIAQGTFRTFASIFVFGTTLSCVRVVVRP
DRELLYPAWFGVDWMHSTRNYVLINIYQLFGLIVQAIQNCASDSYPPAFLCLLTGHMRALELRVRRIGCRTEKSNKG
QTYEAWREEVYQELIECIRDLARVHRLREIIQRVLSVPCMAQFVCSAAVQCTVAMHFLYVADDHDHTAMIISIVFFS
AVTLEVFVICYFGDRMRTQSEALCDAFYDCNWIEQLPKFKRELLFTLARTQRPSLIYAGNYIALSLETFEQVMRFTYSV
FTLLLRAK

>DmOr7a

MAVSTRVATKQEVPESRRAFRNLFNCFYALGMQAPDGSRPTTSSTWQRIYACFSVVMYVWQLLLVPTFFVISYRY
MGGMEITQVLTSAQVAIDAVILPAKIVALAWNLPLLRRAEHHLAALDARCREQEEFQLILDAVRFCNYLVWFYQICY
AIYSSSTFVCAFLLGQPPYALYLPGLDWQRSQMQFCIQAWIEFLIMNWTCLHQASDDVYAVIYLYVVRIQVQLLARR
VEKLGTDDSGQVEIYPDERRQEEHCAELQRCIVDHQTMLQLLDCISPVISRTIFVQFLITAAIMGTTMINIFIFANTNT
KIASIIYLLAVTLQTAPCCYQATSLMLDNERLALAIFQCQWLGQSARFRKMLLYYLHRAQQPITLTAMKLFPINLATYF
SIAKFSFSLYTLIKGMNLGERFNRTN

>DmOr9a

MSDKVKGKKQEEKDQSLRVQILVYRCMGIDLWSPTMANDRPWLTFVTMGPLFLFMVPMFLAAHEYITQVSLLSD
TLGSTFASMLTLVKFLLFCYHRKEFVGLIYHIRAILAKEIEVWPDAREIIEVENQSDQMLSLTYTRCFGLAGIFAALKPFV
GIILSSIRGDEIHLELPHNGVYPYDLQVVMFYVPTYLWNVMASYSAVTMALCVDSLLFFFTYNVCAIFKIAKHRMIHL
PAVGGKEELEGLVQVLLLHQKGLQIADHIADKYRPLIFLQFFLSALQICFIGFQVADLFPNPQSLYFIAFVGSLLIALFIYS
KCGENIKSASLDFGNGLYETNWTDFSPPTKRALLIAAMRAQRPCQMKGYFFEASMATFSTIVRSAVSYIMMLRSFN
A

>DmOr10a

MSEWLRFLKRDQQLDVYFFAVPRLSLDIMGYWPGKTGDTWPWRSLIHFAILAIGVATELHAGMCFLDRQQITLAL
ETLCPAGTSAVTLLKMFLMLRFRQDLSIMWNRLRGLLFDPNWERPEQRDIRLKHSAMAARINFWPLSAGFFTCTTY
NLKPILIAMILYLQNRYEDFVWFTPFNMTMPKVLLNYPFFPLTYIFIAYTGYVTIFMFGGCDGFYFEFCAHLSALFEVL

QAEIESMFRPYTDHLELSPVQLYILEQKMRSVIIRHNAIIDLTRFFRDRYTIITLAHFVSAAMVIGFSMVNLLTLGNNGL
GAMLYVAYTVAALSQLLVYCYGGTLVAESSTGLCRAMFSCPWQLFKPKQRRLVQLLILRSQRPVSMAVPFFSPSLAT
FAAILQTSGSIIALVKSFQ

>DmOr13a

MFYSYPYKALSFPIQCVWLKLNGSWPLTESSRPWRSQSLLATAYIVWAWYVIASVGITISYQTAFLLNNLSDIIITTEN
CCTTFMGVLNFVRLIHLRLNQRKFRQLIENFSYEIWIPNSSKNNVAAECRRRMVTFSIMTSLLACLIIMYCVLPLVEIFF
GPAFDAQNKPFPYKMIFPYDAQSSWIRYVMTYIFTSYAGICVVTTLFAEDTILGFFITYTCGQFHLLHQRIAGLFAGSN
AELAESIQLERLKRIVEKHNNIISFAKRLEDFFNPILLANLMISSVLICMVGFQIVTGKNMFIGDYVKFIIYISSALSQLYVL
CENGDALIKQSTLTAQILYECQWEGSDRIEIQSFTPTTKRIRNQIWFMILCSQQPVRITAFKFSTLSLQSFTAILSTSISYF
TLLRSVYFDDEKKLD

>DmOr19a

MDISKVDSTRALVNHWRIFRIMGIHPPGKRTFWGRHYTAYSMVWNVTFHICIWVSFSVNLLQSNSLETFCESLCVT
MPHTLYMLKLINVRRMRGQMISSHWLLRLLDKRLGCDDERQIIMAGIERAEFIFRTIFRGLACTVVLGIIYISASSEPTL
MYPTWIPWNWRDSTSAYLATAMLHTTALMANATLVLNLSSYPGTYLILVSVHTKALALRVSKLGYGAPLPAVRMQ
AILVGYIHDHQIILRLFKSLERSLSMTCFLQFFSTACAQCTICYFLLFGNVGIMRFMNMLFLLVILTTETLLLCYTAELPCK
EGESLLTAVYSCNWLSQSVNFRRLLLLMLARCQIPMILVSGVIVPISMKTFTVMIKGAYTMLTLLNEIRKTSLE

>DmOr19b

MDISKVDSTRALVNHWRIFRIMGIHPPGKRTFWGRHYTAYSMVWNVTFHICIWVSFSVNLLQSNSLETFCESLCVT
MPHTLYMLKLINVRRMRGEMISSHWLLRLLDKRLGCADERQIIMAGIERAEFIFRTIFRGLACTVVLGIIYISASSEPTL
MYPTWIPWNWKDSTSAYLATAMLHTTALMANATLVLNLSSYPGTYLILVSVHTKALALRVSKLGYGAPLPAVRMQ
AILVGYIHDHQIILRLFKSLERSLSMTCFLQFFSTACAQCTICYFLLFGNVGIMRFMNMLFLLVILTTETLLLCYTAELPCK
EGESLLTAVYSCNWLSQSVNFRRLLLLMLARCQIPMILVSGVIVPISMKTFTVMIKGAYTMLTLLNEIRKTSLE

>DmOr22a

MLSKFFPHIKEKPLSERVKSRDAFIYLDRVMWSFGWTEPENKRWILPYKLWLAFVNIVMLILLPISISIEYLHRFKTFSA
GEFLSSLEIGVNMYGSSFKCAFTLIGFKKRQEAKVLLDQLDKRCLSDKERSTVHRYVAMGNFFDILYHIFYSTFVVMN
FPYFLLERRHAWRMYFPYIDSDEQFYISSIAECFLMTEAIYMDLCTDVCPLISMLMARCHISLLKQRLRNLRSKPGRTE
DEYLEELTECIRDHRLLLDYVDALRPVFSGTIFVQFLLIGTVLGLSMINLMFFSTFWTGVATCLFMFDVSMETFPFCYL
CNMIIDDCQEMSNCLFQSDWTSADRRYKSTLVYFLHNLQQPITLTAGGVFPISMQTNLAMVKLAFSVVTVIKQFNL
AERFQ

>DmOr22b

MLSQFFPHIKEKPLSERVKSRDAFVYLDRVMWSFGWTVPENKRWDLHYKLWSTFVTLLIFILLPISVSVEYIQRFKTFS
AGEFLSSIQIGVNMYGSSFKSYLTMMGYKKRQEAKMSLDELDKRCVCDEERTIVHRHVALGNFCYIFYHIAYTSFLIS
NFLSFIMKRIHAWRMYFPYVDPEKQFYISSIAEVILRGWAVFMDLCTDVCPLISMVIARCHITLLKQRLRNLRSEPGR
TEDEYLKELADCVRDHRLILDYVDALRSVFSGTIFVQFLLIGIVLGLSMINIMFFSTLSTGVAVVLFMSCVSMQTFPFCY
LCNMIMDDCQEMADSLFQSDWTSADRRYKSTLVYFLHNLQQPIILTAGGVFPISMQTNLNMVKLAFTVVTIVKQF
NLAEKFQ

>DmOr22c

MTDSGQPAIADHFYRIPRISGLIVGLWPQRIRGGGGRPWHAHLLFVFAFAMVVVGAVGEVSYGCVHLDNLVVALE
AFCPGTTKAVCVLKLWVFFRSNRRWAELVQRLRAILWESRRQEAQRMLVGLATTANRLSLLLLSSGTATNAAFTLQ
PLIMGLYRWIVQLPGQTELPFNIILPSFAVQPGVFPLTYVLLTASGACTVFAFSFVDGFFICSCLYICGAFRLVQQDIRRI
FADLHGDSVDVFTEEMNAEVRHRLAQVVERHNAIIDFCTDLTRQFTVIVLMHFLSAAFVLCSTILDIMLNTSSLSGLT
YICYIIAALTQLFLYCFGGNHVSESSAAVADVLYDMEWYKCDARTRKVILMILRRSQRAKTIAVPFFTPSLPALRSILST
AGSYITLLKTFL

>DmOr23a

MKLSETLKIDYFRVQLNAWRICGALDLSEGRYWSWSMLLCILVYLPTPMLLRGVYSFEDPVENNFSLSLTVTSLSNL
MKFCMYVAQLTKMVEVQSLIGQLDARVSGESQSERHRNMTEHLLRMSKLFQITYAVVFIIAAVPFVFETELSLPMP
MWFPFDWKNSMVAYIGALVFQEIGYVFQIMQCFAADSFPPLVLYLISEQCQLLILRISEIGYGYKTLEENEQDLVNCI
RDQNALYRLLDVTKSLVSYPMMVQFMVIGINIAITLFVLIFYVETLYDRIYYLCFLLGITVQTYPLCYYGTMVQESFAEL
HYAVFCSNWVDQSASYRGHMLILAERTKRMQLLLAGNLVPIHLSTYVACWKGAYSFFTLMADRDGLGS

>DmOr24a

MERHYFMVPKFALSLIGFYPEQKRTVLVKLWSFFNFFILTYGCYAEAYYGIHYIPINIATALDALCPVASSILSLVKMVAI
WWYQDELRSLIERVRFLTEQQKSKRKLGYKKRFYTLATQLTFLLLCCGFCTSTSYSVRHLIDNILRRTHGKDWIYETPF
KMMFPDLLLRLPLYPITYILVHWHGYITVVCFVGADGFFLGFCLYFTVLLLCLQDDVCDLLEVENIEKSPSEAEEARIVR
EMEKLVDRHNEVAELTERLSGVMVEITLAHFVTSSLIIGTSVVDILLFSGLGIIVYVVYTCAVGVEIFLYCLGGSHIMEAC
SNLARSTFSSHWYGHSVRVQKMTLLMVARAQRVLTIKIPFFSPSLETLTSILRFTGSLIALAKSVI

>DmOr30a

MELKSMDPVEMPIFGSTLKLMKFWSYLFVHNWRRYVAMTPYIIINCTQYVDIYLSTESLDFIIRNVYLAVLFTNTVVR
GVLLCVQRFSYERFINILKSFYIELLQSDDPIINILVKETTRLSVLISRINLLMGCCTCIGFVTYPIFGSERVLPYGMYLPTID
EYKYASPYYEIFFVIQAIMAPMGCCMYIPYTNMVVTFTLFAILMCRVLQHKLRSLEKLKNEQVRGEIIWCIKYQLKLSG
FVDSMNALNTHLHLVEFLCFGAMLCVLLFSLIIAQTIAQTVIVIAYMVMIFANSVVLYYVANELYFQSFDIAIAAYESN
WMDFDVDTQKTLKFLIMRSQKPLAILVGGTYPMNLKMLQSLLNAIYSFFTLLRRVYG

>DmOr33a

MDSRRKVRSENLYKTYWLYWRLLGVEGDYPFRRLVDFTITSFITILFPVHLILGMYKKPQIQVFRSLHFTSECLFCSYKF
FCFRWKLKEIKTIEGLLQDLDSRVESEEERNYFNQNPSRVARMLSKSYLVAAISAIITATVAGLFSTGRNLMYLGWFPY
DFQATAAIYWISFSYQAIGSSLLILENLANDSYPPITFCVVSGHVRLLIMRLSRIGHDVKLSSSENTRKLIEGIQDHRKL
MKIIRLLRSTLHLSQLGQFLSSGINISITLINILFFAENNFAMLYYAVFFAAMLIELFPSCYYGILMTMEFDKLPYAIFSSN
WLKMDKRYNRSLIILMQLTLVPVNIKAGGIVGIDMSAFFATVRMAYSFYTLALSFRV

>DmOr33b

MDLKPRVIRSEDIYRTYWLYWHLLGLESNFFLNRLLDLVITIFVTIWYPIHLILGLFMERSLGDVCKGLPITAACFFASFK
FICFRFKLSEIKEIEILFKELDQRALSREECEFFNQNTRREANFIWKSFIVAYGLSNISAIASVLFGGGHKLLYPAWFPYD
VQATELIFWLSVTYQIAGVSLAILQNLANDSYPPMTFCVVAGHVRLLAMRLSRIGQGPEETIYLTGKQLIESIEDHRKL
MKIVELLRSTMNISQLGQFISSGVNISITLVNILFFADNNFAITYYGVYFLSMVLELFPCCYYGTLISVEMNQLTYAIYSS
NWMSMNRSYSRILLIFMQLTLAEVQIKAGGMIGIGMNAFFATVRLAYSFFTLAMSLR

>DmOr33c

MVIIDSLSFYRPFWICMRLLVPTFFKDSSRPVQLYVVLLHILVTLWFPLHLLLHLLLLPSTAEFFKNLTMSLTCVACSLKH
VAHLYHLPQIVEIESLIEQLDTFIASEQEHRYYRDHVHCHARRFTRCLYISFGMIYALFLFGVFVQVISGNWELLYPAYF
PFDLESNRFLGAVALGYQVFSMLVEGFQGLGNDTYTPLTLCLLAGHVHLWSIRMGQLGYFDDETVVNHQRLLDYIE
QHKLLVRFHNLVSRTISEVQLVQLGGCGATLCIIVSYMLFFVGDTISLVYYLVFFGVVCVQLFPSCYFASEVAEELERLP
YAIFSSRWYDQSRDHRFDLLIFTQLTLGNRGWIIKAGGLIELNLNAFFATLKMAYSLFAVVVRAKGI

>DmOr35a

MVRYVPRFADGQKVKLAWPLAVFRLNHIFWPLDPSTGKWGRYLDKVLAVAMSLVFMQHNDAELRYLRFEASNR
NLDAFLTGMPTYLILVEAQFRSLHILLHFEKLQKFLEIFYANIYIDPRKEPEMFRKVDGKMIINRLVSAMYGAVISLYLIA
PVFSIINQSKDFLYSMIFPFDSDPLYIFVPLLLTNVWVGIVIDTMMFGETNLLCELIVHLNGSYMLLKRDLQLAIEKILV
ARDRPHMAKQLKVLITKTLRKNVALNQFGQQLEAQYTVRVFIMFAFAAGLLCALSFKAYTNPMANYIYAIWFGAKT
VELLSLGQIGSDLAFTTDSLSTMYYLTHWEQILQYSTNPSENLRLLKLINLAIEMNSKPFYVTGLKYFRVSLQAGLKILQ
ASFSYFTFLTSMQRRQMSN

>DmOr42a

MDLRRWFPTLYTQSKDSPVRSRDATLYLLRCVFLMGVRKPPAKFFVAYVLWSFALNFCSTFYQPIGFLTGYISHLSEF
SPGEFLTSLQVAFNAWSCSTKVLIVWALVKRFDEANNLLDEMDRRITDPGERLQIHRAVSLSNRIFFFFMAVYMVY
ATNTFLSAIFIGRPPYQNYYPFLDWRSSTLHLALQAGLEYFAMAGACFQDVCVDCYPVNFVLVLRAHMSIFAERLRR
LGTYPYESQEQKYERLVQCIQDHKVILRFVDCLRPVISGTIFVQFLVVGLVLGFTLINIVLFANLGSAIAALSFMAAVLLE
TTPFCILCNYLTEDCYKLADALFQSNWIDEEKRYQKTLMYFLQKLQQPITFMAMNVFPISVGTNISVTKFSFSVFTLVK
QMNISEKLAKSEMEE

>DmOr42b

MVFELIRPAPLTEQKRSRDGCIYLYRAMKFIGWLPPKQGVLRYVYLTWTLMTFVWCTTYLPLGFLGSYMTQIKSFSP
GEFLTSLQVCINAYGSSVKVAITYSMLWRLIKAKNILDQLDLRCTAMEEREKIHLVVARSNHAFLIFTFVYCGYAGSTY
LSSVLSGRPPWQLYNPFIDWHDGTLKLWVASTLEYMVMSGAVLQDQLSDSYPLIYTLILRAHLDMLRERIRRLRSDE
NLSEAESYEELVKCVMDHKLILRYCAIIKPVIQGTIFTQFLLIGLVLGFTLINVFFFSDIWTGIASFMFVITILLQTFPFCYT
CNLIMEDCESLTHAIFQSNWVDASRRYKTTLLYFLQNVQQPIVFIAGGIFQISMSSNISVAKFAFSVITITKQMNIADK
FKTD

>DmOr43a

MTIEDIGLVGINVRMWRHLAVLYPTPGSSWRKFAFVLPVTAMNLMQFVYLLRMWGDLPAFILNMFFFSAIFNAL
MRTWLVIIKRRQFEEFLGQLATLFHSILDSTDEWGRGILRRAEREARNLAILNLSASFLDIVGALVSPLFREERAHPFGL
ALPGVSMTSSPVYEVIYLAQLPTPLLLSMMYMPFVSLFAGLAIFGKAMLQILVHRLGQIGGEEQSEEERFQRLASCIA
YHTQVMRYVWQLNKLVANIVAVEAIIFGSIICSLLFCLNIITSPTQVISIVMYILTMLYVLFTYYNRANEICLENNRVAEA
VYNVPWYEAGTRFRKTLLIFLMQTQHPMEIRVGNVYPMTLAMFQSLLNASYSYFTMLRGVTGK

>DmOr43b

MFGHFKLVYPAPISEPIQSRDSNAYMMETLRNSGLNLKNDFGIGRKIWRVFSFTYNMVILPVSFPINYVIHLAEFPPE
LLLQSLQLCLNTWCFALKFFTLIVYTHRLELANKHFDELDKYCVKPAEKRKVRDMVATITRLYLTFVVVYVLYATSTLLD
GLLHHRVPYNTYYPFINWRVDRTQMYIQSFLEYFTVGYAIYVATATDSYPVIYVAALRTHILLLKDRIIYLGDPSNEGSS
DPSYMFKSLVDCIKAHRTMLNFCDAIQPIISGTIFAQFIICGSILGIIMINMVLFADQSTRFGIVIYVMAVLLQTFPLCFY

CNAIVDDCKELAHALFHSAWWVQDKRYQRTVIQFLQKLQQPMTFTAMNIFNINLATNINVAKFAFTVYAIASGMN
LDQKLSIKE

>DmOr45a

MDASYFAVQRRALEIVGFDPSTPQLSLKHPIWAGILILSLISHNWPMVVYALQDLSDLTRLTDNFAVFMQGSQSTFK
FLVMMAKRRRIGSLIHRLHKLNQAASATPNHLEKIERENQLDRYVARSFRNAAYGVICASAIAPMLLGLWGYVETG
VFTPTTPMEFNFWLDERKPHFYWPIYVWGVLGVAAAAWLAIATDTLFSWLTHNVVIQFQLLELVLEEKDLNGGDS
RLTGFVSRHRIALDLAKELSSIFGEIVFVKYMLSYLQLCMLAFRFSRSGWSAQVPFRATFLVAIIIQLSSYCYGGEYIKQ
QSLAIAQAVYGQINWPEMTPKKRRLWQMVIMRAQRPAKIFGFMFVVDLPLLLWVIRTAGSFLAMLRTFER

>DmOr45b

MYPRFLSRNYPLAKHLFFVTRYSFGLLGLRFGKEQSWLHLLWLVFNFVNLAHCCQAEFVFGWSHLRTSPVDAMDA
FCPLACSFTTLFKLGWMWWRRQEVADLMDRIRLLIGEQEKREDSRRKVAQRSYYLMVTRCGMLVFTLGSITTGAF
VLRSLWEMWVRRHQEFKFDMPFRMLFHDFAHRMPWFPVFYLYSTWSGQVTVYAFAGTDGFFFGFTLYMAFLLQ
ALRYDIQDALKPIRDPSLRESKICCQRLADIVDRHNEIEKIVKEFSGIMAAPTFVHFVSASLVIATSVIDILLYSGYNIIRYV
VYTFTVSSAIFLYCYGGTEMSTESLSLGEAAYSSAWYTWDRETRRRVFLIILRAQRPITVRVPFFAPSLPVFTSVIKFTGS
IVALAKTIL

>DmOr46aA

MSKGVEIFYKGQKAFLNILSLWPQIERRWRIIHQVNYVHVIVFWVLLFDLLLVLHVMANLSYMSEVVKAIFILATSAG
HTTKLLSIKANNVQMEELFRRLDNEEFRPRGANEELIFAAACERSRKLRDFYGALSFAALSMILIPQFALDWSHLPLKT
YNPLGENTGSPAYWLLYCYQCLALSVSCITNIGFDSLCSSLFIFLKCQLDILAVRLDKIGRLITTSGGTVEQQLKENIRYH
MTIVELSKTVERLLCKPISVQIFCSVLVLTANFYAIAVLSDERLELFKYVTYQACMLIQIFILCYYAGEVTQRSLDLPHELY
KTSWVDWDYRSRRIALLFMQRLHSTLRIRTLNPSLGFDLMLFSSIVNCSYSYFALLKRVNS

>DmOr46aB

MVTEDFYKYQVWYFQILGVWQLPTWAADHQRRFQSMRFGFILVILFIMLLLFSFEMLNNISQVREILKVFFMFATEI
SCMAKLLHLKLKSRKLAGLVDAMLSPEFGVKSEQEMQMLELDRVAVVRMRNSYGIMSLGAASLILIVPCFDNFGEL
PLAMLEVCSIEGWICYWSQYLFHSICLLPTCVLNITYDSVAYSLLCFLKVQLQMLVLRLEKLGPVIEPQDNEKIAMELR
ECAAYYNRIVRFKDLVELFIKGPGSVQLMCSVLVLVSNLYDMSTMSIANGDAIFMLKTCIYQLVMLWQIFIICYASNE
VTVQSSRLCHSIYSSQWTGWNRANRRIVLLMMQRFNSPMLLSTFNPTFAFSLEAFGSIVNCSYSYFALLKRVNS

>DmOr47a

MDSFLQVQKSTIALLGFDLFSENREMWKRPYRAMNVFSIAAIFPFILAAVLHNWKNVLLLADAMVALLITILGLFKFS
MILYLRRDFKRLIDKFRLLMSNEAEQGEEYAEILNAANKQDQRMCTLFRTCFLLAWALNSVLPLVRMGLSYWLAGH
AEPELPFPCLFPWNIHIIRNYVLSFIWSAFASTGVVLPAVSLDTIFCSFTSNLCAFFKIAQYKVVRFKGGSLKESQATLNK
VFALYQTSLDMCNDLNQCYQPIICAQFFISSLQLCMLGYLFSITFAQTEGVYYASFIATIIIQAYIYCYCGENLKTESASF
EWAIYDSPWHESLGAGGASTSICRSLLISMMRAHRGFRITGYFFEANMEAFSSIVRTAMSYITMLRSFS

>DmOr47b

MNDSGYQSNLSLLRVFLDEFRSVLRQESPGLIPRLAFYYVRAFLSLLCQYPNKKLASLPLYRWINLFIMCNVMTIFWT
MFVALPESKNVIEMGDDLVWISGMALVFTKIFYMHLRCDEIDELISDFEYYNRELRPHNIDEEVLGWQRLCYVIESGL
YINCFCLVNFFSAAIFLQPLLGEGKLPFHSVYPFQWHRLDLHPYTFWFLYIWQSLTSQHNLMSILMVDMVGISTFLQ

TALNLKLLCIEIRKLGDMEVSDKRFHEEFCRVVRFHQHIIKLVGKANRAFNGAFNAQLMASFSLISISTFETMAAAAV
DPKMAAKFVLLMLVAFIQLSLWCVSGTLVYTQSVEVAQAAFDINDWHTKSPGIQRDISFVILRAQKPLMYVAEPFLP
FTLGTYMLVLKNCYRLLALMQESM

>DmOr49a

MEKLRSYEDFIFMANMMFKTLGYDLFHTPKPWWRYLLVRGYFVLCTISNFYEASMVTTRIIEWESLAGSPSKIMRQ
GLHFFYMLSSQLKFITFMINRKRLLQLSHRLKELYPHKEQNQRKYEVNKYYLSCSTRNVLYVYYFVMVVMALEPLVQ
SCIMYLIGFGKADFTYKRIFPTRLTFDSEKPLGYVLAYVIDFTYSQFIVNVSLGTDLWMMCVSSQISMHLGYLANMLA
SIRPSPETEQQDCDFLASIIKRHQLMIRLQKDVNYVFGLLLASNLFTTSCLLCCMAYYTVVEGFNWEGISYMMLFASV
AAQFYVVSSHGQMLIDLSTNLAKAAFESKWYEGSLRYKKEILILMAQAQRPLEISARGVIIISLDTFKILMTITYRFFAVI
RQTVEK

>DmOr49b

MFEDIQLIYMNIKILRFWALLYDKNLRRYVCIGLASFHIFTQIVYMMSTNEGLTGIIRNSYMLVLWINTVLRAYLLLAD
HDRYLALIQKLTEAYYDLLNLNDSYISEILDQVNKVGKLMARGNLFFGMLTSMGFGLYPLSSSERVLPFGSKIPGLNEY
ESPYYEMWYIFQMLITPMGCCMYIPYTSLIVGLIMFGIVRCKALQHRLRQVALKHPYGDRDPRELREEIIACIRYQQSI
IEYMDHINELTTMMFLFELMAFSALLCALLFMLIIVSGTSQLIIVCMYINMILAQILALYWYANELREQNLAVATAAYE
TEWFTFDVPLRKNILFMMMRAQRPAAILLGNIRPITLELFQNLLNTTYTFFTVLKRVYG

>DmOr56a

MFKVKDLLLSPTTFEDPIFGTHLRYFQWYGYVASKDQNRPLLSLIRCTILTASIWLSCALMLARVFRGYENLNDGATS
YATAVQYFAVSIAMFNAYVQRDKVISLLRVAHSDIQNLMHEADNREMELLVATQAYTRTITLLIWIPSVIAGLMAYS
DCIYRSLFLPKSVFNVPAVRRGEEHPILLFQLFPFGELCDNFVVGYLGPWYALGLGITAIPLWHTFITCLMKYVNLKLQI
LNKRVEEMDITRLNSKLVIGRLTASELTFWQMQLFKEFVKEQLRIRKFVQELQYLICVPVMADFIIFSVLICFLFFALTV
GVPSKMDYFFMFIYLFVMAGILWIYHWHATLIVECHDELSLAYFSCGWYNFEMPLQKMLVFMMMHAQRPMKM
RALLVDLNLRTFIDIGRGAYSYFNLLRSSHLY

>DmOr59a

MAEVRVDSLEFFKSHWTAWRYLGVAHFRVENWKNLYVFYSIVSNLLVTLCYPVHLGISLFRNRTITEDILNLTTFATC
TACSVKCLLYAYNIKDVLEMERLLRLLDERVVGPEQRSIYGQVRVQLRNVLYVFIGIYMPCALFAELSFLFKEERGLMY
PAWFPFDWLHSTRNYYIANAYQIVGISFQLLQNYVSDCFPAVVLCLISSHIKMLYNRFEEVGLDPARDAEKDLEACIT
DHKHILELFRRIEAFISLPMLIQFTVTALNVCIGLAALVFFVSEPMARMYFIFYSLAMPLQIFPSCFFGTDNEYWFGRL
HYAAFSCNWHTQNRSFKRKMMLFVEQSLKKSTAVAGGMMRIHLDTFFSTLKGAYSLFTIIIRMRK

>DmOr59b

MAVFKLIKPAPLTEKVQSRQGNIYLYRAMWLIGWIPPKEGVLRYVYLFWTCVPFAFGVFYLPVGFIISYVQEFKNFTP
GEFLTSLQVCINVYGASVKSTITYLFLWRLRKTEILLDSLDKRLANDSDRERIHNMVARCNYAFLIYSFIYCGYAGSTFLS
YALSGRPPWSVYNPFIDWRDGMGSLWIQAIFEYITMSFAVLQDQLSDTYPLMFTIMFRAHMEVLKDHVRSLRMD
PERSEADNYQDLVNCVLDHKTILKCCDMIRPMISRTIFVQFALIGSVLGLTLVNVFFFSNFWKGVASLLFVITILLQTFP
FCYTCNMLIDDAQDLSNEIFQSNWVDAEPRYKATLVLFMHHVQQPIIFIAGGIFPISMNSNITVAKFAFSIITIVRQM
NLAEQFQ

>DmOr59c

MTKFFFKRLQTAPLDQEVSSLDASDYYYRIAFFLGWTPPKGALLRWIYSLWTLTTMWLGIVYLPLGLSLTYVKHFDRF
TPTEFLTSLQVDINCIGNVIKSCVTYSQMWRFRRMNELISSLDKRCVTTTQRRIFHKMVARVNLIVILFLSTYLGFCFLT
LFTSVFAGKAPWQLYNPLVDWRKGHWQLWIASILEYCVVSIGTMQELMSDTYAIVFISLFRCHLAILRDRIANLRQD
PKLSEMEHYEQMVACIQDHRTIIQCSQIIRPILSITIFAQFMLVGIDLGLAAISILFFPNTIWTIMANVSFIVAICTESFPC
CMLCEHLIEDSVHVSNALFHSNWITADRSYKSAVLYFLHRAQQPIQFTAGSIFPISVQSNIAVAKFAFTIITIVNQMNL
GEKFFSDRSNGDINP

>DmOr63a

MYSPEEAAELKRRNYRSIREMIRLSYTVGFNLLDPSRCGQVLRIWTIVLSVSSLASLYGHWQMLARYIHDIPRIGETA
GTALQFLTSIAKMWYFLFAHRQIYELLRKARCHELLQKCELFERMSDLPVIKEIRQQVESTMNRYWASTRRQILIYLYS
CICITTNYFINSFVINLYRYFTKPKGSYDIMLPLPSLYPAWEHKGLEFPYYHIQMYLETCSLYICGMCAVSFDGVFIVLCL
HSVGLMRSLNQMVEQATSELVPPDRRVEYLRCCIYQYQRVANFATEVNNCFRHITFTQFLLSLFNWGLALFQMSV
GLGNNSSITMIRMTMYLVAAGYQIVVYCYNGQRFATASEEIANAFYQVRWYGESREFRHLIRMMLMRTNRGFRL
DVSWFMQMSLPTLMAMVRTSGQYFLLLQNVNQK

>DmOr65a

MTELRSERKNGNWDRLFGPFFESWAVFKAPQAKSRHIIAYWTRDQLKALGFYMNSEQRRLPRIVAWQYFVSIQLA
TALASLFYGISESIGDIVNLGRDLVFIITIIFICFRLVFFAQYAGELDVIIDALEDIYHWSIKGPATKEVQETKRLHFLLFMA
LIITWFSFLILFMLIKISTPFWIESQTLPFHVSWPFQLHDPSKHPIAYIIIFVSQSTTMLYFLIWLGVVENMGVSLFFELTS
ALRVLCIELRNLQELCLGDEDMLYRELCRMTKFHQQIILLTDRCNHIFNGAFIMQMLINFLLVSLSLFEVLAAKKNPQV
AVEYMIIMLMTLGHLSFWSKFGDMFSKESEQVALAVYEAYDPNVGSKSIHRQFCFFIQRAQKPLIMKASPFPPFNLE
NYMFILKQCYSILTILANTLE

>DmOr65b

MEASHSSIYYWREQMKAMALFTTTEERLLPYRSKWHTLVYIQMVIFFASMSFGLTESMGDHVQMGRDLAFILGAF
FIIFKTYYFCWYGDELDQVISDLDALHPWAQKGPNPVEYQTGKRWYFVMAFFLATSWSFFLCILLLLLITSPMWVH
QQNLPFHAAFPFQWHEKSLHPISHAIIYLFQSYFAVYCLTWLLCIEGLSICIYAEITFGIEVLCLELRQIHRHNYGLQELR
METNRLVKLHQKIVEILDRTNDVFHGTLIMQMGVNFSLVSLSVLEAVEARKDPKVVAQFAVLMLLALGHLSMWSY
CGDQLSQKSLQISEAAYEAYDPTKGSKDVYRDLCVIIRRGQDPLIMRASPFPSFNLINYSAILNQCYGILTFLLKTLD

>DmOr65c

MESSYSAVYYWREQMKAMFLYTTSKERQMPYRSSWHTLVIIQATVCFLTMCYGVTESLGDKVQMGRDIAFIIGFFY
IAFKIYYFQWYGDELDEVVEALETFHPWAQKGPGAVDYRTAKRWYFTLAFFLASSWLVFLCIFILLLITSPLWVHQQI
LPLHAAFPFQWHEKSIHPISHAFIYLFQTWNVMYFLTWLVCIEGLSVSIYVEITFAIEVLCLELRHLHQRCHGYEQLRLE
TNRLVQFHQKIVHILDHTNKVFHGTLIMQMGVNFFLVSLSVLEAMEARKDPKVVAQFAVLMLLALGHLSMWSYF
GDLLSQKSLTISEAAYEAYDPIKGSKDVYRDLCLIIRRGQEPLIMRASPFPSFNFINYSAILNQCYGILTFLLKTLD

>DmOr67a

MDNVAEMPEEKYVEVDDFLRLAVKFYNTLGIDPYETGRKRTIWFQIYFALNMFNMVFSFYAEVATLVDRLRDNENF
LESCILLSYVSFVVMGLSKIGAVMKKKPKMTALVRQLETCFPSPSAKVQEEYAVKSWLKRCHIYTKGFGGLFMIMYF
AHALIPLFIYFIQRVLLHYPDAKQIMPFYQLEPWEFRDSWLFYPSYFHQSSAGYTATCGSIAGDLMIFAVVLQVIMHY
ERLAKVLREFKIQAHNAPNGAKEDIRKLQSLVANHIDILRLTDLMNEVFGIPLLLNFIASALLVCLVGVQLTIALSPEYFC

KQMLFLISVLLEVYLLCSFSQRLIDASENVGHAAYDMDWLGSDKRFKKILIFISMRSQKPVCLKATVVLDLSMPTMSI
FLGMSYKFFCAVRTMYQ

>DmOr67b

MQDQLDHELERIDKLPKLGLLWVEYSAYALGVNIAPRKRSSKYCRLTRILVLIVNLSIIYSLVAFIMENYMISFETYVEA
VLLTFQLSVGVVKMFHFQNKVESCSQLVFSTETGEVLKSLGLFQLDLPRKKELLSSVSLILLNNWMIIDRQVMFFFKIV
CMPVLYYCVRPYFQYIFDCYIKDKDTCEMTLTYPAIVPYLQLGNYEFPSYVIRFFLLQSGPLWCFFAVFGFNSLFVVLT
RYESGLIKVLRFLVQNSTSDILVPKDQRVKYLQCCVRLFARISSHHNQIENLFKYIILVQCSVSSILICMLLYKISTVLEVG
WVWMGMIMVYFVTIALEITLYNVSAQKVESQSELLFHDWYNCSWYNESREFKFMIKMMLLFSRRTFVLSVGGFTS
LSHKFLVQVFRLSANFFLLLRNMNNK

>DmOr67c

METAKDNTARTFMELMRVPVQFYRTIGEDIYAHRSTNPLKSLLFKIYLYAGFINFNLLVIGELVFFYNSIQDFETIRLAIA
VAPCIGFSLVADFKQAAMIRGKKTLIMLLDDLENMHPKTLAKQMEYKLPDFEKTMKRVINIFTFLCLAYTTTFSFYPAI
KASVKFNFLGYDTFDRNFGFLIWFPFDATRNNLIYWIMYWDIAHGAYLAGIAFLCADLLLVVVITQICMHFNYISMR
LEDHPCNSNEDKENIEFLIGIIRYHDKCLKLCEHVNDLYSFSLLLNFLMASMQICFIAFQVTESTVEVIIIYCIFLMTSMV
QVFMVCYYGDTLIAASLKVGDAAYNQKWFQCSKSYCTMLKLLIMRSQKPASIRPPTFPPISLVTYMKVISMSYQFFA
LLRTTYSNN

>DmOr67d

MLKMAKVEPVERYCKVIRMIRFCVGFCGNDVADPNFRMWWLTYAVMAAIAFFFACTGYTIYVGVVINGDLTIILQ
ALAMVGSAVQGLTKLLVTANNASHMREVQNTYEDIYREYGSKGDEYAKCLEKRIRITWTLLIGFMLVYIILLGLVITFP
IFYLLILHQKVLVMQFLIPFLDHTTDGGHLILTAAHVILITFGGFGNYGGDMYLFLFVTHVPLIKDIFCVKLTEFNELVM
KRNDFPKVRAMLCDLLVWHQLYTRMLQTTKKIYSIVLFVQLSTTCVGLLCTISCIFMKAWPAAPLYLLYAAITLYTFCG
LGTLVENSNEDFLSVIYTNCLWYELPVKEEKLIIMMLAKAQNEVVLTAADMAPLSMNTALQLTKGIYSFSMMLMNY
LG

>DmOr69aA

MQLHDHMKYIDLGCKMACIPRYQWKGRPTERQFYASEQRIVFLLGTICQIFQITGVLIYWYCNGRLATETGTFVAQ
LSEMCSSFCLTFVGFCNVYAISTNRNQIETLLEELHQIYPRYRKNHYRCQHYFDMAMTIMRIEFLFYMILYVYYNSAPL
WVLLWEHLHEEYDLSFKTQTNTWFPWKVHGSALGFGMAVLSITVGSFVGVGFSIVTQNLICLLTFQLKLHYDGISS
QLVSLDCRRPGAHKELSILIAHHSRILQLGDQVNDIMNFVFGSSLVGATIAICMSSVSIMLLDLASAFKYASGLVAFVL
YNFVICYMGTEVTLASGKVLPAAFYNNWYEGDLVYRRMLLILMMRATKPYMWKTYKLAPVSITTYMATLKFSYQM
FTCVRSLK

>DmOr69aB

MQLEDFMRYPDLVCQAAQLPRYTWNGRRSLEVKRNLAKRIIFWLGAVNLVYHNIGCVMYGYFGDGRTKDPIAYLA
ELASVASMLGFTIVGTLNLWKMLSLKTHFENLLNEFEELFQLIKHRAYRIHHYQEKYTRHIRNTFIFHTSAVVYYNSLPI
LLMIREHFSNSQQLGYRIQSNTWYPWQVQGSIPGFFAAVACQIFSCQTNMCVNMFIQFLINFFGIQLEIHFDGLAR
QLETIDARNPHAKDQLKYLIVYHTKLLNLADRVNRSFNFTFLISLSVSMISNCFLAFSMTMFDFGTSLKHLLGLLLFITY
NFSMCRSGTHLILTSGKVLPAAFYNNWYEGDLVYRRMLLILMMRATKPYMWKTYKLAPVSITTYMATLKFSYQMF
TCVRSLK

>DmOr71a

MDYDRIRPVRFLTGVLKWWRLWPRKESVSTPDWTNWQAYALHVPFTFLFVLLLWLEAIKSRDIQHTADVLLICLTT
TALGGKVINIWKYAHVAQGILSEWSTWDLFELRSKQEVDMWRFEHRRFNRVFMFYCLCSAGVIPFIVIQPLFDIPNR
LPFWMWTPFDWQQPVLLWYAFIYQATTIPIACACNVTMDAVNWYLMLHLSLCLRMLGQRLSKLQHDDKDLREK
FLELIHLHQRLKQQALSIEIFISKSTFTQILVSSLIICFTIYSMQMSPVLQDLPGFAAMMQYLVAMIMQVMLPTIYGNA
VIDSANMLTDSMYNSDWPDMNCRMRRLVLMFMVYLNRPVTLKAGGFFHIGLPLFTKTMNQAYSLLALLLNMNQ

>DmOr74a

MSFHRYRPRLPGGELAPMPWPVSLYRVLNHVAWPLEAESGRWTVFLDRLMIFLGFLVFCEHNEVDFHYLIANRQD
MDNMLTGLPTYLILVEMQIRCFQLAWHKDRFRALLQRFYAEIYVSEEMEPHLFASIQRQMLATRVNSTVYLLALLNF
FLVPVTNVIYHRREMLYKQVYPFDNTQLHFFIPLLVLNFWVGFIITSMLFGELNVMGELMMHLNARYIQLGQDLRR
SAQMLLKKSSSLNVAIAYRLNLTHILRRNAALRDFGQRVEKEFTLRIFVMFAFSAGLLCALFFKAFTNPWGNVAYIVW
FLAKFMELLALGMLGSILLKTTDELGMMYYTADWEQVIHQSDNVGENVKLMKLVTLAIQLNSRPFFITGLNYFRVSL
TAVLKIIQGAFSYFTFLNSMR

>DmOr82a

MGRLFQLQEYCLRAMGHKDDMDSTDSTALSLKHISSLIFVISAQYPLISYVAYNRNDMEKVTACLSVVFTNMLTVIKI
STFLANRKDFWEMIHRFRKMHEQSSHIPRYREGLDYVAEANKLASFLGRAYCVSCGLTGLYFMLGPIVKIGVCRWH
GTTCDKELPMPMKFPFNDLESPGYEVCFLYTVLVTVVVVAYASAVDGLFISFAINLRAHFQTLQRQIENWEFPSSEP
DTQIRLKSIVEYHVLLLSLSRKLRSIYTPTVMGQFVITSLQVGVIIYQLVTNMDSVMDLLLYASFFGSIMLQLFIYCYGGE
IIKAESLQVDTAVRLSNWHLASPKTRTSLSLIILQSQKEVLIRAGFFVASLANFVGICRTALSLITLIKSIE

>DmOr83a

MKSTFKEERIKDDSKRRDLFVFVRQTMCIAAMYPFGYYVNGSGVLAVLVRFCDLTYELFNYFVSVHIAGLYICTIYINY
GQGDLDFFVNCLIQTIIYLWTIAMKLYFRRFRPGLLNTILSNINDEYETRSAVGFSFVTMAGSYRMSKLWIKTYVYCCY
IGTIFWLALPIAYRDRSLPLACWYPFDYTQPGVYEVVFLLQAMGQIQVAASFASSSGLHMVLCVLISGQYDVLFCSLK
NVLASSYVLMGANMTELNQLQAEQSAADVEPGQYAYSVEEETPLQELLKVGSSMDFSSAFRLSFVRCIQHHRYIVA
ALKKIESFYSPIWFVKIGEVTFLMCLVAFVSTKSTAANSFMRMVSLGQYLLLVLYELFIICYFADIVFQNSQRCGEALW
RSPWQRHLKDVRSDYMFFMLNSRRQFQLTAGKISNLNVDRFRGTITTAFSFLTLLQKMDARE

>DmOr83b

MTTSMQPSKYTGLVADLMPNIRAMKYSGLFMHNFTGGSAFMKKVYSSVHLVFLLMQFTFILVNMALNAEEVNEL
SGNTITTLFFTHCITKFIYLAVNQKNFYRTLNIWNQVNTHPLFAESDARYHSIALAKMRKLFFLVMLTTVASATAWTTI
TFFGDSVKMVVDHETNSSIPVEIPRLPIKSFYPWNASHGMFYMISFAFQIYYVLFSMIHSNLCDVMFCSWLIFACEQL
QHLKGIMKPLMELSASLDTYRPNSAALFRSLSANSKSELIHNEEKDPGTDMDMSGIYSSKADWGAQFRAPSTLQSF
GGNGGGGNGLVNGANPNGLTKKQEMMVRSAIKYWVERHKHVVRLVAAIGDTYGAALLLHMLTSTIKLTLLAYQA
TKINGVNVYAFTVVGYLGYALAQVFHFCIFGNRLIEESSSVMEAAYSCHWYDGSEEAKTFVQIVCQQCQKAMSISG
AKFFTVSLDLFASVLGAVVTYFMVLVQLK

>DmOr83c

MSTSESPSSRFRELSKYINSLTNLLGVDFLSPKLKFNYRTWTTIFAIANYTGFTVFTILNNGGDWRVGLKASLMTGGLF
HGLGKFLTCLLKHQDMRRLVLYSQSIYDEYETRGDSYHRTLNSNIDRLLGIMKIIRNGYVFAFCLMELLPLAMLMYDG
TRVTAMQYLIPGLPLENNYCYVVTYMIQTVTMLVQGVGFYSGDLFVFLGLTQILTFADMLQVKVKELNDALEQKAE
YRALVRVGASIDGAENRQRLLLDVIRWHQLFTDYCRAINALYYELIATQVLSMALAMMLSFCINLSSFHMPSAIFFVV

SAYSMSIYCILGTILEFAYDQVYESICNVTWYELSGEQRKLFGFLLRESQYPHNIQILGVMSLSVRTALQIVKLIYSVSM
MMMNRA

>DmOr85a

MIFKYIQEPVLGSLFRSRDSLIYLNRSIDQMGWRLPPRTKPYWWLYYIWTLVVIVLVFIFIPYGLIMTGIKEFKNFTTTD
LFTYVQVPVNTNASIMKGIIVLFMRRRFSRAQKMMDAMDIRCTKMEEKVQVHRAAALCNRVVVIYHCIYFGYLSM
ALTGALVIGKTPFCLYNPLVNPDDHFYLATAIESVTMAGIILANLILDVYPIIYVVVLRIHMELLSERIKTLRTDVEKGDD
QHYAELVECVKDHKLIVEYGNTLRPMISATMFIQLLSVGLLLGLAAVSMQFYNTVMERVVSGVYTIAILSQTFPFCYV
CEQLSSDCESLTNTLFHSKWIGAERRYRTTMLYFIHNVQQSILFTAGGIFPICLNTNIKMAKFAFSVVTIVNEMDLAEK
LRRE

>DmOr85b

MEKLMKYASFFYTAVGIRPYTNGEESKMNKLIFHIVFWSNVINLSFVGLFESIYVYSAFMDNKFLEAVTALSYIGFVTV
GMSKMFFIRWKKTAITELINELKEIYPNGLIREERYNLPMYLGTCSRISLIYSLLYSVLIWTFNLFCVMEYWVYDKWLNI
RVVGKQLPYLMYIPWKWQDNWSYYPLLFSQNFAGYTSAAGQISTDVLLCAVATQLVMHFDFLSNSMERHELSGD
WKKDSRFLVDIVRYHERILRLSDAVNDIFGIPLLLNFMVSSFVICFVGFQMTVGVPPDIVVKLFLFLVSSMSQVYLICH
YGQLVADASYGFSVATYNQKWYKADVRYKRALVIIIARSQKVTFLKATIFLDITRSTMTDLLQISYKFFALLRTMYTQ

>DmOr85c

MKFMKYAVFFYTSVGIEPYTIDSRSKKASLWSHLLFWANVINLSVIVFGEILYLGVAYSDGKFIDAVTVLSYIGFVIVG
MSKMFFIWWKKTDLSDLVKELEHIYPNGKAEEEMYRLDRYLRSCSRISITYALLYSVLIWTFNLFSIMQFLVYEKLLKIR
VVGQTLPYLMYFPWNWHENWTYYVLLFCQNFAGHTSASGQISTDLLLCAVATQVVMHFDYLARVVEKQVLDRD
WSENSRFLAKTVQYHQRILRLMDVLNDIFGIPLLLNFMVSTFVICFVGFQMTVGVPPDIMIKLFLFLFSSLSQVYLICH
YGQLIADASSSLSISAYKQNWQNADIRYRRALVFFIARPQRTTYLKATIFMNITRATMTDLLQVSYKFFALLRTMYIK

>DmOr85d

MLTKKDTQSAKEQEKLKAIPLHSFLKYANVFYLSIGMMAYDHKYSQKWKEVLLHWTFIAQMVNLNTVLISELIYVFL
AIGKGSNFLEATMNLSFIGFVIVGDFKIWNISRQRKRLTQVVSRLEELHPQGLAQQEPYNIGHHLSGYSRYSKFYFGM
HMVLIWTYNLYWAVYYLVCDFWLGMRQFERMLPYYCWVPWDWSTGYSYYFMYISQNIGGQACLSGQLAADML
MCALVTLVVMHFIRLSAHIESHVAGIGSFQHDLEFLQATVAYHQSLIHLCQDINEIFGVSLLSNFVSSSFIICFVGFQM
TIGSKIDNLVMLVLFLFCAMVQVFMIATHAQRLVDASEQIGQAVYNHDWFRADLRYRKMLILIIKRAQQPSRLKAT
MFLNISLVTVSDLLQLSYKFFALLRTMYVN

>DmOr85e

MASLQFHGNVDADIRYDISLDPARESNLFRLLMGLQLANGTKPSPRLPKWWPKRLEMIGKVLPKAYCSMVIFTSLH
LGVLFTKTTLDVLPTGELQAITDALTMTIIYFFTGYGTIYWCLRSRRLLAYMEHMNREYRHHSLAGVTFVSSHAAFRM
SRNFTVVWIMSCLLGVISWGVSPLMLGIRMLPLQCWYPFDALGPGTYTAVYATQLFGQIMVGMTFGFGGSLFVTL
SLLLLGQFDVLYCSLKNLDAHTKLLGGESVNGLSSLQEELLLGDSKRELNQYVLLQEHPTDLLRLSAGRKCPDQGNAF
HNALVECIRLHRFILHCSQELENLFSPYCLVKSLQITFQLCLLVFVGVSGTREVLRIVNQLQYLGLTIFELLMFTYCGELLS
RHSIRSGDAFWRGAWWKHAHFIRQDILIFLVNSRRAVHVTAGKFYVMDVNRLRSVITQAFSFLTLLQKLAAKKTESE
L

>DmOr85f

MEPVQYSYEDFARLPTTVFWIMGYDMLGVPKTRSRRILYWIYRFLCLASHGVCVGVMVFRMVEAKTIDNVSLIMR
YATLVTYIINSDTKFATVLQRSAIQSLNSKLAELYPKTTLDRIYHRVNDHYWTKSFVYLVIIYIGSSIMVVIGPIITSIIAYFT
HNVFTYMHCYPYFLYDPEKDPVWIYISIYALEWLHSTQMVISNIGADIWLLYFQVQINLHFRGIIRSLADHKPSVKHD
QEDRKFIAKIVDKQVHLVSLQNDLNGIFGKSLLLSLLTTAAVICTVAVYTLIQGPTLEGFTYVIFIGTSVMQVYLVCYYG
QQVLDLSGEVAHAVYNHDFHDASIAYKRYLLIIIIRAQQPVELNAMGYLSISLDTFKQLMSVSYRVITMLMQMIQ

>DmOr88a

MKPTEIKKPYRMEEFLRPQMFQEVAQMVHFQWRRNPVDNSMVNASMVPFCLSAFLNVLFFGCNGWDIIGHFW
LGHPANQNPPVLSITIYFSIRGLMLYLKRKEIVEFVNDLDRECPRDLVSQLDMQMDETYRNFWQRYRFIRIYSHLGG
PMFCVVPLALFLLTHEGKDTPVAQHEQLLGGWLPCGVRKDPNFYLLVWSFDLMCTTCGVSFFVTFDNLFNVMQG
HLVMHLGHLARQFSAIDPRQSLTDEKRFFVDLRLLVQRQQLLNGLCRKYNDIFKVAFLVSNFVGAGSLCFYLFMLSE
TSDVLIIAQYILPTLVLVGFTFEICLRGTQLEKASEGLESSLRSQEWYLGSRRYRKFYLLWTQYCQRTQQLGAFGLIQVN
MVHFTEIMQLAYRLFTFLKSH

>DmOr92a

MLFRKRKPKSDDEVITFDELTRFPMTFYKTIGEDLYSDRDPNVIRRYLLRFYLVLGFLNFNAYVVGEIAYFIVHIMSTTT
LLEATAVAPCIGFSFMADFKQFGLTVNRKRLVRLLDDLKEIFPLDLEAQRKYNVSFYRKHMNRVMTLFTILCMTYTSS
FSFYPAIKSTIKYYLMGSEIFERNYGFHILFPYDAETDLTVYWFSYWGLAHCAYVAGVSYVCVDLLLIATITQLTMHFN
FIANDLEAYEGGDHTDEENIKYLHNLVVYHARALDLSEEVNNIFSFLILWNFIAASLVICFAGFQITASNVEDIGVYFIFF
SASLVQVFVVCYYGDEMISSSSRIGHSAFNQNWLPCSTKYKRILQFIIARSQKPASIRPPTFPPISFNTFMKVISMSYQF
FALLRTTYYG

>DmOr94a

MDKHKDRIESMRLILQVMQLFGLWPWSLKSEEEWTFTGFVKRNYRFLLHLPITFTFIGLMWLEAFISSNLEQAGQV
LYMSITEMALVVKILSIWHYRTEAWRLMYELQHAPDYQLHNQEEVDFWRREQRFFKWFFYIYILISLGVVYSGCTGV
LFLEGYELPFAYYVPFEWQNERRYWFAYGYDMAGMTLTCISNITLDTLGCYFLFHISLLYRLLGLRLRETKNMKNDTI
FGQQLRAIFIMHQRIRSLTLTCQRIVSPYILSQIILSALIICFSGYRLQHVGIRDNPGQFISMLQFVSVMILQIYLPCYYGN
EITVYANQLTNEVYHTNWLECRPPIRKLLNAYMEHLKKPVTIRAGNFFAVGLPIFVKTINNAYSFLALLLNVSN

>DmOr94b

MESTNRLSAIQTLLVIQRWIGLLKWENEGEDGVLTWLKRIYPFVLHLPLTFTYIALMWYEAITSSDFEEAGQVLYMSI
TELALVTKLLNIWYRRHEAASLIHELQHDPAFNLRNSEEIKFWQQNQRNFKRIFYWYIWGSLFVAVMGYISVFFQED
YELPFGYYVPFEWRTRERYFYAWGYNVVAMTLCCLSNILLDTLGCYFMFHIASLFRLLGMRLEALKNAAEEKARPEL
RRIFQLHTKVRRLTRECEVLVSPYVLSQVVFSAFIICFSAYRLVHMGFKQRPGLFVTTVQFVAVMIVQIFLPCYYGNEL
TFHANALTNSVFGTNWLEYSVGTRKLLNCYMEFLKRPVKVRAGVFFEIGLPIFVKTINNAYSFFALLLKISK

>DmOr98a

MLFNYLRKPNPTNLLTSPDSFRYFEYGMFCMGWHTPATHKIIYYITSCLIFAWCAVYLPIGIIISFKTDINTFTPNELLTV
MQLFFNSVGMPFKVLFFNLYISGFYKAKKLLSEMDKRCTTLKERVEVHQGVVRCNKAYLIYQFIYTAYTISTFLSAALS
GKLPWRIYNPFVDFRESRSSFWKAALNETALMLFAVTQTLMSDIYPLLYGLILRVHLKLLRLRVESLCTDSGKSDAENE
QDLIKCIKDHNLIIDYAAAIRPAVTRTIFVQFLLIGICLGLSMINLLFFADIWTGLATVAYINGLMVQTFPFCFVCDLLKK
DCELLVSAIFHSNWINSSRSYKSSLRYFLKNAQKSIAFTAGSIFPISTGSNIKVAKLAFSVVTFVNQLNIADRLTKN

>DmOr98b

MLTDKFLRLQSALFRLLGLELLHEQDVGHRYPWRSICCILSVASFMPLTIAFGLQNVQNVEQLTDSLCSVLVDLLALC
KIGLFLWLYKDFKFLIGQFYCVLQTETHTAVAEMIVTRESRRDQFISAMYAYCFITAGLSACLMSPLSMLISYHEQVNC
SRNFHFPVYPWDNMKLSNYIISYFWNVCAALGVALPTVCVDTLFCSLSHNLCALFQIARHKMMHFEGRNTKETHE
NLKHVFQLYALCLNLGHFLNEYFRPLICQFVAASLHLCVLCYQLSANILQPALLFYAAFTAAVVGQVSIYCFCGSSIHSE
CQLFGQAIYESSWPHLLQENLQLVSSLKIAMMRSSLGCPIDGYFFEANRETLITIVRTAISYVTLLRSLA
